# Dysregulated synaptic gene expression in oligodendrocytes of spinal and bulbar muscular atrophy

**DOI:** 10.1172/jci.insight.182123

**Published:** 2025-06-23

**Authors:** Madoka Iida, Kentaro Sahashi, Tomoki Hirunagi, Kenji Sakakibara, Kentaro Maeda, Yohei Iguchi, Jiayi Li, Yosuke Ogura, Masaki Iizuka, Tomohiro Akashi, Kunihiko Hinohara, Shouta Sugio, Hiroaki Wake, Masahiro Nakatochi, Masahisa Katsuno

**Affiliations:** 1 Department of Neurology, Nagoya University Graduate School of Medicine, Showa-ku, Nagoya, Aichi, Japan.; 2 Nagoya University Institute for Advanced Research, Furo-cho, Chikusa-ku, Nagoya, Aichi, Japan.; 3 Center for 5D Cell Dynamics,; 4 Division of Systems Biology, and; 5 Department of Immunology, Nagoya University Graduate School of Medicine, Nagoya, Aichi, Japan.; 6 Institute for Advanced Research, Nagoya University, Nagoya, Aichi, Japan.; 7 Department of Anatomy and Molecular Cell Biology, Nagoya University Graduate School of Medicine, Nagoya, Aichi, Japan.; 8 Division of Multicellular Circuit Dynamics, National Institute for Physiological Sciences, Okazaki, Aichi, Japan.; 9 Institutes of Innovation for Future Society, Nagoya University, Nagoya, Aichi, Japan.; 10 Department of Physiological Sciences, Graduate University for Advanced Studies, SOKENDAI, Hayama, Japan.; 11 Public Health Informatics Unit, Department of Integrated Health Sciences, and; 12 Department of Clinical Research Education, Nagoya University Graduate School of Medicine, Nagoya, Aichi, Japan.

**Keywords:** Cell biology, Neuroscience, Ion channels, Molecular biology, Neuromuscular disease

## Abstract

Spinal and bulbar muscular atrophy (SBMA) is a neuromuscular disease caused by an expanded CAG repeat in the androgen receptor (*AR*) gene. To elucidate the cell type–specific temporal gene expression in SBMA, we performed single-nucleus RNA sequencing on the spinal cords of an SBMA mouse model (AR-97Q). Among all cell types, oligodendrocytes had the highest number of differentially expressed genes before disease onset. Analysis of oligodendrocyte clusters suggested that pathways associated with cation channels and synaptic function were activated before disease onset, with increased output from oligodendrocytes to neurons in AR-97Q mice compared with wild-type mice. These changes in the early stages were abrogated at the advanced stages. An oligodendrocyte model of SBMA showed phenotypes similar to those of AR-97Q mice at early stages, such as increased transcriptional changes in synapse organization, and Ca^2+^ imaging of oligodendrocytes in AR-97Q mice revealed the increased Ca^2+^ responses. A coculture system of primary rat oligodendrocytes and neurons revealed that the mutant AR in oligodendrocytes affected the activity and synchronization of neurons. These findings suggest that dysregulated cell-to-cell communication plays a critical role in early SBMA pathology and that synaptic or ion channel–related proteins, such as contactin associated protein 2 (Cntnap2) and NALCN channel auxiliary factor 1 (Fam155a), are potential therapeutic targets for SBMA.

## Abstract 2

Pathways associated with cation channels and synaptic function in oligodendrocytes are dysregulated in spinal and bulbar muscular atrophy, with disrupted output from oligodendrocytes to neurons.

## Graphical abstract

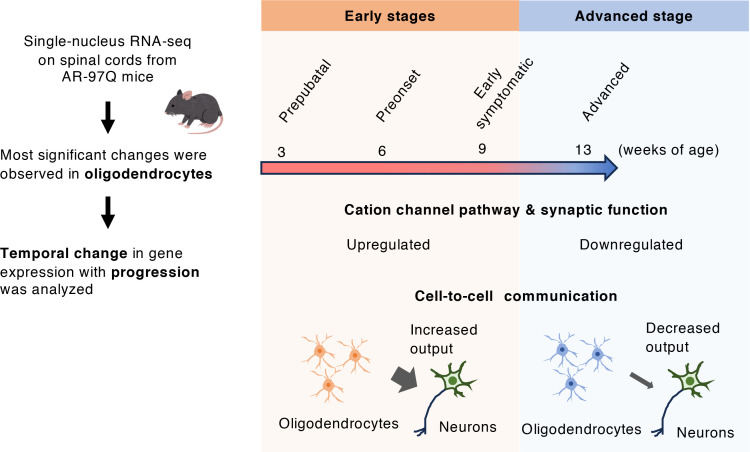

## Introduction

Spinal and bulbar muscular atrophy (SBMA) is an X-linked, adult-onset neuromuscular disease caused by a CAG repeat expansion within the first exon of the androgen receptor (*AR*) gene (1). It is characterized by progressive muscle weakness, atrophy, and fasciculation of the limb and bulbar muscles, which manifest between 30 and 60 years of age (2). Serum creatinine concentrations are significantly reduced in patients with SBMA and correlate with disease severity (3). MRI assessment of skeletal muscle and fat is another promising biomarker for tracking disease changes (4). Although the ligand-dependent toxicity of the polyglutamine-expanded AR protein is central to the pathogenesis of SBMA, the initiation and progression of its degenerative processes remain elusive.

As in other neurodegenerative disorders (5, 6), preclinical changes have been identified in SBMA. Most patients with SBMA notice hand tremors and muscle cramps more than 10 years before the emergence of limb weakness (7). Patients present with elevated serum creatine kinase levels and changes in muscle pathology before or at the onset of clinical symptoms (8). Female carriers of SBMA may develop mild muscular weakness associated with changes in neurogenic biomarkers, such as decreased motor unit number estimation and electromyographic abnormalities (9). Several mouse models of SBMA show reduced muscle force and altered contractile properties as early pathological events (10). The skeletal muscles of SBMA knockin mice show metabolic changes such as increased lipid metabolism and impaired glycolysis prior to denervation (11). Furthermore, presymptomatic SBMA transgenic mice (AR-100Q) show early changes in the expression pattern of genes involved in muscle contraction and structure (12).

In addition to the degeneration of motor neurons, skeletal muscles (13–17), and neuromuscular junctions (18), glial cell alterations are observed in certain mouse models of SBMA. The limb muscles of AR-113Q mice exhibit myopathy-like features and reduced mRNA levels of neurotrophin-4 and glial cell–derived neurotrophic factor, suggesting that glial cells are involved in the development of SBMA (15). TGF-β signaling, which plays a crucial role in the survival and function of adult neurons, was dysregulated in motor neurons as well as glial cells of SBMA model AR-97Q transgenic mice (19). It has also been reported that astrocyte proliferation is prominent and inflammatory M1 microglia are prevalent in the spinal cord of AR-97Q mice (13).

To understand the early pathogenesis of SBMA and to systematically assess the role of different cell types in the central nervous system of SBMA, we examined gene expression in the spinal cord of AR-97Q mice at the single-nucleus level during different stages of the disease. Oligodendrocytes had the highest number of differentially expressed genes (DEGs) from the preonset phase, and the expression of the genes related to ion channel and synapse function in oligodendrocytes was upregulated in the early stages but downregulated in the advanced stage of SBMA. A coculture system of primary rat oligodendrocytes and neurons showed that the mutant AR in oligodendrocytes affects the activity and synchronization of neurons, with increased levels of synaptic and ion channel–related proteins.

## Results

### Single-nucleus sequencing of the spinal cord from SBMA model AR-97Q mice reveals transcriptional alterations in oligodendrocytes.

**Figure 1:**
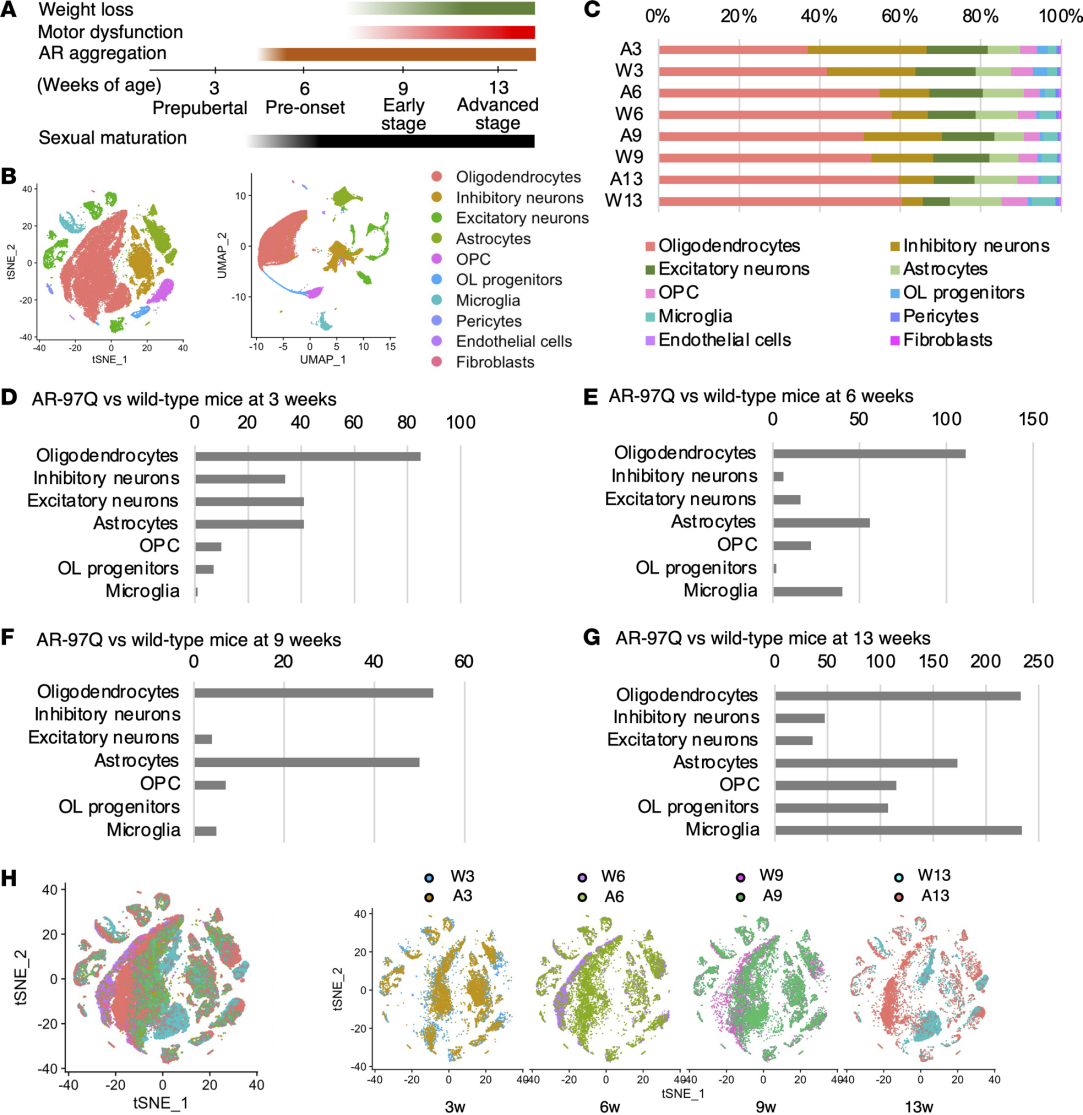
SnRNA-Seq of the spinal cords from AR-97Q and wild-type mice reveals cell type–specific differences. (**A**) A scheme showing the stages of disease in AR-97Q mice. (**B**) t-Distributed stochastic neighbor embedding (t-SNE) and uniform manifold approximation and projection (UMAP) plots of 54,456 nuclei from the spinal cords of all mice used in this experiment. OL, Oligodendrocytes; OPC, Oligodendrocyte precursor cell. (**C**) Proportion of the 9 cell types of each sample. (**D**–**G**) Number of DEGs in each cell type in AR-97Q mice and wild-type mice. Adjusted *P* < 0.05, absolute log_2_ fold-change (|log_2_FC|) ≥ 0.20 for 3 weeks of age (**D**) and |log_2_FC| ≥ 0.40 for 6, 9, and 13 weeks of age (**E**–**G**). (**H**) t-SNE plot of nuclei color-coded by each sample (left) and t-SNE plots of nuclei comparing AR-97Q mice with wild-type mice at 4 different disease stages (right). A, AR-97Q mice; W, wild-type mice (ex. A3 indicates AR-97Q mice at 3 weeks); *N* = 4 mice for each sample.

We conducted single-nucleus RNA sequencing (snRNA-Seq) on spinal cord samples from AR-97Q mice and wild-type mice at 4 stages: prepubertal (3 weeks of age), pre-onset (6 weeks of age), early symptomatic (9 weeks of age), and advanced (13 weeks of age) (*n* = 4 mice for each condition) (Figure 1A and Supplemental Figure 1, A and B; supplemental material available online with this article; https://doi.org/10.1172/jci.insight.182123DS1). After applying quality filters, 54,456 cells were retained for further analysis (Supplemental Figure 1C). Samples from all time points were combined, and data were projected onto 2 dimensions via t-distributed stochastic neighbor embedding (t-SNE) and uniform manifold approximation and projection (UMAP) (Figure 1B). Cell type designations were first determined by analyzing the DEGs in each cluster and manually comparing them with several canonical markers of each cell type (Supplemental Figure 2A). The oligodendrocyte progenitors, known as committed oligodendrocyte progenitors, differed from OPCs in that they lacked *Pdgfra* and *Cspg4* and expressed *Neu4* and genes expressed by undifferentiated oligodendrocytes, such as *Sox6*, *Bmp4*, and *Gpr17* (20) (Supplemental Figure 2B). The results revealed that the proportion of oligodendrocytes was the highest compared with that of each cell type within the samples, consistent with a previous report from human and mouse spinal cords (21, 22) (Figure 1C). The proportion of oligodendrocytes was lower in AR-97Q mice than in wild-type mice. Overall, the proportion of glial cells were increased with age, which is consistent with a previous report (23). The number of DEGs in the oligodendrocytes of AR-97Q mice compared with wild-type mice at 3, 6, and 9 weeks was the highest number of DEGs among all cell types (Figure 1, D–F). At 13 weeks, oligodendrocytes had the second highest number of DEGs after microglia (Figure 1G). Color-coding of the snRNA-Seq data by weeks of age also showed that the gene expression of oligodendrocytes in AR-97Q mice began to change from 3 weeks of age, with the differences from wild-type mice becoming more pronounced as time progressed (Figure 1H).

**Figure 2:**
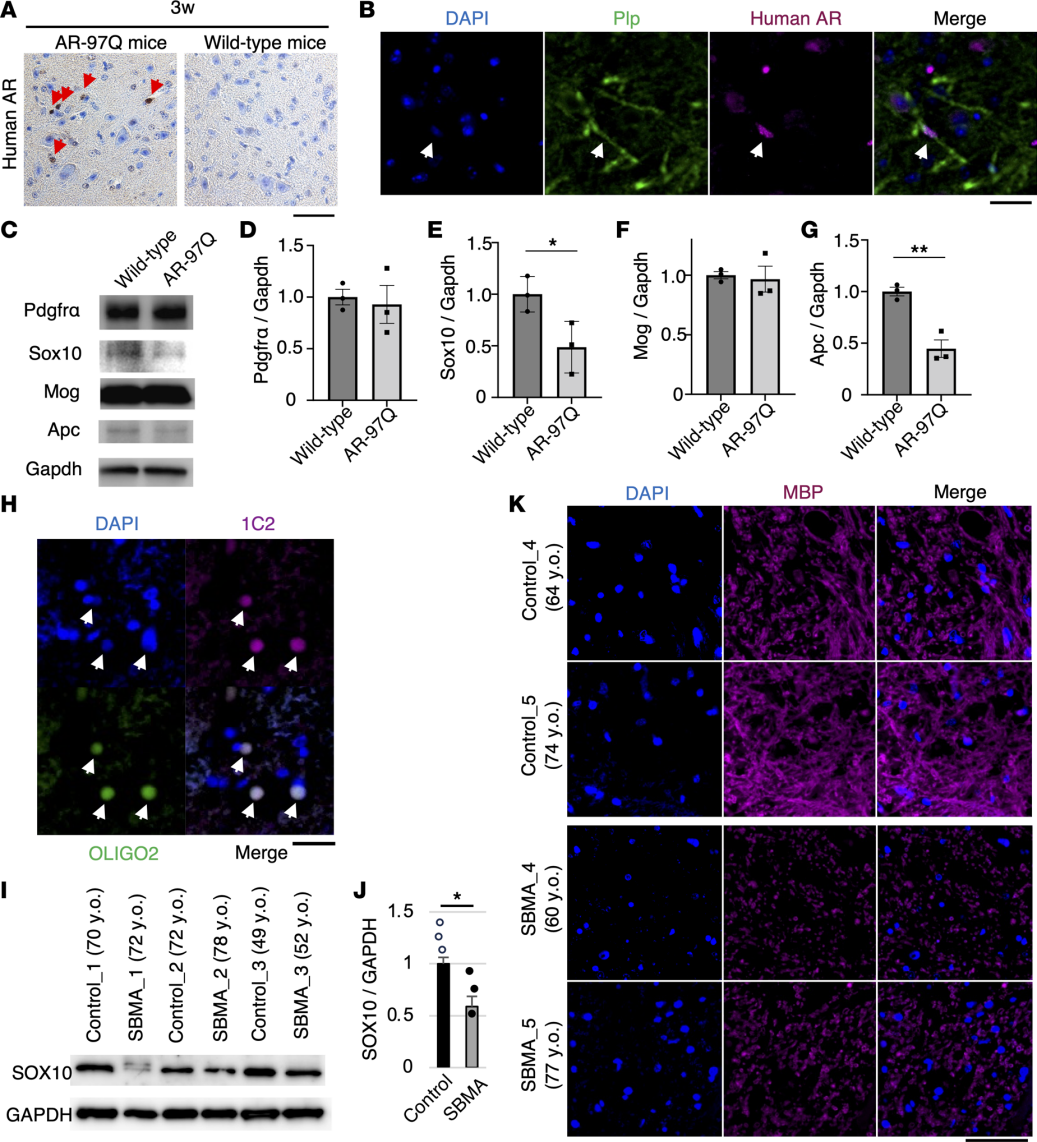
Oligodendrocytes are impaired in AR-97Q mice and patients with SBMA. (**A**) Immunostaining of human androgen receptor (*AR*) in the glial cells of AR-97Q mice at 3 weeks. The arrows indicate glial cells expressing human AR. (**B**) Immunofluorescence staining of human AR in the oligodendrocytes of AR-97Q mice at 3 weeks. The arrow indicates a cell of interest. (**C**) Immunoblotting of Pdgfrα, Sox10, Mog, and Apc in the spinal cord of AR-97Q mice at 13 weeks. (**D**–**G**) Quantitative immunoblot analysis of Pdgfrα (**D**), Sox10 (**E**), Mog (**F**), and Apc (**G**) in the spinal cord of AR-97Q mice at 13 weeks. (**H**) Immunofluorescence staining of polyglutamine in the oligodendrocytes of the spinal cord from patients with SBMA. The arrows indicate oligodendrocytes with 1C2-positive aggregation. (**I**) Immunoblotting of SOX10 in autopsied spinal cords in disease controls and SBMA participants. All participants were males. (**J**) Quantitative immunoblot analysis of SOX10 in the spinal cords of control and SBMA participants (*n* = 3 participants per group). (**K**) Immunohistochemical analysis of MBP in the autopsied spinal cords of control and SBMA participants. Error bars indicate the SEM. *P < 0.05 and **P < 0.01, unpaired 2-sided *t* test. Scale bars: 50 μm (**A** and **K**) or 25 μm (**B** and **H**). y.o., years old.

Immunostaining with a human-specific AR antibody demonstrated the presence of human AR in glial cells within the spinal cords of AR-97Q mice at 3 weeks of age, as well as in oligodendrocytes (Figure 2, A and B). However, no such staining was observed in the spinal cords of wild-type mice (Supplemental Figure 3). The AR-97Q mice express the full-length human *AR* comprising 97 CAGs under the control of a cytomegalovirus enhancer and a chicken β-actin promoter, resulting in the observation of human AR in other glial cells, including astrocytes (Supplemental Figure 4). Immunohistochemical staining with the 1C2 antibody, which specifically recognizes expanded polyglutamine, showed 1C2-positive cells in the spinal cords of AR-97Q mice at 6, 9, and 13 weeks. There were no 1C2-positive cells observed in AR-97Q mice at 3 weeks or in wild-type mice at any age (Supplemental Figure 5). Immunoblotting analyses revealed that the level of Sox10, a marker of OPCs and oligodendrocytes, and Apc, a marker upregulated in mature oligodendrocytes, were significantly lower in the spinal cord of AR-97Q mice than in those of wild-type mice at 13 weeks (Figure 2, C–G). In parallel, the number of Sox10- or Apc-positive oligodendrocytes in AR-97Q mice was found to be significantly reduced in comparison with wild-type mice at 13 weeks (Supplemental Figure 6). The immunoreactivity of myelin basic protein (Mbp), a marker of myelin, was lower in AR-97Q mice than in wild-type mice at 13 weeks (Supplemental Figure 7). The g-ratios, which are axon diameter per fiber diameter, were significantly elevated in the L5 ventral roots and L5 anterior horns of AR-97Q mice in comparison with wild-type mice at 13 weeks, suggesting that myelination was impaired in AR-97Q mice (Supplemental Figure 8). We further examined the implication of oligodendrocytes in human SBMA pathology. Polyglutamine inclusions were present in oligodendrocytes in autopsy specimens of the spinal cord from patients with SBMA (Figure 2H). Immunoblotting analyses showed that the level of SOX10 was significantly lower in the spinal cord of patients with SBMA than in control individuals (Figure 2, I and J). MBP in the autopsy spinal cord specimens of patients with SBMA had lower immunoreactivity than MBP in control patients (Figure 2K), suggesting that oligodendrocytes were impaired in the spinal cords of patients with SBMA.

### DEGs at each week.

**Figure 3:**
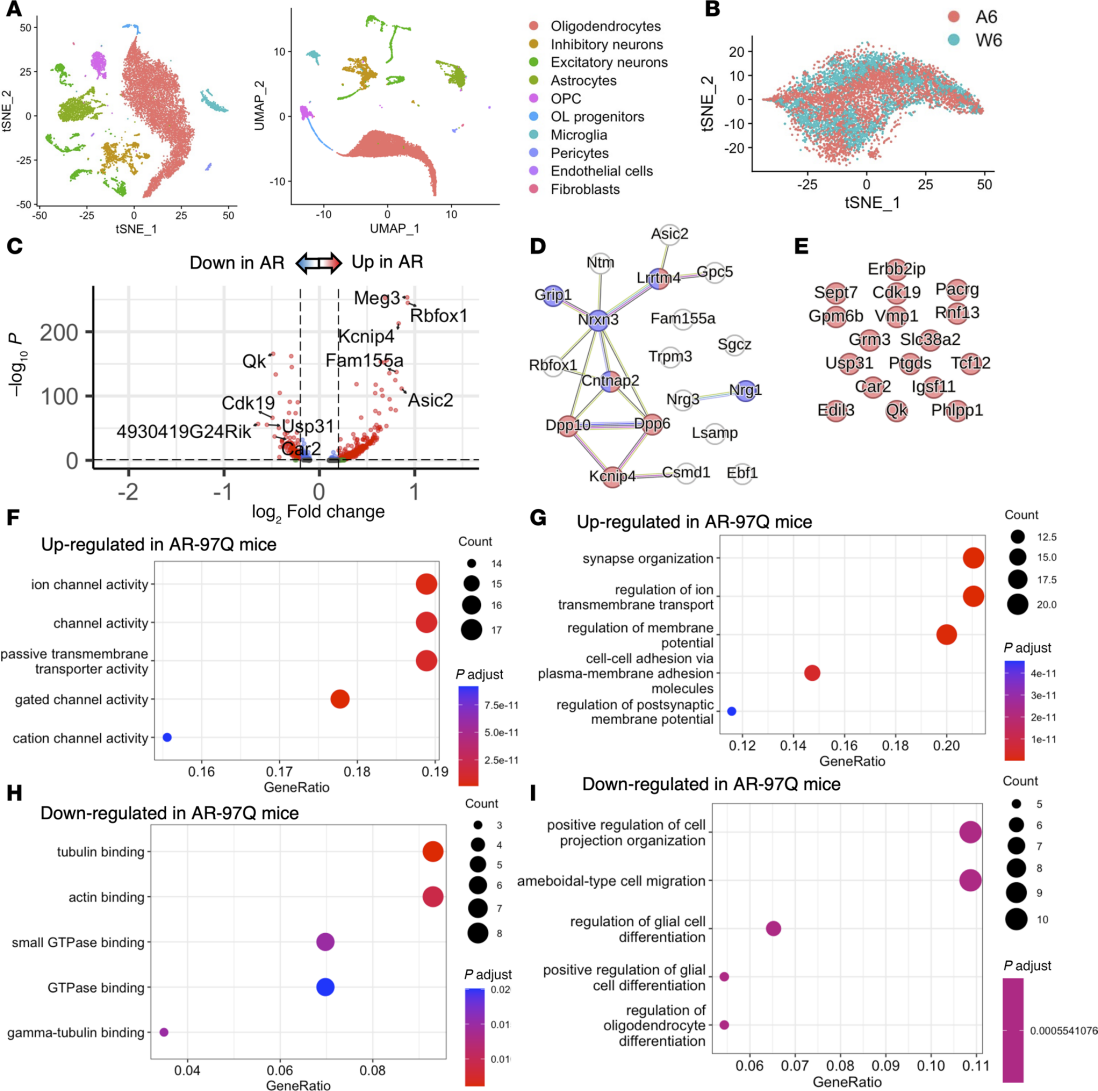
Genes associated with synaptic activity are upregulated at 6 weeks in AR-97Q mice. (**A**) t-Distributed stochastic neighbor embedding (t-SNE) and uniform manifold approximation and projection (UMAP) plots visualizing clusters of single nuclei in the spinal cord of AR-97Q and wild-type mice at 6 weeks. (**B**) Genotype-colored t-SNE plot of the oligodendrocyte cluster: orange dots represent AR-97Q mice (A6), and green dots represent wild-type mice (W6). (**C**) Volcano plot showing the DEGs of the oligodendrocyte cluster in AR-97Q and wild-type mice. The top 5 genes and last 5 genes are marked. (**D**) Protein-protein interaction (PPI) networks for the top 20 upregulated genes in AR-97Q mice. Genes colored in red have cation channel complex, and genes colored in purple have synaptic membrane in the cellular component of GO terms. Asic2, acid sensing ion channel subunit 2; Cntnap2, contactin associated protein 2; Fam155a, NALCN channel auxiliary factor 1. (**E**) PPI networks for the top 20 downregulated genes in AR-97Q mice. (**F** and **G**) The enrichment of the top 100 upregulated genes in AR-97Q mice in the biological process (**F**) and molecular function (**G**) categories (log_2_FC > 0.404). (**H** and **I**) The enrichment of the top 100 downregulated genes in AR-97Q mice in the biological process (**H**) and molecular function (**I**) categories (log_2_FC < –0.224). A6, AR-97Q mice at 6 weeks; W6, wild-type mice at 6 weeks. Line color code: sky blue, known interactions from curated databases; magenta, experimentally determined interactions; green, predicted from neighborhood analysis; red, predicted from gene fusions; blue, predicted from gene co-occurrence; pastel green, text mining; black, coexpression; and clear violet, protein homology.

To investigate the transcriptional changes in oligodendrocytes before the appearance of motor symptoms, we compared the data obtained from AR-97Q and wild-type mice at 6 weeks (Figure 3, A–C). The predicted protein interaction (PPI) networks for the top 20 DEGs upregulated in AR-97Q mice showed that they are related to each other and involve several genes associated with the cation channel complex and synaptic membrane, though such an interaction was unclear for downregulated genes (Figure 3, D and E). The Gene Ontology (GO) analysis of the top 100 upregulated DEGs in AR-97Q mice (log_2_FC > 0.404) revealed that the DEGs were associated with ion channel activity and synapse organization in the GO biological process and molecular function categories (Figure 3, F and G). In contrast, the top 100 downregulated genes in AR-97Q mice (log_2_FC < –0.224) were associated with tubulin binding, actin binding, and positive regulation of cell projection organization (Figure 3, H and I). To study whether the CAG repeat expansion led to alterations in the gene expression, we compared transcriptional changes of AR-24Q and AR-97Q mice at 6 weeks (Supplemental Figure 9, A–D). The PPI networks for the top 20 upregulated DEGs in AR-97Q mice showed that they are related to each other and involve several genes associated with the ion channel complex and synapse. In contrast, downregulated genes showed less clear interaction (Supplemental Figure 9, E and F). The GO analysis of the top 100 upregulated genes in AR-97Q mice (log_2_FC > 0.209) revealed associations with ion transmembrane transporter activity in the GO biological process and protein folding in the molecular function categories (Supplemental Figure 9, G and H). Conversely, the top 100 downregulated genes in AR-97Q mice (log_2_FC < –0.1905) were linked to acyltransferase activity and ensheathment of neurons (Supplemental Figure 9, I and J). These data suggest that the increase in ion channel– and synapse-related genes in AR-97Q mice before disease onset is due to CAG repeat expansion rather than AR overexpression.

We reanalyzed microarray data from the whole spinal cords of AR-97Q mice at 7 to 9 weeks reported in a previous study (24) (Supplemental Figure 10, A–C). A total of 25 genes were significantly upregulated in AR-97Q mice compared with AR-24Q mice (FDR < 0.1, FC > 1.5), and they were enriched in synapse assembly, suggesting that synaptic function is activated in the whole spinal cords of AR-97Q mice in the early stages of disease (Supplemental Figure 10D). The relative expression levels of genes related to cation channels and synaptic function were also increased in AR-97Q mice (Supplemental Figure 10, E and F).

To elucidate the transcriptional changes in oligodendrocytes after the onset of motor deficits, we compared the data obtained from AR-97Q and wild-type mice at 9 weeks (Supplemental Figure 11, A–C). The findings at 9 weeks were similar to those found at 6 weeks. The top 20 upregulated genes, but not downregulated genes, in AR-97Q mice included several genes associated with the cation channel complex and synapses (Supplemental Figure 11, D and E). The top 100 upregulated genes in AR-97Q mice (log_2_FC > 0.338) were associated with ion channel activity and synapse organization (Supplemental Figure 11, F and G). The top 100 downregulated genes in AR-97Q mice (log_2_FC < –0.234) were related to actin binding, gliogenesis, and ensheathment of neurons (Supplemental Figure 11, H and I). Collectively, these findings suggest that axon sheath formation is impaired by 9 weeks of age.

**Figure 4:**
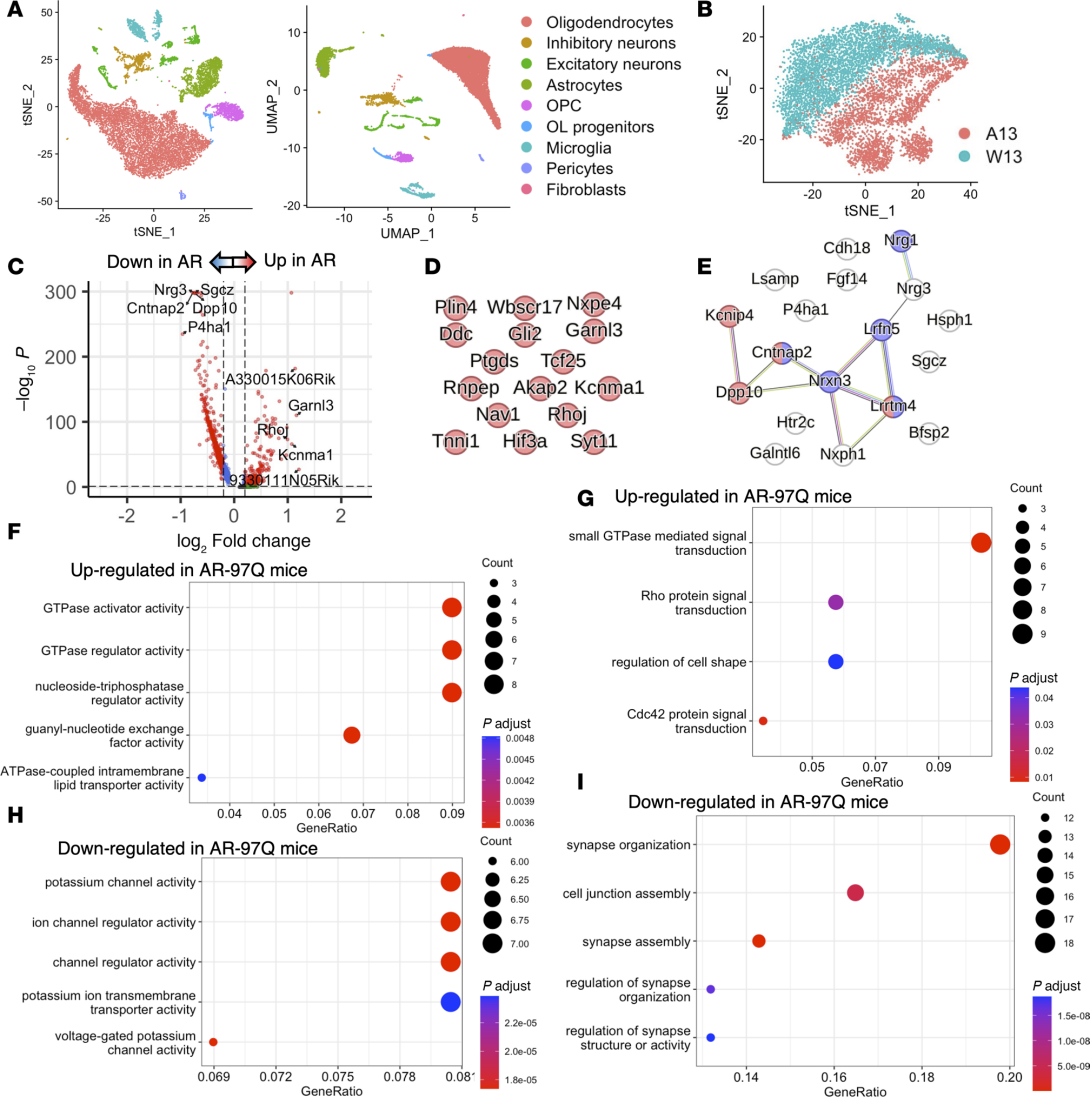
Genes associated with synaptic activity are downregulated at 13 weeks in AR-97Q mice. (**A**) t-Distributed stochastic neighbor embedding (t-SNE) and uniform manifold approximation and projection (UMAP) plots visualizing clusters of single nuclei in the spinal cord of AR-97Q and wild-type mice at 13 weeks. (**B**) Genotype-colored t-SNE plot of the oligodendrocyte cluster: orange dots represent AR-97Q mice (A13), and green dots represent wild-type mice (W13). (**C**) Volcano plot showing the DEGs in the oligodendrocyte cluster of AR-97Q and wild-type mice. The top 5 genes and last 5 genes are marked. (**D**) PPI networks for the top 20 upregulated genes in AR-97Q mice. (**E**) PPI networks for the top 20 downregulated genes in AR-97Q mice. Genes colored in red have cation channel complex in the cellular component of GO terms, and genes colored in purple have synaptic organization in the biological component of GO terms. (**F** and **G**) The enrichment of the top 100 upregulated genes in AR-97Q mice in the biological process (**F**) and molecular function (**G**) categories (log_2_FC > 0.415). (**H** and **I**) The enrichment of the top 100 downregulated genes in AR-97Q mice in the biological process (**H**) and molecular function (**I**) categories (log_2_FC < –0.436). A13, AR-97Q mice at 13 weeks; W13, wild-type mice at 13 weeks.

To investigate oligodendrocyte heterogeneity in the advanced stages of SBMA, data from 13-week-old AR-97Q mice and wild-type mice were compared (Figure 4, A–C). The top 20 downregulated genes showed interaction, but the top 20 upregulated genes in AR-97Q mice were predicted to be less connected to each other (Figure 4, D and E). The top 100 upregulated genes in AR-97Q mice (log_2_FC > 0.415) were associated with GTPase activator and regulator activity (Figure 4, F and G), though these genes were downregulated at 6 weeks (Figure 3H). The top 100 downregulated genes in AR-97Q mice (log_2_FC < –0.436) were related to ion channel activity and synapse organization (Figure 4, H and I), though these pathways were activated at 6–9 weeks (Figure 3, F and G, and Supplemental Figure 11, F and G). Together, our results demonstrated that ion channel activity and synapse organization were augmented in the early stages of disease but suppressed at the advanced disease stage.

### Time course of differential gene expression.

Three-week-old AR-97Q mice showed neither motor symptoms nor nuclear aggregation of the polyglutamine-expanded AR (Supplemental Figure 5), in agreement with the fact that prepubertal patients with SBMA have no subjective symptoms (25). However, the toxicity of soluble polyglutamine oligomers, which appears before the onset of symptoms, has been shown in previous in vivo experiments (26). We thus investigated the heterogeneity of oligodendrocytes at 3 weeks. Unsupervised clustering identified 8 major cell types, and the volcano plot of DEGs demonstrated fewer changes at 3 weeks of age compared with other ages (Supplemental Figure 12, A–C). A total of 17 upregulated genes in AR-97Q mice were predicted to be closely related to each other and involved several genes associated with the cation channel complex and synaptic membrane, but the interactions in downregulated genes were not clear (Supplemental Figure 12, D and E). The upregulated genes in AR-97Q mice (17 genes, log_2_FC > 0.22) were associated with ion channel activity and synapse organization (Supplemental Figure 12, F and G). The downregulated genes in AR-97Q mice (67 genes, log_2_FC < –0.2) were related to small GTPase binding and carboxylic or organic acid biosynthetic processes (Supplemental Figure 12, H and I). The cluster of oligodendrocytes at 3 weeks was reclustered into 6 subclusters for downstream analysis (Supplemental Figure 13, A and B). Based on the cell subpopulation proportional diagram, subcluster 0 was mainly enriched in AR-97Q mice and was associated with neurexins and neuroligins, protein-protein interaction at synapses, and the neuronal system, according to Reactome pathway analysis (Supplemental Figure 13, C and D). The transcriptional changes observed in AR-97Q mice at 6 and 9 weeks were also observed to a lesser extent at 3 weeks.

To understand the mechanisms of oligodendrocyte transcriptional changes in the absence of nuclear aggregation, we searched ChIP-Atlas (http://chip-atlas.org) for transcription factors that are associated with the top 10 and last 10 DEGs in oligodendrocytes at 3 weeks (Supplemental Figure 14). Several of these transcription factors have been reported to interact with AR, suggesting that these genes are involved in the transcriptional dysregulation of *AR* even before nuclear aggregation occurs.

**Figure 5:**
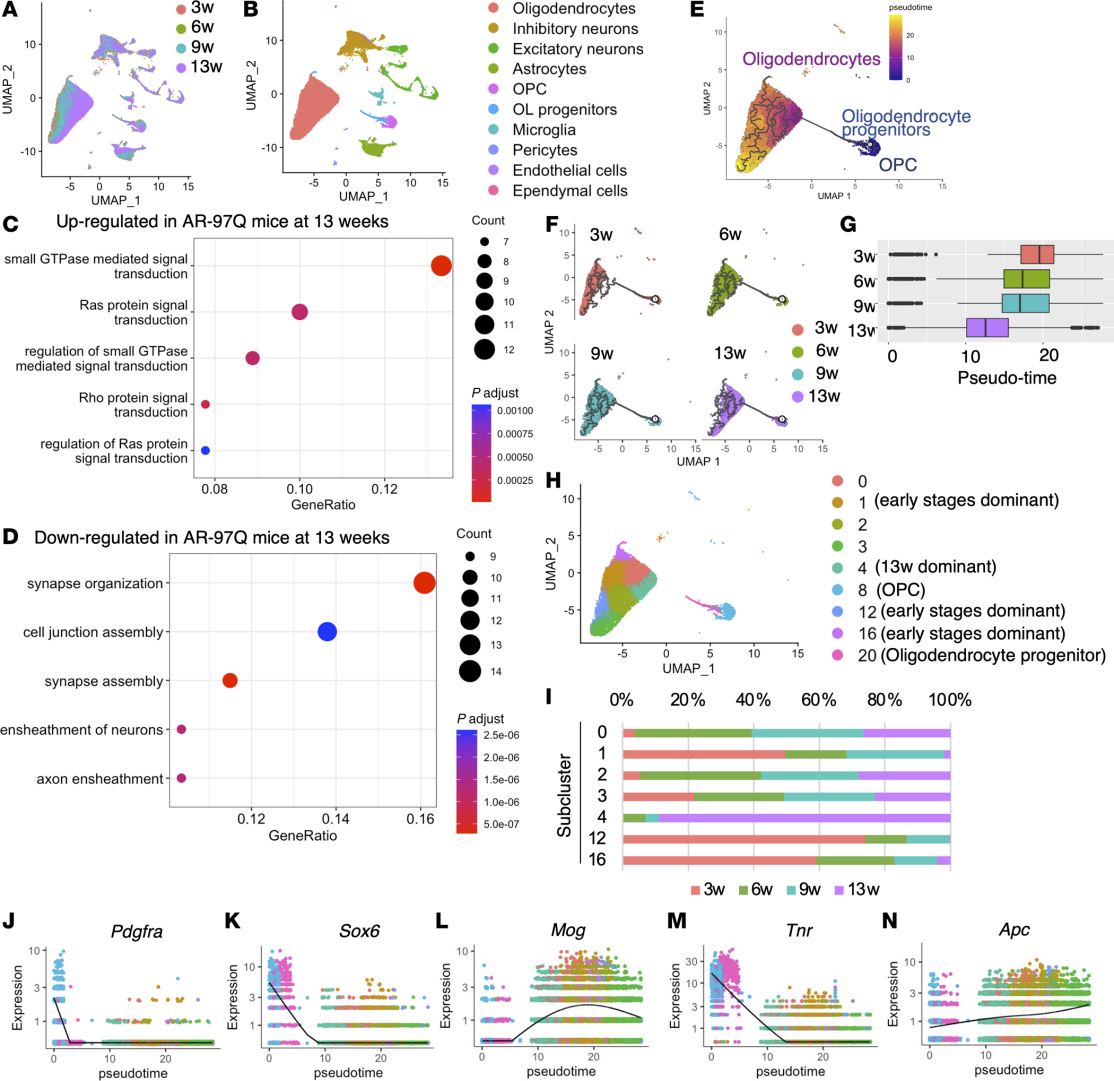
Transcriptional changes in oligodendrocytes according to disease stage. (**A**) Uniform manifold approximation and projection (UMAP) plots of all AR-97Q mice samples color-coded by each week of age (resolution = 1.2). (**B**) UMAP plots of each cell type of AR-97Q mice at 4 disease stages. (**C** and **D**) The enrichment of 100 genes upregulated (**C**) or downregulated (**D**) in AR-97Q mice at 13 weeks in the biological processes category (log_2_FC > 0.275 or log_2_FC < –0.1). (**E**) Pseudotime analysis inferred from the oligodendrocyte lineage cell clusters of AR-97Q mice in 4 disease stages. (**F**) UMAP visualization of oligodendrocyte lineage cell clusters colored by weeks of age. (**G**) Box plot showing the distribution of pseudotime within each sample. Vertical bars indicate median values. (**H**) UMAP visualization of oligodendrocyte lineage cell clusters colored by the Seurat package. (**I**) Proportion of each subcluster of each sample. (**J**–**N**) Pseudotime kinetics of *Pdgfra* (**J**), *Sox6* (**K**), *Mog* (**L**), *Tnr* (**M**), and *Apc* (**N**).

To investigate the timeline of transcriptional changes in SBMA, data from AR-97Q mice in 4 disease stages were compared (Figure 5, A and B). The top 100 upregulated genes in the oligodendrocytes of AR-97Q mice at 13 weeks (log_2_FC > 0.275) compared with those at 3, 6, and 9 weeks were associated with small GTPase-mediated signal transduction activators (Figure 5C). In contrast, the top 100 downregulated DEGs in AR-97Q mice at 13 weeks (log_2_FC < –0.100) were related to synapse organization and cell junction assembly (Figure 5D). These findings were consistent with the DEGs between the 2 groups at each week. We utilized pseudotime trajectory analysis to reveal the differentiation process of oligodendrocyte lineage cells (Figure 5, E and F). The box plot shows that cells at 13 weeks exhibited a different pseudotime pattern from those at 3, 6, and 9 weeks (Figure 5G). The pseudotime kinetics of *Pdgfrα* (a marker of OPCs), *Sox6* (a marker of OPCs and oligodendrocyte progenitors), and *Mog* (a marker of oligodendrocytes) revealed that cluster 8 represented OPCs, cluster 20 represented oligodendrocyte progenitors, and other clusters represented oligodendrocytes (Figure 5, H–L). Compared with other oligodendrocyte clusters, cluster 4, the predominant cluster at 13 weeks, exhibited high expression of *Tnr*, a marker of OPCs and oligodendrocyte progenitor cells, and low expression of *Apc*, a marker of mature oligodendrocytes, indicating that oligodendrocytes at 13 weeks are immature (Figure 5, M and N). In contrast, the oligodendrocyte lineage cells of wild-type mice showed almost no deviation in pseudotime distribution over time compared with those of AR-97Q mice (Supplemental Figure 15). Immunoblotting analyses revealed that the levels of Sox10 and Apc were significantly increased in the spinal cord of AR-97Q mice compared with those of wild-type mice at 6 weeks (Supplemental Figure 16, A–E). However, there was no significant difference in the number of Sox10- or Apc-positive oligodendrocytes between the 2 groups (Supplemental Figure 16, F–K), suggesting that the amount of protein in each cell increased in AR-97Q mice compared with wild-type mice at 6 weeks. The number of Sox10- and Apc-positive cells significantly decreased in AR-97Q mice at 13 weeks (Supplemental Figure 6). Similar results were obtained when AR-24Q mice were employed as a comparator (Supplemental Figure 17).

### Genes associated with ion channels and synaptic activity are upregulated in the early stages of SBMA.

**Figure 6:**
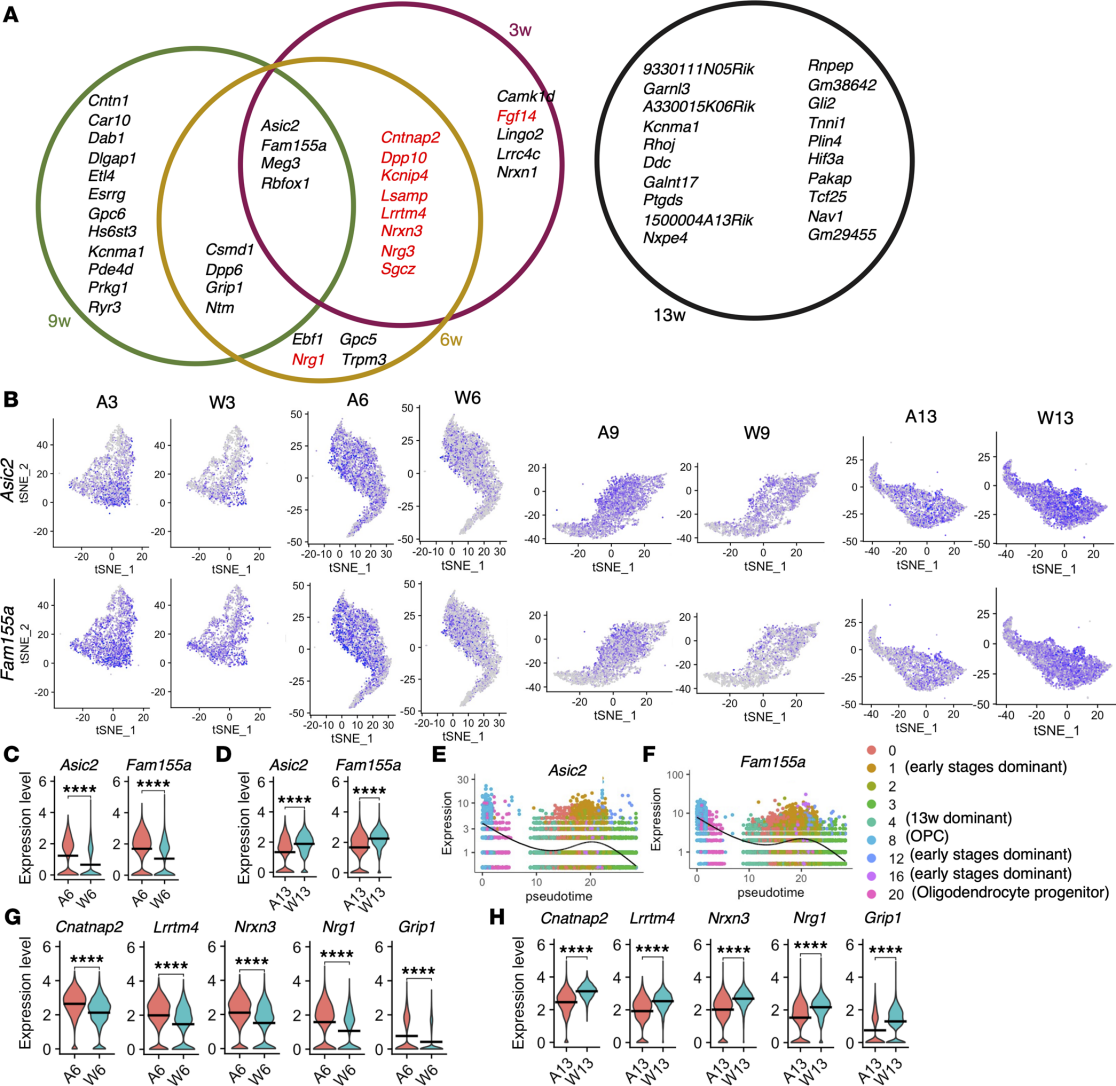
Top 20 DEGs in the oligodendrocytes of AR-97Q mice and wild-type mice in each week. (**A**) Common top 20 DEGs across the different weeks of age. Genes in red are the top 20 genes that are downregulated in AR-97Q mice at 13 weeks. (**B**) Feature plots of *Asic2* and *Fam155a* expression in the oligodendrocyte cluster of each sample. (**C** and **D**) Violin plots of *Asic2* and *Fam155a* expression in the oligodendrocytes of AR-97Q and wild-type mice at 6 weeks (**C**) and 13 weeks (**D**). (**E** and **F**) Pseudotime kinetics of *Asic2* (**E**) and *Fam155a* (**F**) in the oligodendrocyte lineage cells of AR-97Q mice at 3, 6, 9, and 13 weeks. UMAP visualization of oligodendrocyte lineage cell clusters in AR-97Q mice is shown in Figure 5G. (**G** and **H**) Violin plots of *Cntnap2*, *Lrrtm4*, *Nrxn3*, *Nrg1*, and *Grip1* expression in the oligodendrocytes of AR-97Q mice and wild-type mice at 6 weeks (**G**) and 13 weeks (**H**). Vertical bars indicate mean values (**C**, **D**, **G**, and **H**). ****P < 0.0001. Unpaired 2-tailed *t* test.

The top 20 upregulated genes in AR-97Q mice at each week were compared to elucidate the broad range of changes that are common in the early disease stages (Figure 6A). *Asic2*, *Fam155a*, *Meg3*, and *Rbfox3* were among the top 20 shared DEGs at 3, 6, and 9 weeks of age. Among them, the expression levels of *Asic2* and *Fam155a*, which are associated with cation channels, specifically sodium channels, in the oligodendrocytes of AR-97Q mice were increased at 6 weeks and decreased at 13 weeks compared with those in the oligodendrocytes of wild-type mice (Figure 6, B–D). Quantitative real-time polymerase chain reaction (RT-PCR) analysis showed that *Asic2* and *Fam155a* mRNA levels were significantly increased in the spinal cords of AR-97Q mice at 6 weeks and significantly suppressed in those of AR-97Q mice at 13 weeks compared with wild-type mice (Supplemental Figure 18, A–D). Immunoblot analysis revealed that Asic2 and Fam155a protein levels were significantly increased in the spinal cords of AR-97Q mice at 6 weeks and significantly suppressed in those of AR-97Q mice at 13 weeks (Supplemental Figure 18, E–J). Comparison of Asic2 and Fam155a protein levels between AR-24Q and AR-97Q mice at 6 weeks also revealed their increased levels in AR-97Q mice (Supplemental Figure 18, K–M). The pseudotime analysis of *Asic2* and *Fam155a* showed that their expression levels were elevated in clusters 1, 12, and 16, early stage–dominant clusters, and decreased in cluster 4, a late stage–dominant cluster (Figure 5I and Figure 6, E and F). The analysis of oligodendrocyte lineage cells of wild-type mice showed almost no deviation in the expression levels of *Asic2* and *Fam155a* among the 4 stages (Supplemental Figures 15 and 19). Immunofluorescence analysis of AR-97Q mouse spinal cords verified the increased levels of Asic2 and Fam155a in oligodendrocytes at 6 weeks and their decreased levels in oligodendrocytes at 13 weeks compared with those in the oligodendrocytes of wild-type mice (Supplemental Figures 20 and 21).

The top 20 upregulated genes in the oligodendrocytes of AR-97Q mice at 3 and/or 6 weeks included 10 of the top 20 genes that were downregulated in the oligodendrocytes of AR-97Q mice at 13 weeks (Figure 6, A, G, and H). Immunoblotting analysis of autopsied spinal cords from patients with SBMA also revealed reduced levels of ASIC2 and FAM155A compared with controls (Supplemental Figure 22).

To further clarify the role of oligodendrocytes in SBMA, high-dimensional weighted gene coexpression network analysis (hdWGCNA) (27) was performed using oligodendrocyte data from wild-type and AR-97Q mice at 6 and 13 weeks (Supplemental Figure 23A), and 5 distinct modules were identified from the oligodendrocyte cluster (Supplemental Figure 23B). The M1 module, which is associated with synaptic transmission and was the most altered in the comparison between AR-97Q and wild-type mice, was significantly upregulated in AR-97Q mice compared with wild-type mice at 6 weeks and was significantly suppressed in AR-97Q mice compared with wild-type mice at 13 weeks (Supplemental Figure 23, C–E). These findings were consistent with the results observed in the comparison of snRNA-Seq data from oligodendrocytes of AR-97Q and wild-type mice at 6 and 13 weeks.

### Transcriptional dysregulation in other cell types.

To further understand the dysregulation of other cell types in SBMA, the upregulated genes in other cell types were compared in AR-97Q mice (Supplemental Figure 24). *Camk1d* was commonly upregulated in the oligodendrocytes, astrocytes, inhibitory neurons, and excitatory neurons clusters at 3 weeks. At 6 weeks, 6 genes were universally upregulated in oligodendrocytes, astrocytes, and microglia clusters in AR-97Q mice, and 3 of them, *Asic2*, *Meg3*, and *Rbfox1*, were among the top 20 upregulated genes in oligodendrocytes throughout 3, 6, and 9 weeks of age.

The upregulated genes in the OPCs of AR-97Q mice at 6 weeks (18 genes, log_2_FC < 0.37) were associated with synapse assembly and cell junction assembly. At 13 weeks, the top 100 downregulated genes in the OPCs of AR-97Q mice (log_2_FC < –0.368) were associated with ion transmembrane transport and cell junction assembly (Supplemental Figure 25). Changes in the OPCs of AR-97Q mice were similar to those in the oligodendrocytes of AR-97Q mice, indicating that the alterations in oligodendrocytes are similar across oligodendrocyte lineage cells. The hdWGCNA using OPC data from wild-type and AR-97Q mice at 6 and 13 weeks identified 10 modules (Supplemental Figure 26A) and revealed that the M5 module, which was associated with synaptic transmission, was significantly elevated in AR-97Q mice at 6 weeks and was significantly suppressed in AR-97Q mice at 13 weeks (Supplemental Figure 26, B–D). These findings in OPCs were in agreement with the results observed when the comparison of snRNA-Seq data from OPCs of AR-97Q and wild-type mice at 6 and 13 weeks was performed (Supplemental Figure 25).

With respect to neuronal changes in AR-97Q mice, the number of DEGs was low in comparison with oligodendrocytes at all ages. Some genes associated with synaptic and ion transport were upregulated in AR-97Q mice (Mapt, Lrrtm4, Grip1, Nkain2, etc.), while some of the genes that were downregulated are also related to synaptic and ion transport at 6 and 9 weeks (Supplemental Figure 27).

### The SBMA oligodendrocyte cell model reflects the early pathogenesis of SBMA.

**Figure 7:**
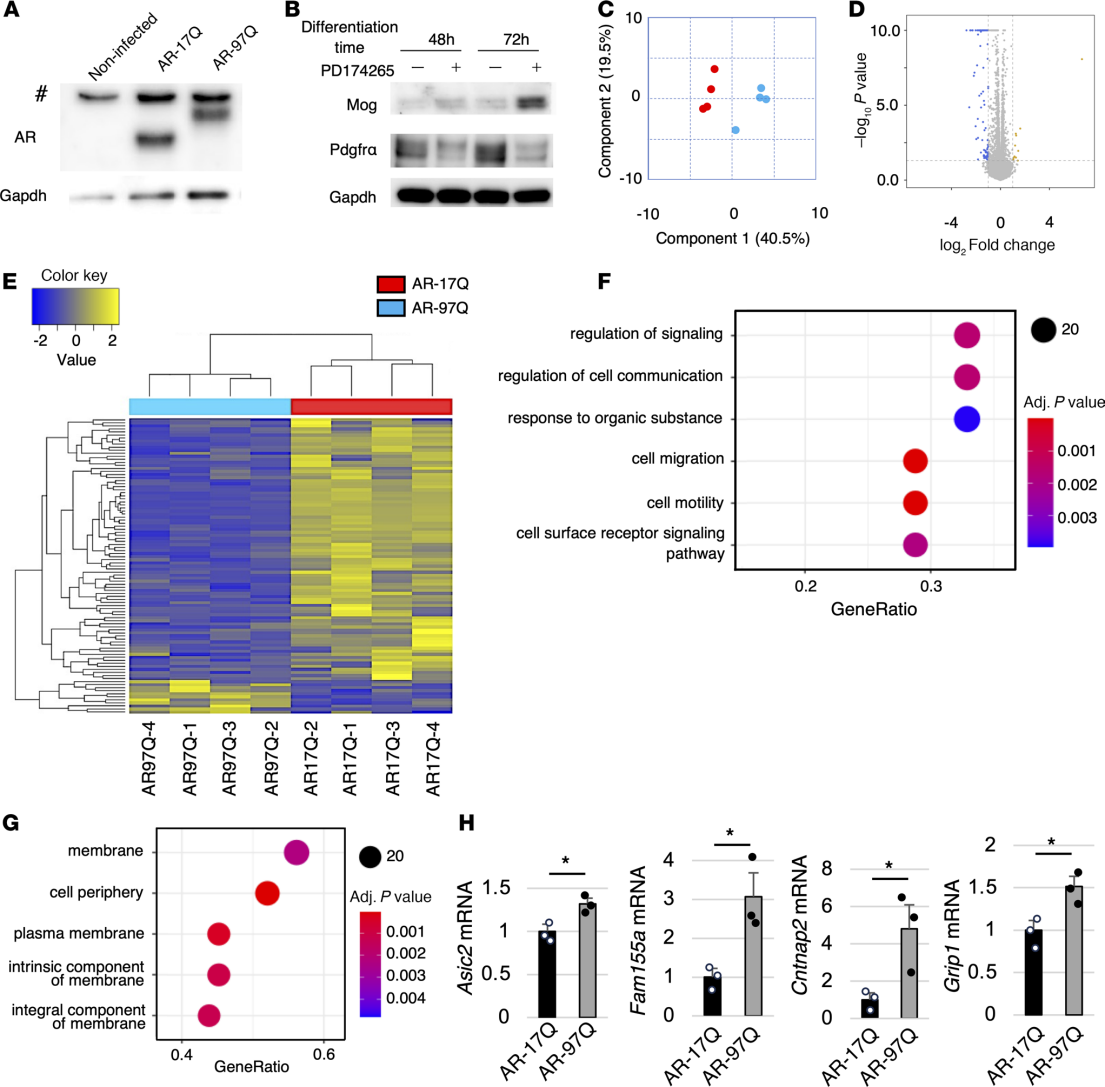
Oligodendrocyte cell model reflects the early pathogenesis of SBMA. (**A**) Immunoblotting of human AR in noninfected, AR-17Q, and AR-97Q cells. (**B**) Immunoblotting of Mog and Pdgfrα in AR-97Q cells with or without PD174265. (**C**) Multidimensional scaling analysis of the samples. Blue dots indicate AR-97Q cell samples, and red dots indicate AR-17Q cell samples. (**D**) Volcano plot of the 2 groups. (**E**) Heatmap showing the results of hierarchical clustering analysis. (**F** and **G**) GO terms related to the biological process (**F**) and cellular component (**G**) that are enriched in the DEGs identified by RNA-Seq. (**H**) The mRNA levels of *Asic2*, *Fam155a*, *Cntnap2*, and *Grip1* in AR-17Q cells and AR-97Q cells. AR-17Q and AR-97Q cells were treated with DHT. *N* = 3 samples for each group. Error bars indicate the SEM. *P < 0.05, unpaired 2-sided *t* test. #, nonspecific bands.

To further validate the transcriptional alterations identified in snRNA-Seq analysis, we generated an oligodendroglial cell model of SBMA using Oli-neu mouse oligodendroglial precursor cell line and treated them with dihydrotestosterone (DHT) (28) (Figure 7, A and B). Oli-neu cells were cultured in Sato medium and differentiated with 1 μM PD174265 on day 2. DHT at 10 nM was added on day 3, and total RNA was extracted from the cells on day 5. Eleven genes were significantly upregulated (>2-fold), and 86 genes (less than one-half) were significantly downregulated in AR-97Q cells (*P* < 0.05). Hierarchical clustering analysis revealed a clear difference between AR-17Q and AR-97Q cells (Figure 7, C–E). The DEGs were associated with the regulation of signaling and cell communication, and the synaptic membrane was the most enriched GO cellular component term (Figure 7, F and G), indicating that the DEGs may function in synapse or signal transduction and that the oligodendrocyte cell model of SBMA showed phenotypes similar to those of AR-97Q mice. To compare the oligodendrocytes in the SBMA cell model with those in AR-97Q mice, genes that were upregulated or downregulated in both the cell and mouse models of SBMA were selected (|log_2_FC| > 0.1, adjusted *P* < 0.05). The ratio of the number of these genes to the number of genes for which the expression levels were commonly measured by both RNA-Seq methods was calculated. The results revealed that the ratio was significantly lower at 13 weeks than at other weeks, suggesting that the oligodendrocytes in the SBMA cell model were more similar to those in AR-97Q mice in early disease stages than in advanced stages (Supplemental Figure 28A). The top 6 enriched GO terms of the genes that were upregulated in the oligodendrocytes of both the cell model and AR-97Q mice (log_2_FC > 0.1) at 6 weeks involved postsynapse organization and action potential, indicating that genes related to synapses are upregulated in both the SBMA cell and animal model oligodendrocytes (Supplemental Figure 28B). Quantitative RT-PCR analyses showed that the mRNA levels of *Asic2*, *Fam155a*, *Cntnap2*, and *Grip1* in AR-97Q cells were elevated, indicating that the genes related to ion channels and synapse function were also increased in the oligodendrocytes of the SBMA cell model as well as in the oligodendrocytes of AR-97Q mice in early disease stages (Figure 6, C and G, and Figure 7H).

### Interaction strength between oligodendrocytes and excitatory neurons is elevated in the early stages of SBMA.

**Figure 8:**
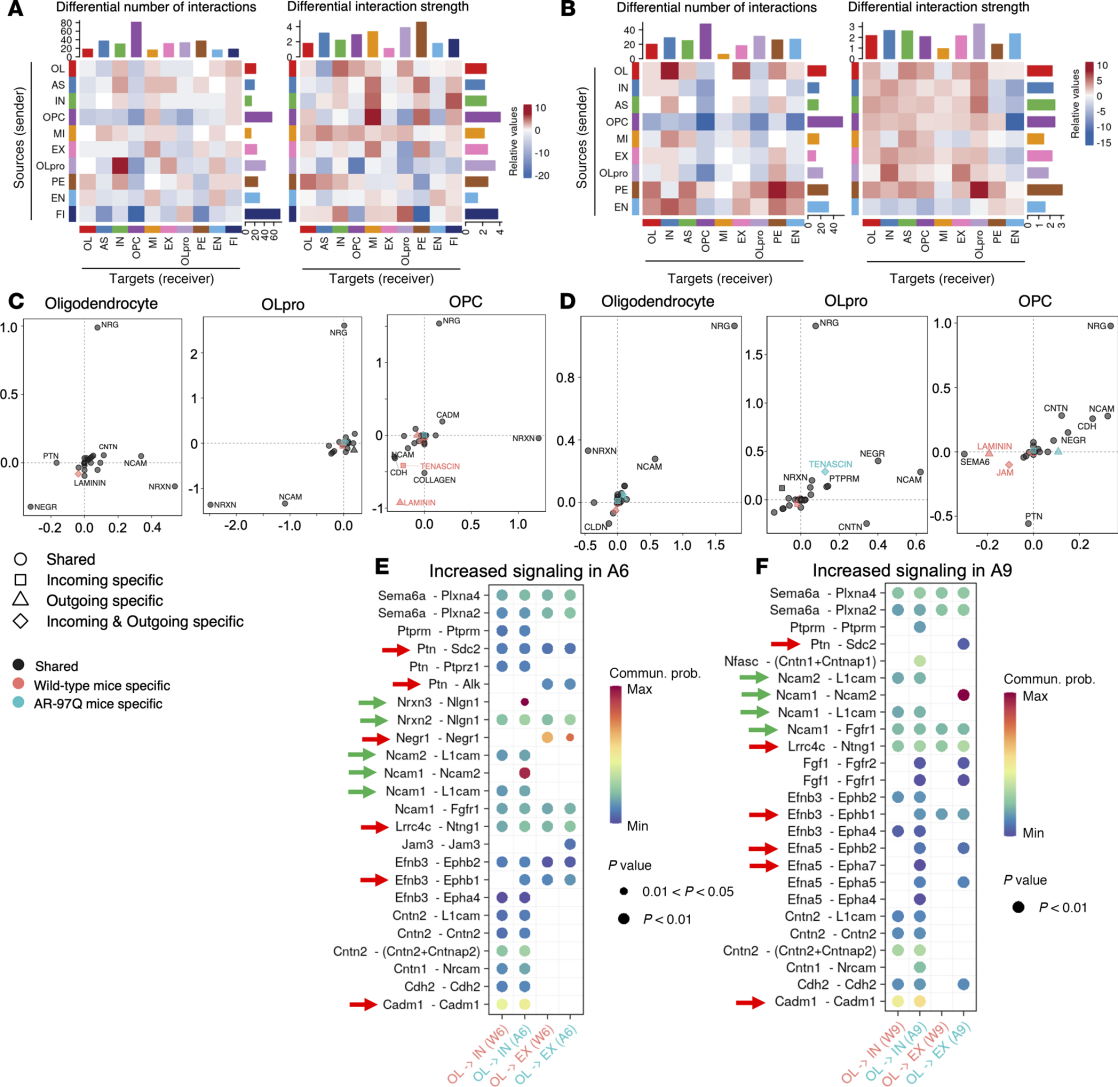
Interaction strength between oligodendrocytes and neurons is elevated in the early stages of SBMA. (**A** and **B**) Heatmap of differential interaction strength in AR-97Q mice compared with wild-type mice at 6 weeks (**A**) and 9 weeks (**B**). The top colored bar plot represents the sum of the values in columns displayed in the heatmap (incoming signaling). The right colored bar plot represents the sum of the values in rows (outgoing signaling). In the heatmap, red (or blue) represents increased (or decreased) signaling in AR-97Q mice compared with wild-type mice. Relative value = the interaction strength from source to target in AR-97Q mice – the interaction strength from source to target in wild-type mice. (**C** and **D**) Signaling changes in oligodendrocytes, oligodendrocyte progenitors (OLpro), and oligodendrocyte precursor cells (OPCs) in AR-97Q mice compared with wild-type mice at 6 weeks (**C**) and 9 weeks (**D**). The vertical axis represents the differential incoming interaction strength, while the horizontal axis represents the differential outgoing interaction strength. (**E** and **F**) Bubble plots of the communication probability of all the significant ligand-receptor interactions between oligodendrocytes and inhibitory neurons or excitatory neurons, which are increased in AR-97Q mice at 6 weeks (**E**) and 9 weeks (**F**). The dot color and size represent the communication probability and *P* values, respectively. The *P* values were computed from a 1-sided permutation test. The ligand-receptor pair interactions that are increased in AR-97Q mice at 6 and 9 weeks are indicated by red arrows. The ligand-receptor pairs related to NCAM and NRXN are indicated by green arrows. OL, oligodendrocytes; AS, astrocytes; IN, inhibitory neurons; MI, microglia; EX, excitatory neurons; OLpro, oligodendrocyte progenitors; PE, pericytes; EN, endothelial cells; FI, fibroblasts.

Then, we evaluated cell-cell communication patterns in the spinal cords of AR-97Q and wild-type mice by applying CellChat to the scRNA-Seq dataset. CellChat database takes into account multimeric ligand-receptor complexes and the effects of soluble and membrane-bound stimulatory and inhibitory cofactors. Using this database, CellChat can quantitatively infer and analyze intercellular communication networks from snRNA-Seq data. Using network analysis and pattern recognition approaches, CellChat predicts key signaling inputs and outputs for cells and how these cells and signals coordinate for function (29). A heatmap of the differential number of interactions and differential interaction strength in AR-97Q mice compared with wild-type mice showed that the number of interactions and interaction strength between cells were elevated overall at 6 and 9 weeks (Figure 8, A and B) and suppressed at 13 weeks (Supplemental Figure 29A). Analysis of oligodendrocyte signaling in AR-97Q mice compared with wild-type mice revealed that either the incoming or outgoing interaction strength of the neuregulin (NRG), neurexin (NRXN), and neural cell adhesion molecule (NCAM) pathways, which are associated with neuronal function, was increased in AR-97Q mice at 6 and 9 weeks (Figure 8, C and D). At 13 weeks, the incoming and outgoing interaction strength of the NRXN pathway was elevated in the oligodendrocytes of AR-97Q mice (Supplemental Figure 29B). Signaling changes in the oligodendrocyte progenitors and OPCs of AR-97Q mice compared with wild-type mice showed similar trends to the signaling changes in oligodendrocytes. To clarify the effects of oligodendrocytes on inhibitory neurons and excitatory neurons, the communication probability of the significant ligand-receptor pair interactions between oligodendrocytes and inhibitory neurons or excitatory neurons was calculated. NCAM and NRXN signaling was highly elevated between the oligodendrocytes and inhibitory neurons or excitatory neurons at 6 and 9 weeks (Figure 8, E and F), and it was decreased at 13 weeks (Supplemental Figure 29C). Ptn, Negr1, Lrrc4c, Efnb3, and Cadm1 signaling was also increased between oligodendrocytes and neurons at 6 and 9 weeks, and it was decreased at 13 weeks. Many of these factors are associated with cell adhesion and synapse formation.

### The mutant AR in oligodendrocytes affects the activity and synchronization of neurons.

**Figure 9:**
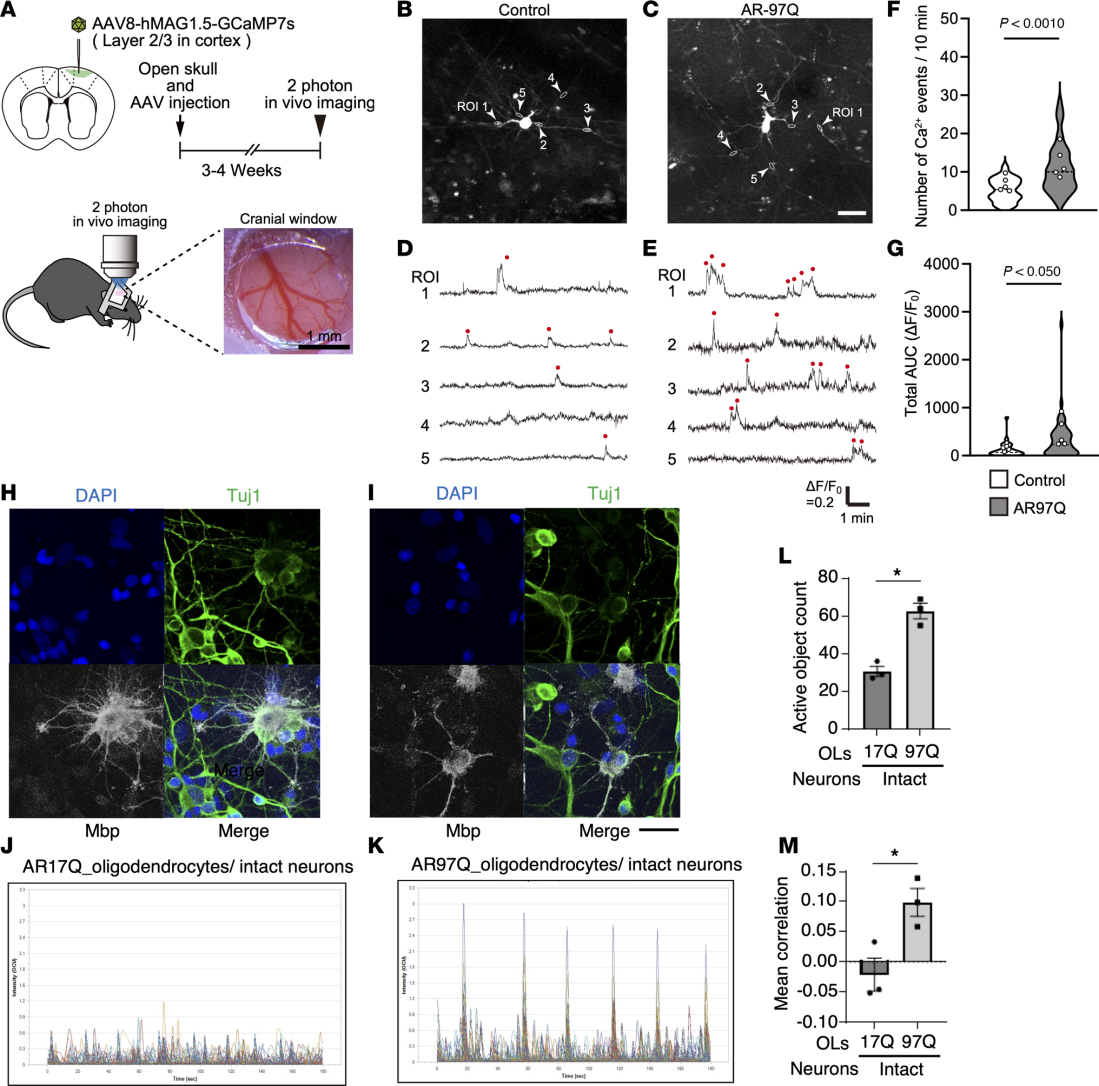
Oligodendrocytes in AR-97Q mice have increased Ca^2+^ activities, and mutant AR in oligodendrocytes affects the activity and synchronization of neurons. (**A**) Experimental protocol of calcium imaging in oligodendrocytes. (**B** and **C**) Representative images of GCaMP7s-positive oligodendrocytes of wild-type (**B**) and AR-97Q (**C**) mice observed in 2-photon in vivo imaging. ROI, region of interest. (**D** and **E**) Ca^2+^ traces detected from oligodendrocyte processes in the cerebral cortex of wild-type (**D**) and AR-97Q (**E**) mice. Red rods indicate the point of Ca^2+^ transient. (**F** and **G**) The number of Ca^2+^ events (**F**) and total AUC (**G**) on a process were significantly higher in the oligodendrocytes from AR-97Q mice compared with wild-type mice at 9 weeks of age. Violin plots show median (black dashed line) and distribution of the data. Wild-type: *N* = 69 processes (20 cells from 5 mice); AR-97Q mice: *N* = 107 processes (24 cells from 5 mice), Mann-Whitney *U* test. Circles on the violin plots indicate individual means of each mouse (number of Ca^2+^ events: *P* = 0.0219; total AUC: *P* = 0.0381, respectively. Unpaired 2-tailed *t* test). (**H** and **I**) Immunofluorescence staining of Tuj1 and Mbp in AR17Q_oligodendrocytes/intact neurons (**H**) and AR97Q_oligodendrocytes/intact neurons coculture models (**I**). (**J** and **K**) Calcium imaging traces of the coculture systems of AR17Q_oligodendrocytes/intact neurons (**J**) and AR97Q_oligodendrocytes/intact neurons (**K**). The *y* axis shows the intensity of the signal in the range from 0 to 3.3. (**L** and **M**) Active object count (**L**), mean correlation (**M**) of the coculture systems of AR17Q_oligodendrocytes/ intact neurons and AR97Q_oligodendrocytes/intact neurons. Error bars indicate the SEM. *P < 0.05, unpaired 2-sided *t* test. Scale bars: 20 μm (**B** and **C**) or 50 μm (**H** and **I**). OLs, oligodendrocytes.

To evaluate the differences of cellular activities of oligodendrocytes in AR-97Q mice in vivo, an adeno-associated virus (AAV) vector encoding fluorescence calcium indicator (GCaMP7s) driven by the human myelin-associated glycoprotein promoter was injected into the cerebral cortex of AR-97Q and wild-type mice given the surgery of craniotomy. Three weeks after the AAV injection, 2-photon in vivo calcium imaging of oligodendrocytes was performed (Figure 9, A–C, and Supplemental Figure 30, A and B). The number of Ca^2+^ events and the total AUC were significantly higher in the oligodendrocyte processes from AR-97Q mice compared with wild-type mice at 9 weeks of age (Figure 9, D and E). The amplitude and latency of the single Ca^2+^ transients were not significantly altered between the oligodendrocytes from AR-97Q and wild-type mice (Supplemental Figure 30, C and D). These results revealed that the Ca^2+^ activities of oligodendrocytes were increased in AR-97Q mice compared with wild-type mice, which was consistent with the snRNA-Seq results indicating the increase in ion channel and synaptic function of oligodendrocytes at early stages of SBMA.

Electrophysiological studies have reported excitatory changes in axons in patients with SBMA (30). To examine the neural activity in cultured cells, primary rat neurons were infected with lentivirus expressing AR-17Q (AR17Q_neurons) or AR-97Q (AR97Q_neurons) and lentivirus expressing the genetically encoded calcium indicator NeuroBurst-Orange to monitor spontaneous neuronal activity over time by measuring calcium fluctuations (Supplemental Figure 31, A and B). The number of active objects, the mean correlation, and the burst rate were increased in AR97Q_neurons compared with AR17Q_neurons on day 7 of coculture, indicating hyperactivity and disrupted synchronization of AR97Q_neurons (Supplemental Figure 31, C–G). We then developed the cell coculture systems of primary rat oligodendrocytes expressing AR-17Q and neurons expressing AR-17Q (AR17Q_oligodendrocytes/AR17Q_neurons model) and primary rat oligodendrocytes expressing AR-97Q and neurons expressing AR-97Q (AR97Q_oligodendrocytes/AR97Q_neurons model), and the neuronal activity was measured on day 7 of coculture (Supplemental Figure 31, H and I). The mean correlation and the mean burst rate were increased in the AR97Q_oligodendrocytes/AR97Q_neurons model compared with the AR17Q_oligodendrocytes/AR17Q_neurons model (Supplemental Figure 31, J–N), in line with the observations in AR17Q_neurons and AR97Q_neurons (Supplemental Figure 30, D and E). We further generated the coculture systems of primary intact rat neurons cultured with AR17Q_oligodendrocytes (AR17Q_oligodendrocytes/intact neurons model) or AR97Q_oligodendrocytes (AR97Q_oligodendrocytes/intact neurons model) to investigate whether mutant AR in oligodendrocytes affects the neural network (Figure 9, F–I). Strikingly, the number of active objects and the mean correlation were significantly increased in the AR97Q_oligodendrocytes/intact neurons model compared with the AR17Q_oligodendrocytes/intact neurons model (Figure 9, J–M), indicating that the mutant AR in oligodendrocytes increases the neuronal activity and perturbs synchronization. Mutant AR in oligodendrocytes did not affect the mean burst rate of neurons, which was increased in the single culture of AR97Q_neurons (Supplemental Figure 32A). The mean intensity and the mean burst strength in the AR97Q_oligodendrocytes/intact neurons model was similar to those in the AR17Q_oligodendrocytes/intact neurons model (Supplemental Figure 32, B and C). Quantitative densitometry analysis of immunoblotting revealed that the levels of Olig2 and NeuN were similar between the AR17Q_oligodendrocytes/intact neurons and AR97Q_oligodendrocytes/intact neurons models (Supplemental Figure 32, D–F). However, the levels of Fam155a and Cntnap2, the proteins related to ion channels and synapse function, respectively, were significantly increased in the AR97Q_oligodendrocytes/intact neurons model compared with the AR17Q_oligodendrocytes/intact neurons model, as observed in the spinal cord of SBMA mice at their early disease stages (Supplemental Figure 32, G and H).

## Discussion

In the present study, we identified cell type– and disease stage–specific gene expression changes in the spinal cord of SBMA model mice using snRNA-Seq analysis. The transcriptional changes in oligodendrocytes were the most evident among all cell types; the number of DEGs in oligodendrocytes was the highest among all cell types in the early stages of disease. GO and pathway analyses of the DEGs in the oligodendrocyte clusters of SBMA model mice at each week showed that pathways associated with ion channels and synapses were activated in the oligodendrocytes of AR-97Q mice from the preonset to early symptomatic stage of disease, suggesting that cell hyperexcitation and the reinforcement of intercellular connections occur early in SBMA.

Oligodendrocytes mediate the rapid conduction of action potentials and provide trophic support for axonal and neuronal maintenance. The supply of energy metabolites from oligodendrocytes to axons is crucial in the neurological system. Disease-associated oligodendrocyte signatures have emerged as important contributors to the development of neurodegenerative diseases such as Huntington’s disease (31–33), Parkinson’s disease (34), and Alzheimer’s disease (AD) (35, 36); however, the role of oligodendrocytes in the pathogenesis of SBMA has yet to be elucidated. Oligodendrocytes and OPCs create functional unidirectional or bidirectional synaptic contacts and regulate synaptic plasticity (37–39). Myelin-forming oligodendrocytes detect electrical activity in axons and increase intracellular calcium levels (40), and alternatively, the depolarization of oligodendrocytes further increases the conduction velocity of myelin-forming axons (41). It was also demonstrated that oligodendrocyte depolarization increases the number of excitatory synaptic responses and facilitates the induction of long-term potentiation at synapses (42).

In the present study, genes related to ion channel activity were upregulated in the oligodendrocyte clusters of AR-97Q mice at 3, 6, and 9 weeks, suggesting that depolarization and repolarization are abnormally activated at the early stage of SBMA. Moreover, Asic2, an acid-sensing ion channel, is known to be localized at the synapse and promote long-term potentiation and synaptic plasticity. Recent studies on the pathophysiology of multiple sclerosis (MS) have indicated that the concentrations of calcium and sodium ions affect the degree of myelin damage. Analysis of human autopsied brain tissue showed increased *ASIC2* mRNA levels in MS samples compared with control participant samples (43). Neurons overexpressing ASIC2a fired more frequently than control neurons (44), suggesting the involvement of ASIC2 in the neuroexcitatory imbalances observed in epilepsy. Fam155a, known as NALCN channel auxiliary factor 1 (NALF1), is a voltage-gated ion channel responsible for the resting sodium permeability, which controls neuronal excitability. Fam155a is a member of the NALCN channelosome, and the ion channel complex has been reported to be associated with intellectual disability and developmental delay (45). Ion channels are critical components of cellular excitability, and cell hyperexcitation has been implicated in the pathogenesis of several neurodegenerative diseases. It has been suggested that hyperexcitability in amyotrophic lateral sclerosis (ALS) is driven by changes in voltage-gated sodium and potassium channels (46), as shown in mouse and cell models of ALS (47, 48). Studies on patients with ALS have shown that persistent sodium conductance is strongly associated with shorter survival, suggesting that alterations in cell excitability are pathogenic (46). Together, our results suggest that the sodium current is disrupted by oligodendrocytes, which is in line with the observed benefit of mexiletine, a sodium channel blocker, in patients with SBMA (49). Antiepileptic and antiarrhythmic drugs inhibit ion channels and may be potential therapeutics targeting the dysregulated pathways in oligodendrocytes of SBMA.

We also found that genes associated with synaptic function were upregulated in the early stages of SBMA, whereas they were downregulated in the advanced stage. These results were consistent with the results of the hdWGCNA. Disruption of Cntnap2 level leads to imbalanced excitatory and inhibitory neural networks, which are thought to be central to the pathophysiology of schizophrenia (50). Lrrtm4 is a postsynaptic protein that regulates the development and strength of glutamatergic synapses by interacting with presynaptic proteins such as Nrxns (51). Increased or decreased levels of Nrg1 result in the abnormal growth of dendritic spines and the disruption of excitatory and inhibitory synapses, suggesting that maintaining a balance of synaptic protein levels is critical to preserve synaptic function. Grip1 plays an essential role in synaptic plasticity and learning and memory (52). *Cntnap2*, *Dpp10*, *Kcnip4*, and *Dpp6* are involved in the potassium channel complex, and potassium channels are key regulators of synaptic plasticity (53). Ion channels at synapses contribute to proper synaptic function by regulating membrane potential and participating in neurotransmitter release and vesicle recycling. This close relationship between channels and synapses resulted in transcriptional changes in the present study, with both ion channel and synaptic genes being upregulated in early stages of the disease and downregulated in advanced stages. Collectively, our results indicate deficits in ion channel and synaptic functioning in oligodendrocytes in the early stages of SBMA. According to RNA-Seq or snRNA-Seq analyses of tissues from disease models or patients, oligodendrocytes in MS exhibited immune-related gene alterations (51), whereas oligodendrocytes in AD exhibited stress-related transcriptomic dysregulation associated with Erk signaling, tau pathology, and cognitive decline (54), suggesting pathological differences among neurodegenerative diseases.

The oligodendrocyte marker proteins were elevated in each oligodendrocyte at early stages of disease, and their expression was suppressed at the advanced stage. Given that Sox10 is a transcription factor required during the differentiation to promote myelin gene expression, the increase in Sox10 levels suggests that cellular signaling within oligodendrocytes and between oligodendrocytes and neurons are activated in AR-97Q mice at 6 weeks. While the number of oligodendrocytes in wild-type and that in AR-97Q mice were equivalent at 6 weeks, the expression of the genes related to synapse and ion channels were increased in each cell. These findings were supported by in vivo Ca^2+^ imaging showing an increased number of Ca^2+^ events in the oligodendrocytes of AR-97Q mice at their early stage of disease. In contrast, the number of oligodendrocytes as well as the expression of genes related to synapse and ion channels in each oligodendrocyte were decreased in AR-97Q mice compared with wild-type mice at the age of 13 weeks, indicating the consequence of progression of degenerative processes. The stage-specific changes in cell activities have been reported in other neurodegenerative diseases. Clinical studies using functional MRI have shown cortical and hippocampal hyperactivity in the early stages of AD, progressing to hypoactivity in the later stages of neurodegeneration (55–57). A previous study reported a greater number of hyperactive CA1 pyramidal neurons in the hippocampus of AD model mice than wild-type mice at an early disease stage; however, this number was reduced as the mice aged (58). Hyperexcitability occurs in the early stages of ALS, even prior to motor symptom onset, then progresses to hypoactivity in later stages (59). In an analysis using transmagnetic stimulation, patients in the early stages of ALS showed a hyperexcitable motor cortex, whereas patients in the later stages of ALS exhibited a hypoexcitable motor cortex compared with the control group (60). Transcriptional changes in the early stages of SBMA observed in this study may reflect the pathological mechanisms of SBMA, while transcriptional changes at 13 weeks may indicate consequential and/or compensatory changes after neurodegeneration.

Intercellular network analysis revealed changes in the interactions between oligodendrocytes and neurons and allowed us to infer a mechanism by which oligodendrocyte abnormalities drive neurodegeneration. Nrxn3 signaling between oligodendrocytes and inhibitory neurons was significantly elevated at 6 weeks, which is consistent with the snRNA-Seq results showing that *Nrxn3* was among the top 20 DEGs in the oligodendrocyte cluster of AR-97Q mice in the early stages of disease. Moreover, Ncam1/Ncam2 signaling between oligodendrocytes to inhibitory and excitatory neurons was inferred to be increased in the early disease stages. Elevated NCAM2 levels and altered submembrane Ca^2+^ dynamics are known to cause defects in synapse maturation, as implicated in the pathology of Down syndrome and other brain disorders associated with abnormal NCAM2 expression (61). The increased strength of NRG, NRXN, and NCAM signaling between oligodendrocytes and other cell types can lead to abnormalities in synaptic function as well as abnormalities in the function of the cell receiving the signal.

The in vivo calcium imaging of oligodendrocytes showed that the oligodendrocytes in AR-97Q mice had increased Ca^2+^ activities compared with those in wild-type mice, particularly with the number of Ca^2+^ events with undetectable changes in amplitude and its duration, indicating that oligodendrocyte processes in AR-97Q mice are hyperexcitable. The primary culture experiments indicated that neurons expressing AR97Q show hyperactivity and disrupted synchronization, both of which were reproduced by coculture of intact neurons with oligodendrocytes bearing AR97Q, suggesting that the mutant AR in oligodendrocytes affects the neuronal circuit activity. The levels of Fam155a and Cntnap2 were elevated in the AR97Q_oligodendrocytes/intact neurons model compared with the AR17Q_oligodendrocytes/intact neurons model as observed in AR-97Q mice at early stages and the Oli-neu oligodendrocyte cell model. Given that Cntnap2 is related to neurite branching and neuronal complexity (50), this protein likely plays a role in the hyperexcitability of neurons in SBMA.

Limitations of the present study are as follows. First, although we demonstrated oligodendrocyte alterations in autopsied patient tissues, early changes in patients with SBMA remain elusive. Development of biomarkers that can be measured in biosamples of patients and carriers of SBMA is needed. Second, we investigated the electrophysiological characteristics of oligodendrocytes through in vivo calcium imaging. However, additional patch-clamp experiments will ascertain the full extent of oligodendrocyte contributions to the pathogenesis of SBMA. Third, most of the insight gained in the present study is based on the findings of a transgenic mouse model overexpressing polyglutamine-expanded AR, although we adopted AR-24Q mice as a control group in key experiments. AR-97Q mice recapitulate key patient characteristics of SBMA, including testosterone-dependent disease progression and pronounced neuromuscular degeneration. In addition, RNA changes in the entire spinal cord prior to disease onset have been previously reported (24) and had the advantage of being used to validate snRNA-Seq analysis. However, the pathophysiology of AR-97Q mice differs from that of patients with SBMA in that AR is overexpressed and the disease progresses more rapidly. While the oligodendrocyte transcriptional changes identified in AR-97Q mice were partially confirmed by immunostaining and Western blotting of human tissue, further studies using a mouse model expressing pathogenic AR at endogenous levels and additional human samples are needed to clarify the pathogenesis of SBMA.

In conclusion, pathways associated with cation channels and synaptic function in oligodendrocytes are dysregulated in SBMA, resulting in disrupted output from oligodendrocytes to neurons. This pathway can be targeted by therapeutic interventions for SBMA.

## Methods

Further information can be found in Supplemental Methods.

### Sex as a biological variable.

Our study exclusively examined male mice because SBMA only affects males.

### Animals.

AR-97Q (Line #7–8) mice were bred and maintained in the animal laboratory of our institute (62). The mice were genotyped by PCR amplification using DNA extracted from the tail with the primers listed in Supplemental Table 1.

### Tissue processing for single-nucleus sequencing.

C57BL/6 (Japan SLC) and AR-97Q male mice were deeply anesthetized, the spinal column was cut at the hip level, and a PBS-filled syringe fitted with a 20 G needle (7.25 mm) was inserted into the caudal end of the spinal column to flush out the spinal cord. Spinal cords were snap-frozen in powdered CO_2_ in liquid nitrogen and stored at –80°C until processing. Spinal cords from 4 mice per group were pooled for homogenization and nuclear isolation. The nuclear isolation protocol was adapted from the Frankenstein protocol (https://www.protocols.io/view/frankenstein-protocol-for-nuclei-isolation-from-f-5jyl8nx98l2w/v3). Each sample was thawed and homogenized in a Dounce Homogenizer (Kimble Chase 2 mL Tissue Grinder) containing 500 μL freshly prepared ice-cold lysis buffer (10 mM Tris-HCl pH 7.4, 10 mM NaCl, 3 mM MgCl_2_, 0.1% Igepal, and 0.2 U/μL SUPERase-In RNase Inhibitor; AM2696, Thermo Fisher Scientific). Then, 900 μL of lysis buffer was added, and the samples were incubated for 5 minutes. The homogenate was filtered through a 70 μm cell strainer (08-771-2, Thermo Fisher Scientific) and centrifuged at 500*g* for 5 minutes at 4°C. The supernatant was removed, and the pellet was resuspended in 1.5 mL of lysis buffer and incubated for another 3 minutes on ice. Then, the nuclei were centrifuged at 500*g* for 5 minutes and resuspended in 1,500 μL of wash buffer (1× PBS with 1% BSA and 0.2 U/μL SUPERase-In RNase Inhibitor). The nuclei were washed and centrifuged at 500*g* for 5 minutes 3 times and filtered through a 40 μm cell strainer (08-771-1, Thermo Fisher Scientific). Then, Debris Removal Solution (130-109-398, Miltenyi Biotec Inc.) was added to the cell suspension to create a density gradient and remove dead cells and debris. The samples were resuspended in 3.1 mL of 1× PBS in a 15 mL tube, and 900 μL of Debris Removal Solution was added and mixed well. The solution was gently overlaid with 4 mL of cold 1× PBS, and the sample was centrifuged at 1,000*g* for 10 minutes at 4°C. The top 2 layers were aspirated and discarded. The bottom layer was left undisturbed, and the volume was increased to 15 mL with 1× PBS. Cells were gently mixed and centrifuged at 1,000*g* for 10 minutes at 4°C. The supernatant was removed, and the cells were resuspended in cold 0.04% BSA/PBS and stained with DAPI. Finally, nuclei labeled with DAPI were sorted with a FACSAria instrument (BD) with a 100 nm nozzle and 405 nm excitation laser. The instrument was controlled by a PC running FACSDiva software (BD). We collected at least 500,000 DAPI-positive nuclei from each condition. These nuclei were centrifuged at 500*g* for 10 minutes and inspected for visual appearance, and the concentration was adjusted to proceed to the next step.

### SnRNA-Seq.

A total of 10,000 nuclei per sample were run on the 10x Genomics Chromium Single Cell 3’ gene expression v3.1 platform. DAPI-positive nuclei were immediately loaded onto a Chromium Single Cell Processor (10x Genomics) to barcode the mRNA inside each nucleus. Sequencing libraries were constructed according to the manufacturer’s instructions, and the resulting cDNA samples were run on an Agilent Bioanalyzer using the High Sensitivity DNA Chip as a quality control to determine cDNA concentrations. The samples were run on an Illumina HiSeq X Ten with read 1 = 28 bp and read 2 = 90 bp to obtain ≥20,000 reads per cell. A total of 70,046 cells with 1.96 billion reads were sequenced for the 8 samples with an average of 8,756 cells per sample with 28,700 reads each. Reads were aligned and assigned to Ensembl mm10 transcript definitions using the Cell Ranger v5.0.1 pipeline (10x Genomics). The gene barcode matrices for each sample were imported into R using the Read10X function in the Seurat R package (v4.3.0) (63). The Seurat R package (v5.1.0) was used to process the data as for hdWGCNA.

### Quality control and filtering.

Based on the distribution of the number of genes detected in each cell and the distribution of the number of unique molecular identifiers (UMIs), nuclei with fewer than 200 genes or more than 3,000 genes were excluded from the downstream analyses. Nuclei with more than 1% of mitochondrial gene expression were excluded. Removal of outliers resulted in 54,456 total remaining cells for analysis. UMI counts were then normalized in Seurat 3.0, and the top 3,000 highly variable genes were identified using the FindVariableFeatures function with variance stabilization transformation.

### Dimension reduction and cluster annotation.

We performed a reference-based integration workflow with reciprocal principal component analysis. Clustering was performed using the Seurat functions FindNeighbors and FindClusters. Visualization of the integrated dataset was performed using t-SNE and UMAP, with the first 25 principal components at a resolution of 0.8 if not otherwise noted. For subclustering, oligodendrocyte clusters were taken as a subset from the integrated dataset of wild-type and AR-97Q mice at 3 weeks, and significant PCs were used for downstream clustering similar to above. The cell type designation was established by first analyzing the DEGs in each cluster and comparing them with several canonical markers for each cell type.

### Differential expression analysis and GO enrichment analysis.

Differential expression analysis between the 2 conditions was performed using FindMarkers. For the identification of DEGs, the log fold-change mentioned in each test and Wilcoxon’s signed rank test with Bonferroni’s correction for *P* value (<0.05) were used as cutoff values. GO enrichment analysis and Reactome pathway analysis were performed to determine the function of the DEGs using the R package clusterProfiler (64).

### Statistics.

GraphPad Prism 9.0 software was used for statistical analysis and the plotting of statistical graphs. Data are expressed as the mean ± SEM. Differences between the 2 groups were evaluated by a 2-tailed Student’s *t* test. The results were considered statistically significant at *P* < 0.05. Principal component analysis and heatmap generation, as well as enriched pathway analysis of microarray data, were performed using iDEP.94 (65). Upregulated DEGs in the oligodendrocytes of AR-97Q mice (log_2_FC > 0.1) at 6 weeks and the upregulated genes in AR-97Q cells compared with AR-24Q cells were analyzed with the Metascape portal (https://metascape.org/gp/index.html#/main/step1) (66).

### Study approval.

All animal experiments were performed in accordance with the National Institutes of Health *Guide for the Care and Use of Laboratory Animals* (National Academies Press, 2011) and with the approval of the Nagoya University Animal Experiment Committee. The collection of autopsied human tissues and their use in this study were approved by the Ethics Review Committee of Nagoya University Graduate School of Medicine, and written informed consent for the use of the specimens was obtained from the patients or patients’ next of kin. Experimental procedures involving human participants were conducted in accordance with the Declaration of Helsinki, the Ethical Guidelines for Medical and Biological Research Involving Human Subjects endorsed by the Japanese government.

### Data availability.

The data generated in this study are provided in the Supporting Data Values file. SnRNA-Seq data in this paper are available in the National Center for Biotechnology Information (NCBI) Gene Expression Omnibus (GEO) database with accession number GSE248684 (https://www.ncbi.nlm.nih.gov/geo/query/acc.cgi?acc=GSE248684). Bulk RNA-Seq data in this paper are available in the NCBI GEO database with accession number GSE247374 (https://www.ncbi.nlm.nih.gov/geo/query/acc.cgi?acc=GSE247374). The codes used for data analyses in this study have been deposited on GitHub under the following link: https://github.com/madoka-iida/Iida-et-al.-2023, commit ID madoka-iida.

## Author contributions

Project planning was performed by M Iida, K Sahashi, and MK; experiments were performed by M Iida, K Sahashi, TH, K Sakakibara, KM, YI, JL, TA, SS, HW, and MK; and data were analyzed by M Iida, K Sahashi, YO, M Iizuka, KH, SS, MN, and MK. The first draft of the manuscript was prepared by M Iida and MK; the text revision was executed by MK.

## Supplementary Material

Supplemental data

Unedited blot and gel images

Supporting data values

## Version 1 06/23/2025

Electronic publication
