# Supplementary material for: Dysregulated synaptic gene expression in oligodendrocytes of spinal and bulbar muscular atrophy
Source: JCI Insight. 2025 Jun 23;10(12):e182123. doi: 10.1172/jci.insight.182123 (PMC12220948; doi:10.1172/jci.insight.182123)
Supplement: Supplemental data [file jciinsight-10-182123-s030.pdf]

Supplementary materials

Dysregulated synaptic gene expression in oligodendrocytes of spinal and bulbar muscular atrophy

Madoka Iida, Kentaro Sahashi, Tomoki Hirunagi, Kenji Sakakibara, Kentaro Maeda, Yohei Iguchi, Jiayi Li, Yosuke Ogura, Masaki Iizuka, Tomohiro Akashi, Kunihiko Hinohara, Shouta Sugio, Hiroaki Wake, Masahiro Nakatochi, Masahisa Katsuno

Supplementary Fig. 1–32
Supplementary Table 1
Supplementary Methods
Supplementary References

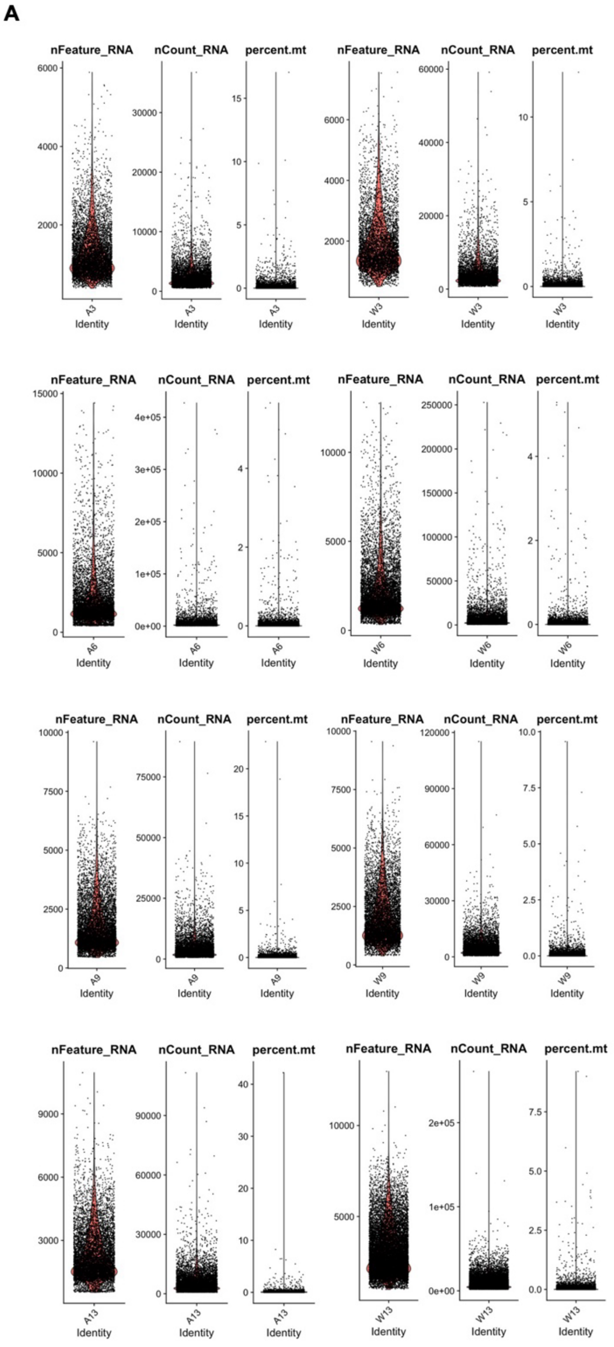

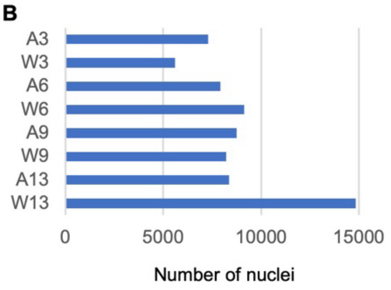

C

Sample	Number of cells
A3	6943
W3	4835
A6	6571
W6	7294
A9	7584
W9	6618
A13	6285
W13	8326

Supplementary Figure 1. Summary of single-nucleus RNA sequencing (snRNA-seq)

A, Number of genes, number of UMI sequences, and proportion of UMI sequences from mitochondrial genes (from left to right) per cell in each sample. **B,** The average number of nuclei of each sample before quality control filtering. **C,** Number of cells for each sample after quality control filtering.

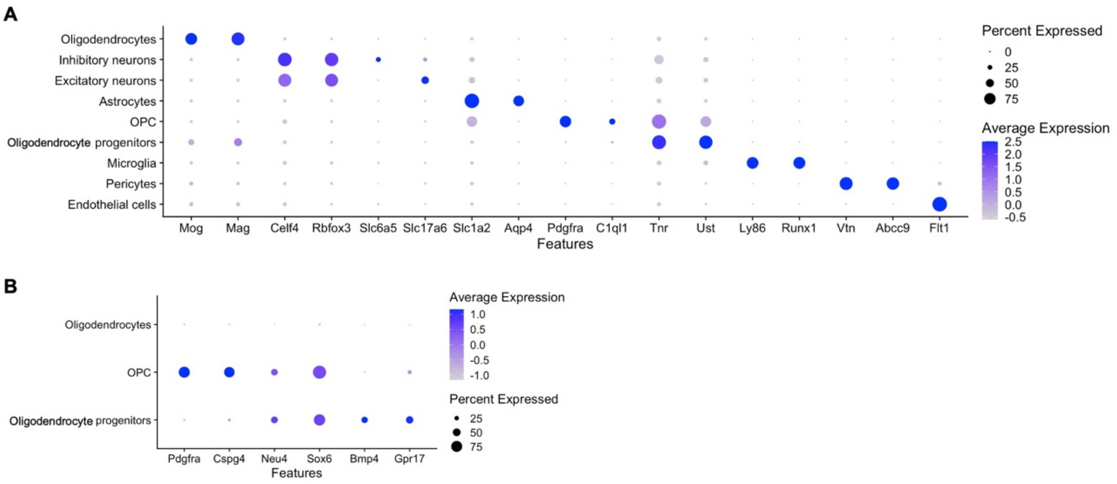

Supplementary Figure 2. snRNA-seq distinguishes major cell types in spinal cord

A, The dot plots of known cell marker genes in each cell type. **B,** The dot plots of known cell marker genes related to oligodendrocyte differentiation in oligodendrocytes, oligodendrocyte precursor cells (OPC), and oligodendrocyte progenitors.

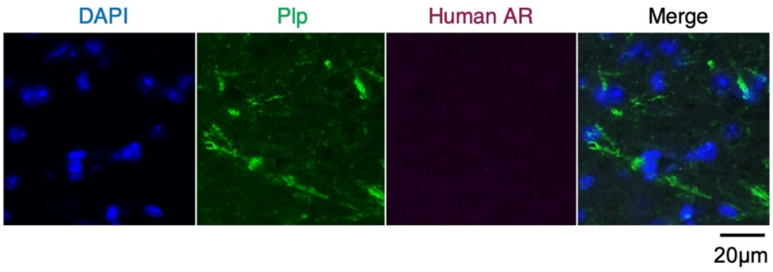

Supplementary Figure 3. Immunofluorescence staining of human AR in the oligodendrocytes of wild-type mice at 3 weeks

Immunostaining of the spinal cord of 3-week-old wild-type mice with antibodies against Plp and human AR. Scale bar: 20 μm.

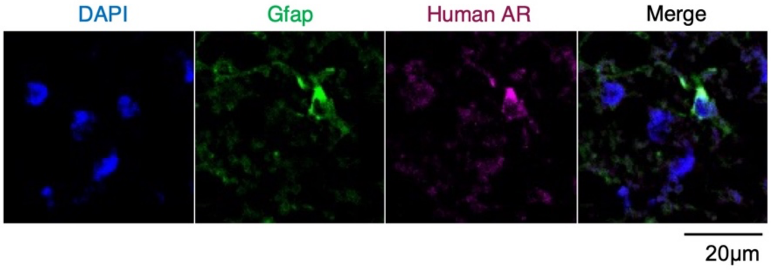

Supplementary Figure 4. Immunofluorescence staining of human AR in the astrocytes of AR-97Q mice at 3 weeks

Immunostaining of the spinal cord of 3-week-old AR-97Q mice with antibodies against Gfap and human AR. Scale bar: 20 μm.

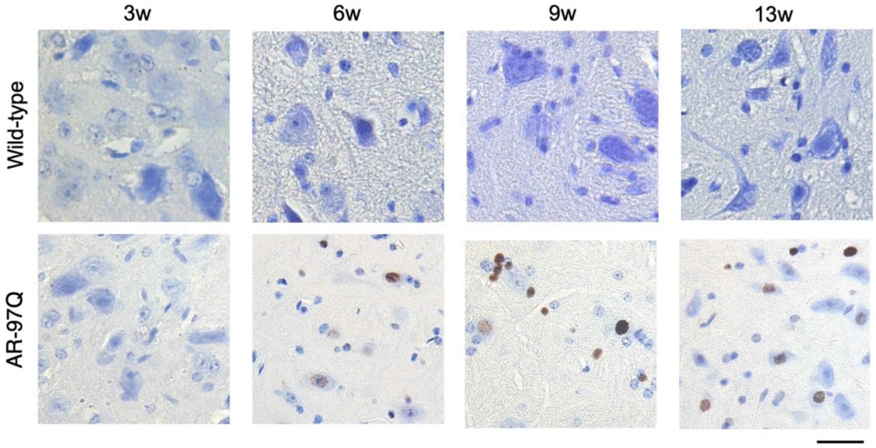

Supplementary Figure 5. Polyglutamine immunoreactivity in the spinal cord of wild-type and AR-97Q mice

Immunostaining of spinal cord from 3-, 6-, 9-, 13-week-old wild-type and AR-97Q mice with an antibody against polyglutamine (1C2). Scale bar: 20 µm.

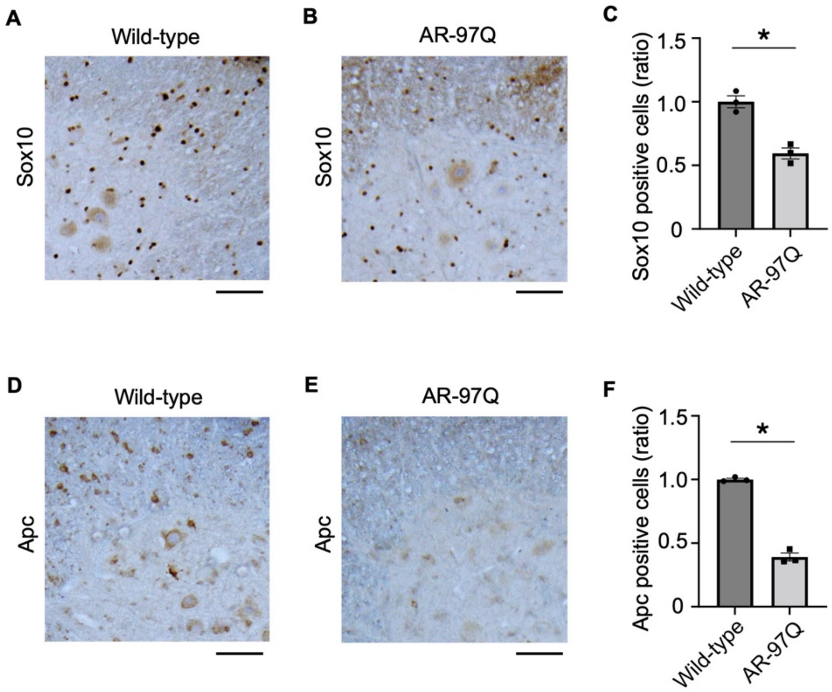

Supplementary Figure 6. The number of Sox10 or Apc positive cells are significantly decreased in the spinal cord of AR-97Q mice at 13 weeks

A–C. Immunostaining of Sox10 in the spinal cord of wild-type (**A**) and AR-97Q mice (**B**) at 13 weeks and quantitative analysis of the staining (**C**). **D–F.** Immunostaining of Apc in the spinal cord of wild-type (**D**) and AR-97Q mice (**E**) at 13 weeks and quantitative analysis of the staining (**F**). Error bars indicate the SEM. *$p < 0.01$, unpaired two-sided t test. Scale bars: 40 μm. N = 3 mice for each group.

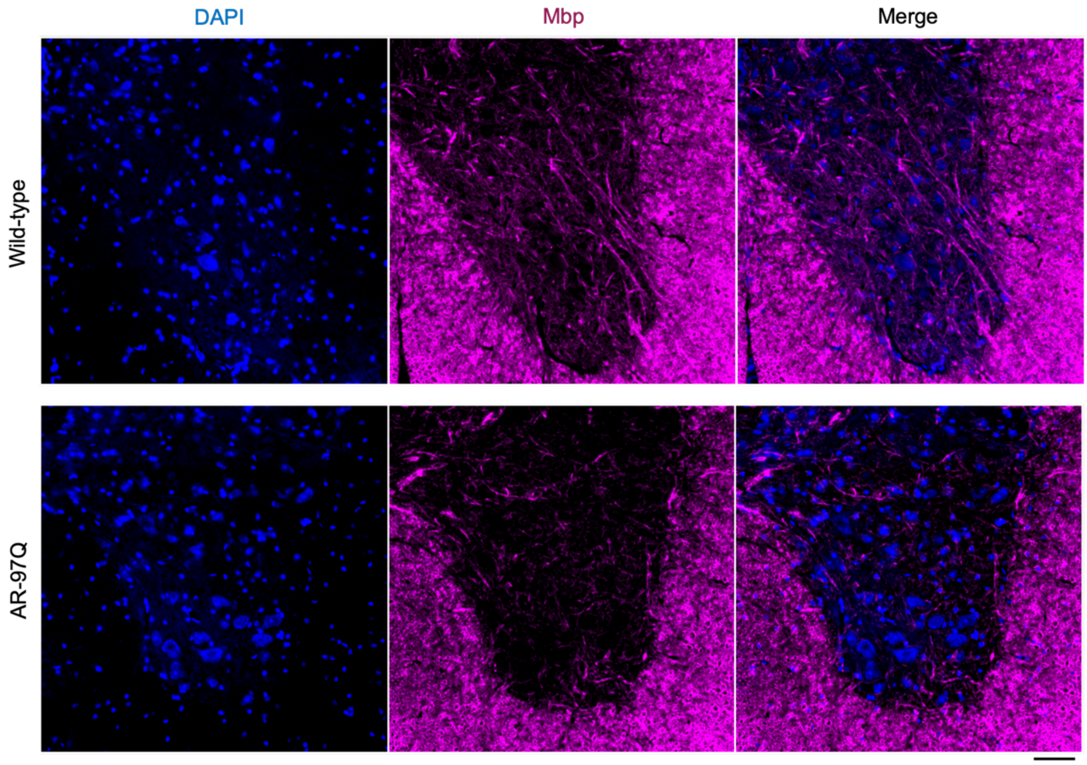

Supplementary Figure 7. Oligodendrocytes are impaired in spinal cords of AR-97Q mice

Immunohistochemical analysis of myelin basic protein (Mbp) in the spinal cords of wild-type and AR-97Q mice at 13 weeks. Scale bar: 50μm.

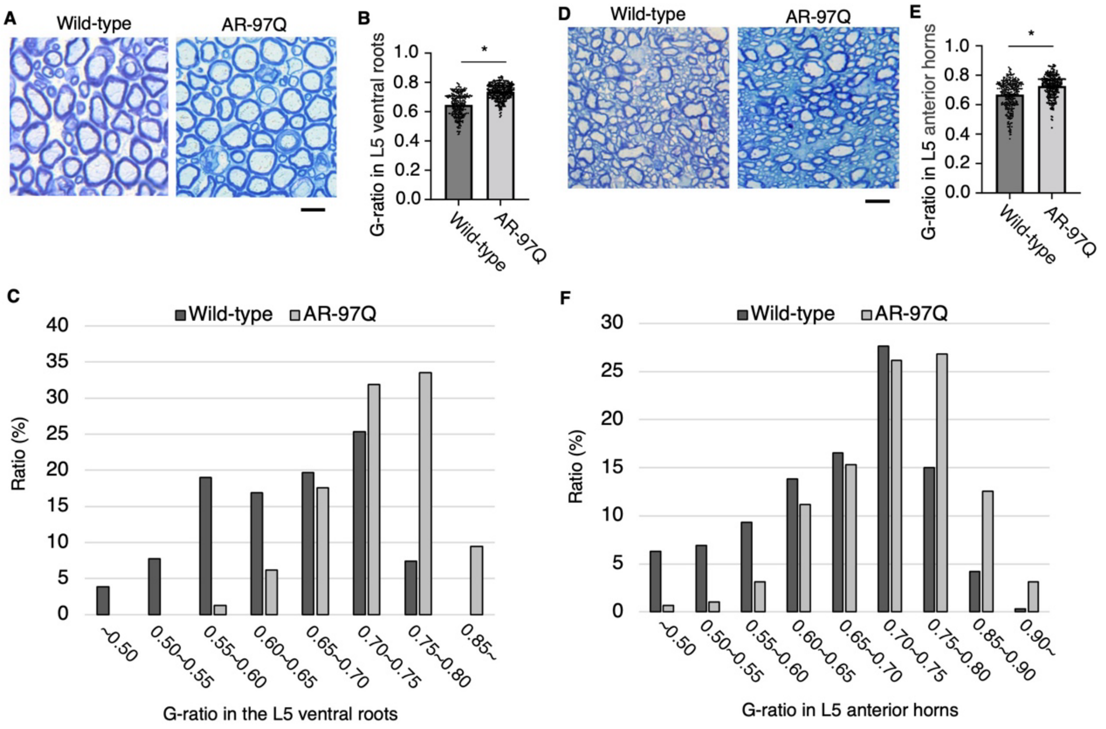

Supplementary Figure 8. The g-ratios of the L5 ventral roots and L5 anterior horns of wild-type and AR-97Q mice at 13 weeks

A. Toluidine blue staining of the L5 ventral root of wild-type and AR-97Q mice. **B.** The average of the g-ratios in the L5 ventral roots of wild-type and AR-97Q mice. **C.** The histogram of the g-ratios in the L5 ventral roots of wild-type and AR-97Q mice. The vertical axis shows the percentage of the total number of nerve fibers measured. **D.** Toluidine blue staining of the L5 anterior horns of wild-type and AR-97Q mice. **E.** The average of the g-ratios in L5 anterior horns of wild-type and AR-97Q mice. **F.** The histogram of the g-ratios in the L5 anterior horns of wild-type and AR-97Q mice. The vertical axis shows the percentage of the total number of nerve fibers measured. Error bars indicate the SEM. *$p < 0.01$, unpaired two-sided t test. Scale bars: 10 µm. N = 3 mice for each group.

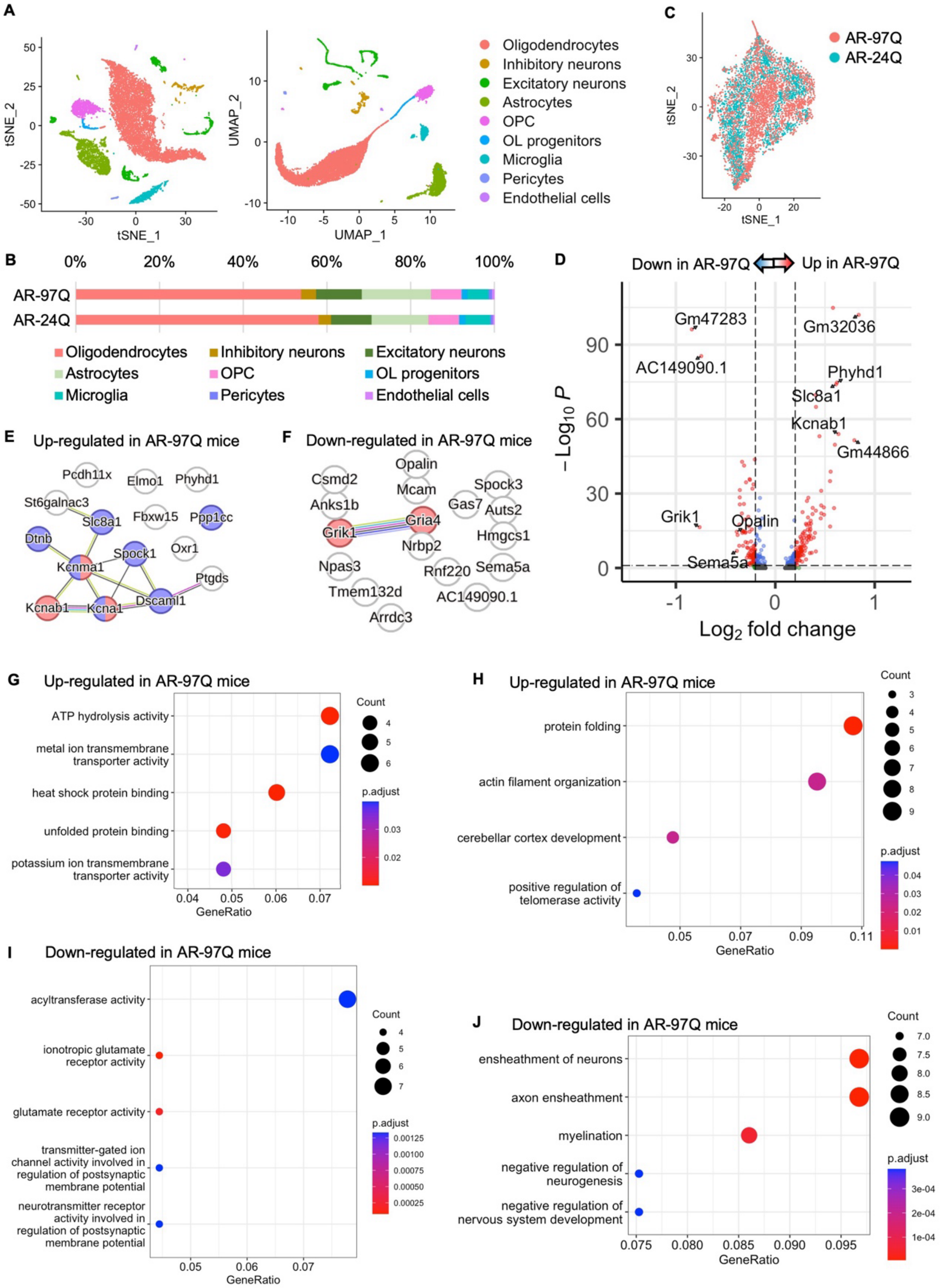

Supplementary Figure 9. Genes associated with ion channels are upregulated in AR-97Q mice compared to AR-24Q mice at 6 weeks

A, t-Distributed stochastic neighbor embedding (t-SNE) and uniform manifold approximation and projection (UMAP) plots visualizing clusters of single nuclei in the spinal

cord of AR-97Q and AR-24Q mice at 6 weeks. **B,** Proportion of the 9 cell types of AR-97Q and AR-24Q mice. **C,** Genotype-colored t-SNE plot of the oligodendrocyte cluster: orange dots represent AR-97Q mice, and green dots represent AR-24Q mice. **D,** Volcano plot showing the DEGs of the oligodendrocyte cluster in AR-97Q and AR-24Q mice. The top 5 genes and last 5 genes are marked. **E,** Protein–protein interaction (PPI) networks for the top 20 upregulated genes in AR-97Q mice. Genes colored in red have voltage-gated potassium channel complex and genes colored in purple have synapse in the cellular component of GO terms. **F,** PPI networks for the top 20 downregulated genes in AR-97Q mice. Genes colored in red have kainate selective glutamate receptor complex in the cellular component of GO terms. **G, H,** The enrichment of the top 100 upregulated genes in AR-97Q mice in the biological process (**G**) and molecular function (**H**) categories ($\log_2$FC > 0.209). **I, J,** The enrichment of the top 100 downregulated genes in AR-97Q mice in the biological process (**I**) and molecular function (**J**) categories ($\log_2$FC < -0.1905). Line color code: sky blue, known interactions from curated databases; magenta, experimentally determined interactions; green, predicted from neighborhood analysis; red, predicted from gene fusions; blue, predicted from gene cooccurrence; pastel green, text mining; black, coexpression; and clear violet, protein homology.

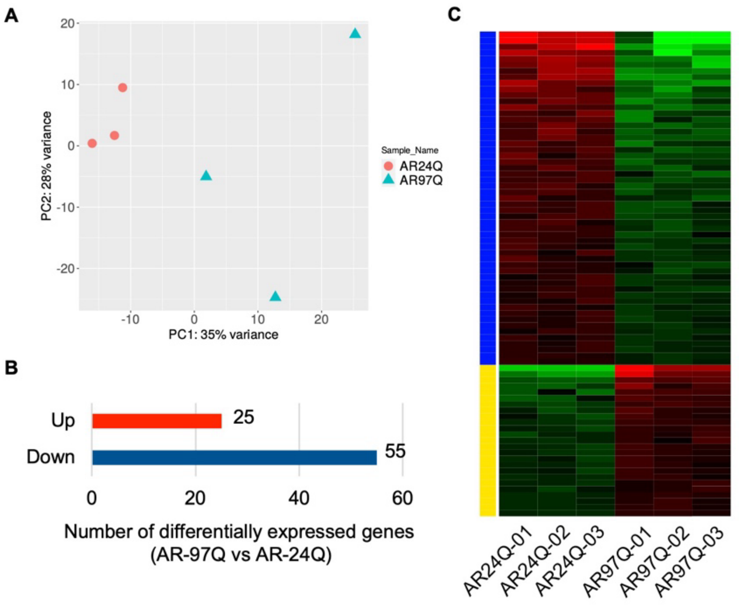

D

Direction	adj.Pval	Pathways
Down regulated	6.0e-03	Collagen fibril organization
	6.0e-03	Leukocyte migration involved in inflammatory response
	6.0e-03	Neutrophil aggregation
	6.5e-03	Ensheathment of neurons
	6.5e-03	Axon ensheathment
	6.5e-03	Myelination
	7.2e-03	L-methionine salvage from methylthioadenosine
	7.8e-03	Tube development
	7.8e-03	Autocrine signaling
	7.8e-03	Amino acid salvage
	7.8e-03	System development
	7.8e-03	Cell chemotaxis
	7.8e-03	L-methionine biosynthetic process
	7.8e-03	L-methionine salvage
Up regulated	4.5e-03	Synapse assembly

E

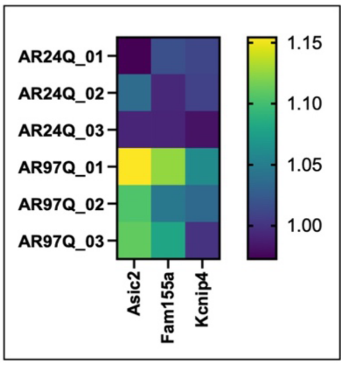

F

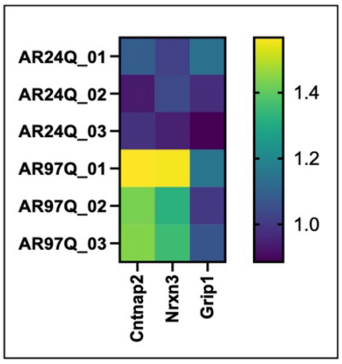

Supplementary Figure 10. Bulk microarray analysis suggests synaptic activation in the spinal cords of AR-97Q mice at the early stages of disease

A, The results of principal component analysis (PCA) for each sample. **B,** Number of differentially expressed genes (FDR < 0.1, FC > 1.5). Red indicates upregulated genes in AR-97Q mice and blue indicates downregulated genes in AR-97Q mice compared to AR-24Q mice. **C,** Heatmap showing results of hierarchical clustering analysis of genes that are significantly altered. **D,** Enriched pathways in DEGs for AR-97Q mice. **E,** Relative expression levels of

channel related genes: *Asic2*, *Fam155a*, and *Kcnip4* in each mouse. **F,** Relative expression levels of genes related to synaptic function: *Cntnap2*, *Nrxn3*, and *Grip1* in each mouse.

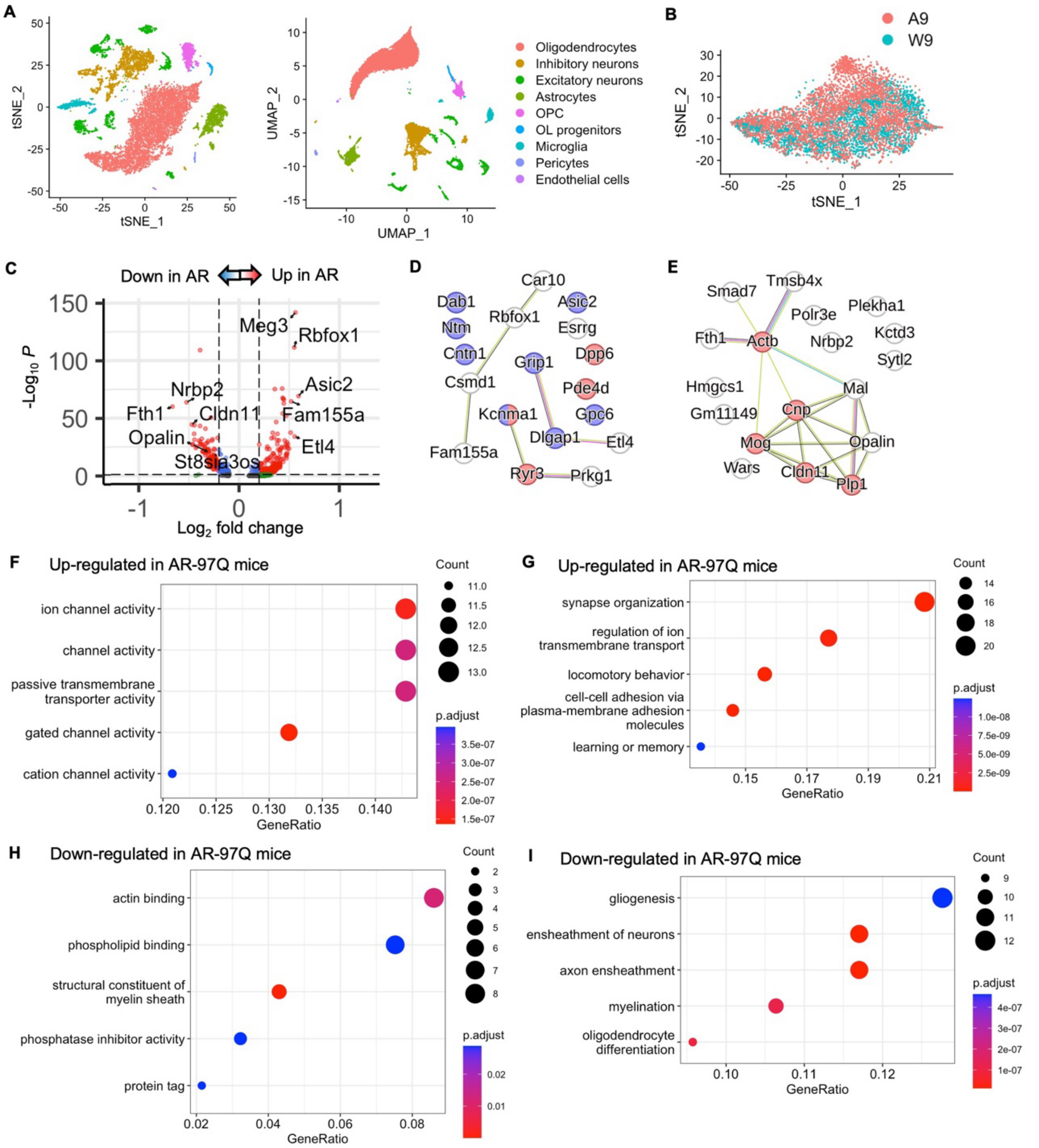

Supplementary Figure 11. Genes associated with synaptic activity are upregulated in AR-97Q mice at 9 weeks

A, t-Distributed stochastic neighbor embedding (t-SNE) and uniform manifold approximation and projection (UMAP) plot visualizing clusters of single nuclei in the spinal cord of AR-97Q and wild-type mice at 9 weeks. **B,** t-SNE plot of the oligodendrocyte cluster colored with the genotype: orange dots represent AR-97Q mice (A9) and green dots represent wild-type mice (W9). **C,** Volcano plot showing DEGs of oligodendrocyte cluster of AR-97Q mice and wild-type mice. The top 5 genes and last 5 genes are marked. **D,** Predicted protein interaction (PPI) networks for the top 20 upregulated genes in AR-97Q mice. Genes colored

in red have cation channel complex and genes colored in purple have synapse in the cellular component of GO terms. **E,** PPI networks for the top 20 downregulated genes in AR-97Q mice. Genes colored in red are related to myelin sheath. **F, G,** The enrichment of the top 100 upregulated genes in AR-97Q mice in the biological process (**F**) and molecular function (**G**) categories ($log_2FC > 0.338$). **H, I,** The enrichment of the top 100 downregulated genes in AR-97Q mice in the biological process (**H**) and molecular function (**I**) categories ($log_2FC < -0.234$). A9, AR-97Q mice at 9 weeks; W9, wild-type mice at 9 weeks.

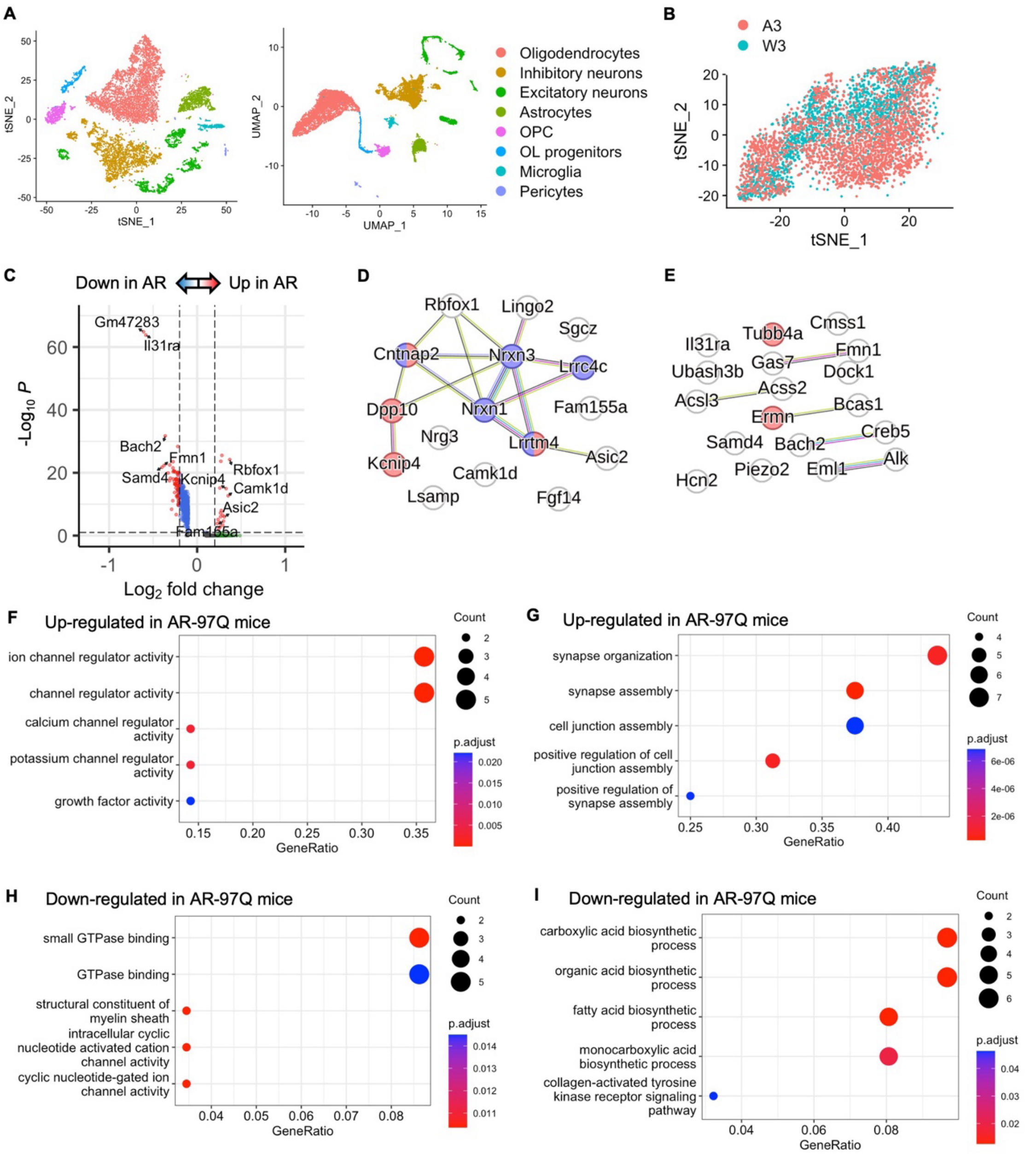

Supplementary Figure 12. Genes associated with synaptic activity are upregulated in AR-97Q mice at 3 weeks

A, t-Distributed stochastic neighbor embedding (t-SNE) and uniform manifold approximation and projection (UMAP) plot visualizing clusters of single nuclei in the spinal cord of AR-97Q and wild-type mice at 3 weeks. **B,** t-SNE plot of the oligodendrocyte cluster colored with the genotype: orange dots represent AR-97Q mice (A3) and green dots represent wild-type mice (W3). **C,** Volcano plot showing DEGs of oligodendrocyte cluster of AR-97Q mice and wild-type mice. The top 5 genes and last 5 genes are marked. **D,** Predicted protein interaction (PPI) networks for the top 17 upregulated genes in AR-97Q mice. Genes colored

in red have cation channel complex and genes colored in purple have synaptic membrane in cellular component of GO terms. **E,** PPI networks for the top 20 downregulated genes in AR-97Q mice. Genes colored in red are related to axons. **F, G,** The GO enrichment of 18 upregulated genes in AR-97Q mice in the biological process (**F**) and molecular function (**G**) categories ($log_2FC > 0.22$). **H, I,** The enrichment of 67 downregulated genes in AR-97Q mice in the biological process (**H**) and molecular function (**I**) categories ($log_2FC < -0.2$). A3, AR-97Q mice at 3 weeks; W3, wild-type mice at 3 weeks.

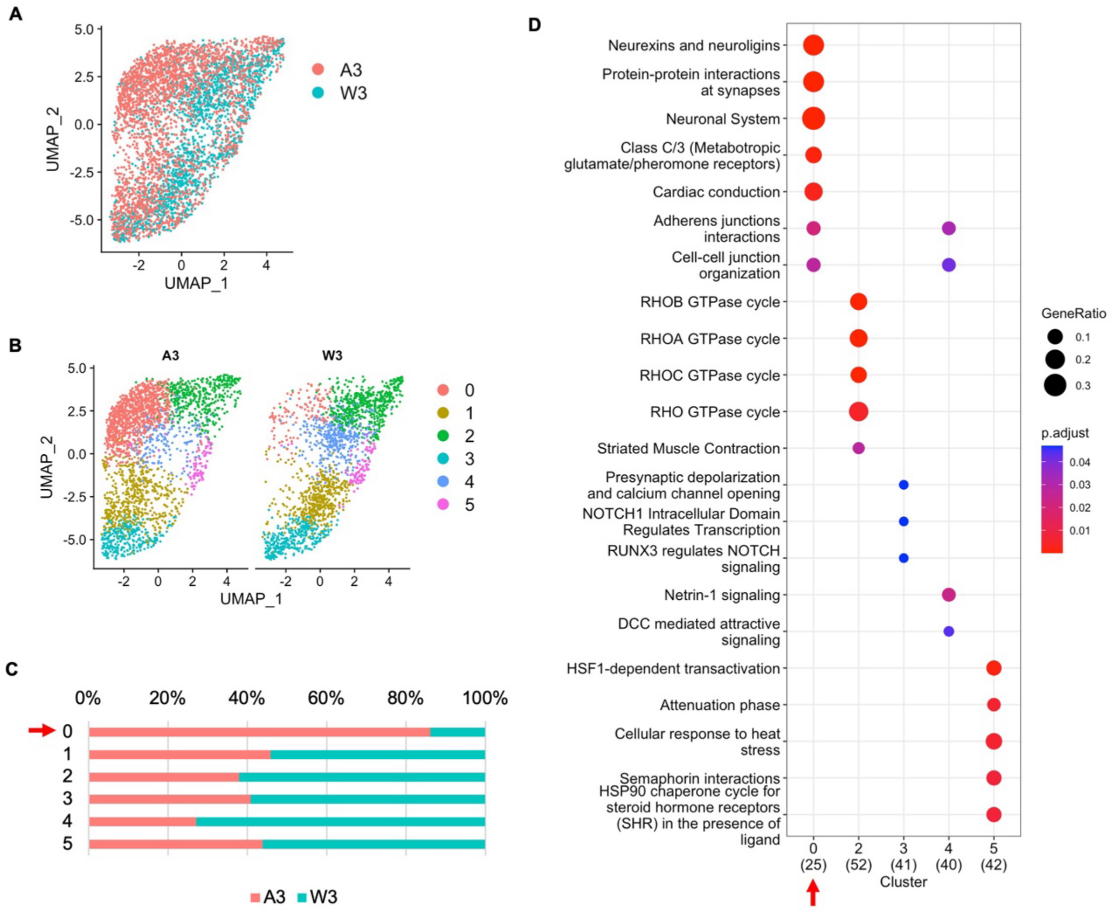

Supplementary Figure 13. Oligodendrocyte heterogeneity at 3 weeks

A, Uniform manifold approximation and projection (UMAP) plot of the oligodendrocyte cluster colored with the genotype: orange dots represent AR-97Q mice (A3) and green dots represent wild-type mice (W3). **B,** UMAP plot of the oligodendrocyte cluster of 3 weeks of age with the associated cell subcluster. **C,** Proportion of each cell subcluster in AR-97Q and wild-type mice. **D,** The enrichment of the Reactome pathway of each subcluster. Red arrows indicate subcluster 0, a cluster dominant in AR-97Q mice.

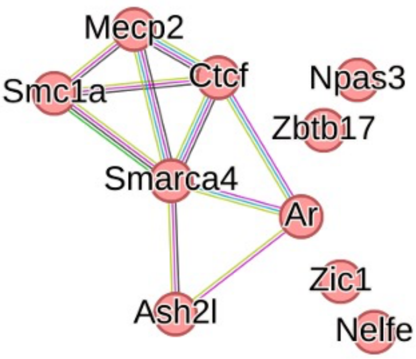

Supplementary Figure 14. PPI networks of transcription factors

Predicted protein interaction (PPI) networks of transcription factors that are related to top 10 and last 10 DEGs of oligodendrocytes of AR-97Q mice at 3 weeks compared to those of wild-type mice at 3 weeks.

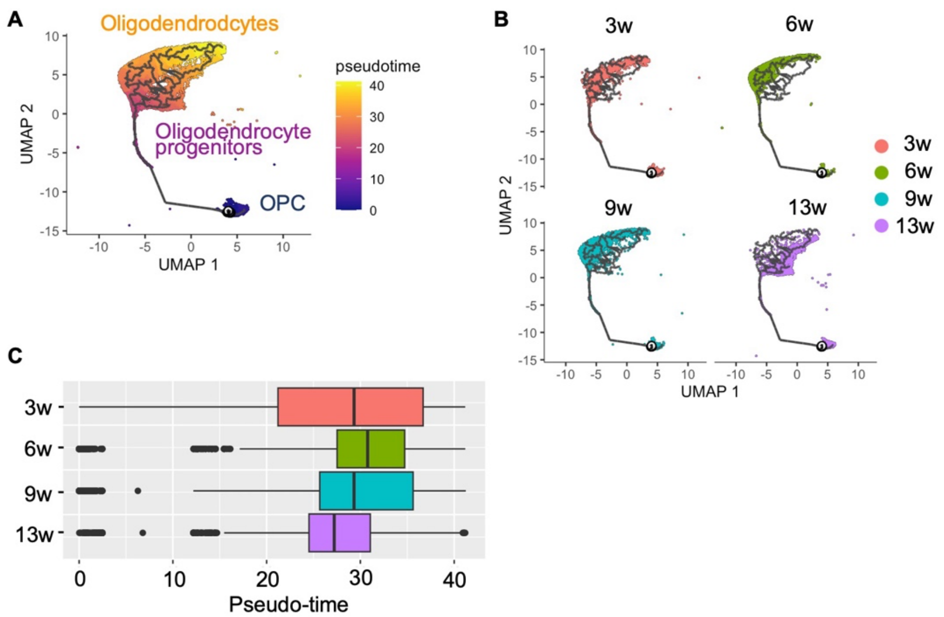

Supplementary Figure 15. Pseudotime analysis based on clusters of oligodendrocyte lineage cells of wild-type mice

A, Pseudotime analysis inferred from clusters of oligodendrocyte lineage cells of wild-type mice at 3, 6, 9, and 13 weeks. **B,** Uniform manifold approximation and projection (UMAP) visualization of clusters of oligodendrocyte lineage cells colored by weeks of age. **C,** Boxplot showing the distribution of pseudotime within each sample. Vertical bars indicate median values.

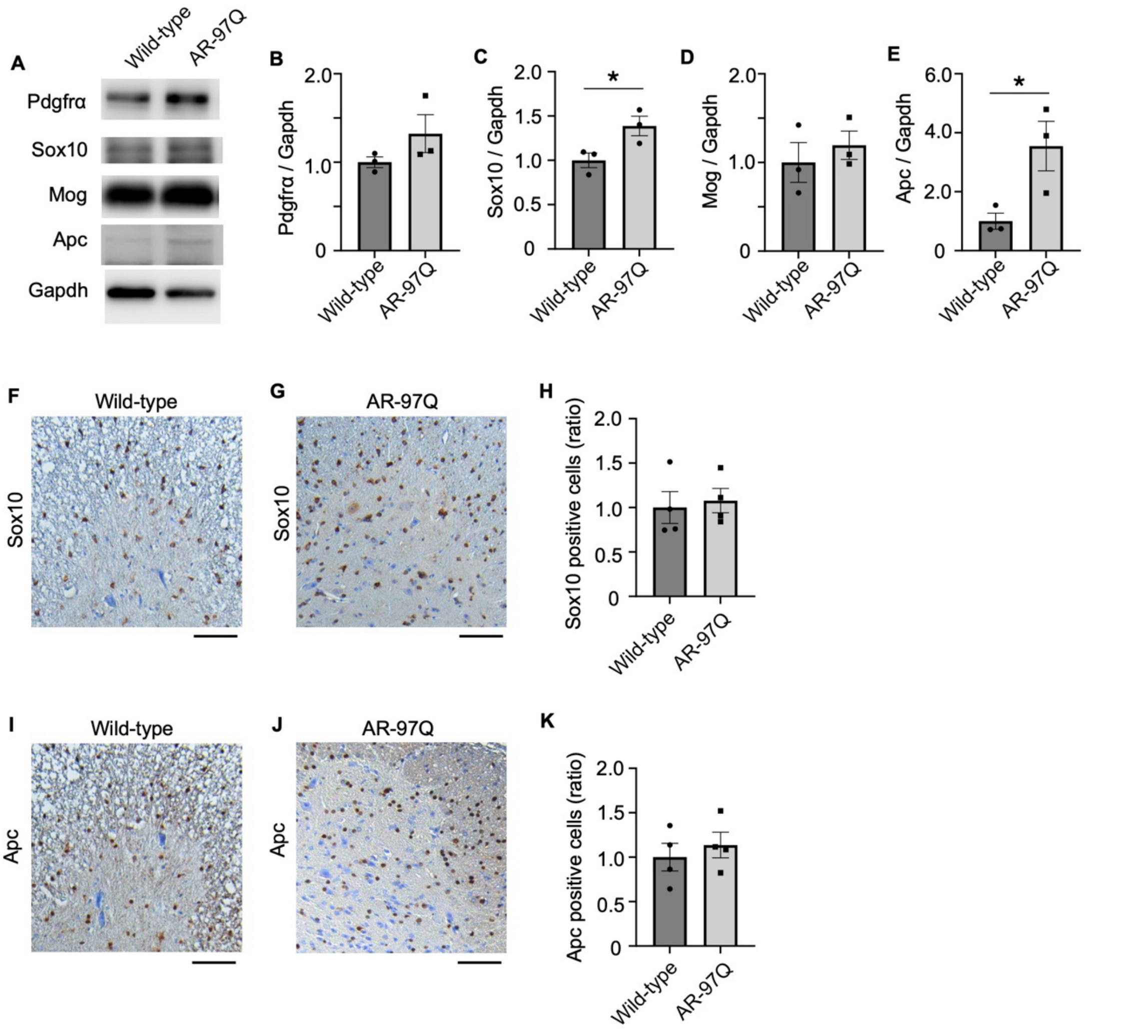

Supplementary Figure 16. The levels of Sox10 and Apc are increased in the spinal cord of AR-97Q mice at 6 weeks

A. Immunoblotting of Pdgfrα, Sox10, Mog, and Apc in the spinal cord of wild-type and AR-97Q mice at 6 weeks. **B–E.** Quantitative immunoblot analysis of Pdgfrα (**B**), Sox10 (**C**), Mog (**D**), and Apc (**E**) in the spinal cord of wild-type and AR-97Q mice at 6 weeks. **F–H.** Immunostaining of Sox10 in the spinal cord of wild-type (**F**) and AR-97Q mice (**G**) at 6 weeks and quantitative analysis of the staining (**H**). **I–K.** Immunostaining of Apc in the spinal cord of wild-type (**I**) and AR-97Q mice (**J**) at 6 weeks and quantitative analysis of the staining (**K**). Error bars indicate the SEM. *$p < 0.05$, unpaired two-sided t test. Scale bars: 40 μm. N = 3 mice per group for immunoblotting analysis (**B–E**) and N = 4 mice per group for immunostaining analysis (**H, K**).

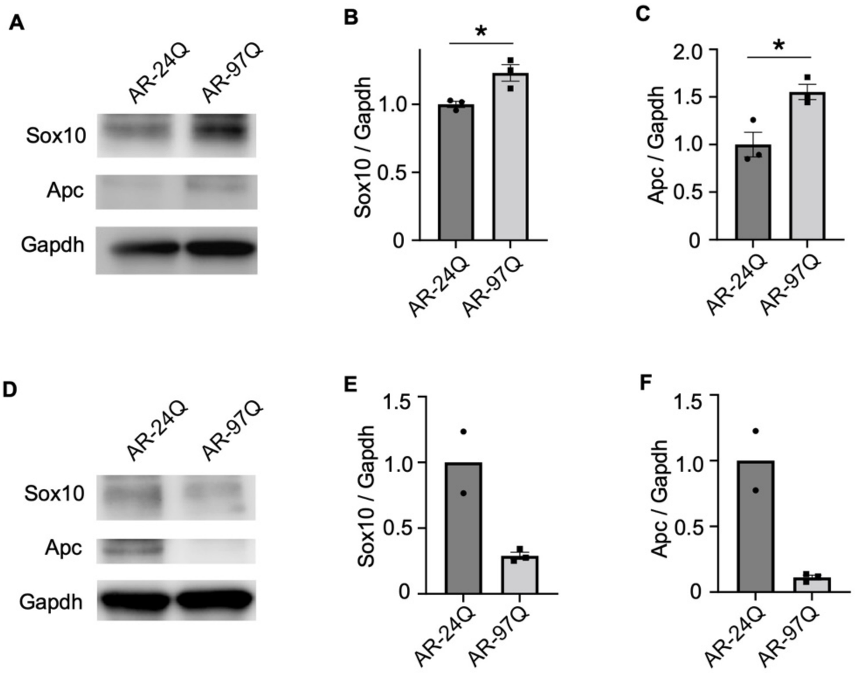

Supplementary Figure 17. The change of Sox10 and Apc protein levels in the spinal cord of AR-97Q mice compared to AR-24Q mice

A. Immunoblotting of Sox10 and Apc in the spinal cord of AR-24Q and AR-97Q mice at 6 weeks. **B, C.** Quantitative immunoblot analysis of Sox10 (**B**) and Apc (**C**) in the spinal cord of AR-24Q (N = 3) and AR-97Q mice (N = 3) at 6 weeks. **D.** Immunoblotting of Sox10 and Apc in the spinal cord of AR-24Q and AR-97Q mice at 15 weeks. **E, F.** Quantitative immunoblot analysis of Sox10 (**E**) and Apc (**F**) in the spinal cord of AR-24Q (N = 2) and AR-97Q mice (N = 3) at 15 weeks. Error bars indicate the SEM. *$p < 0.05$, unpaired two-sided t test.

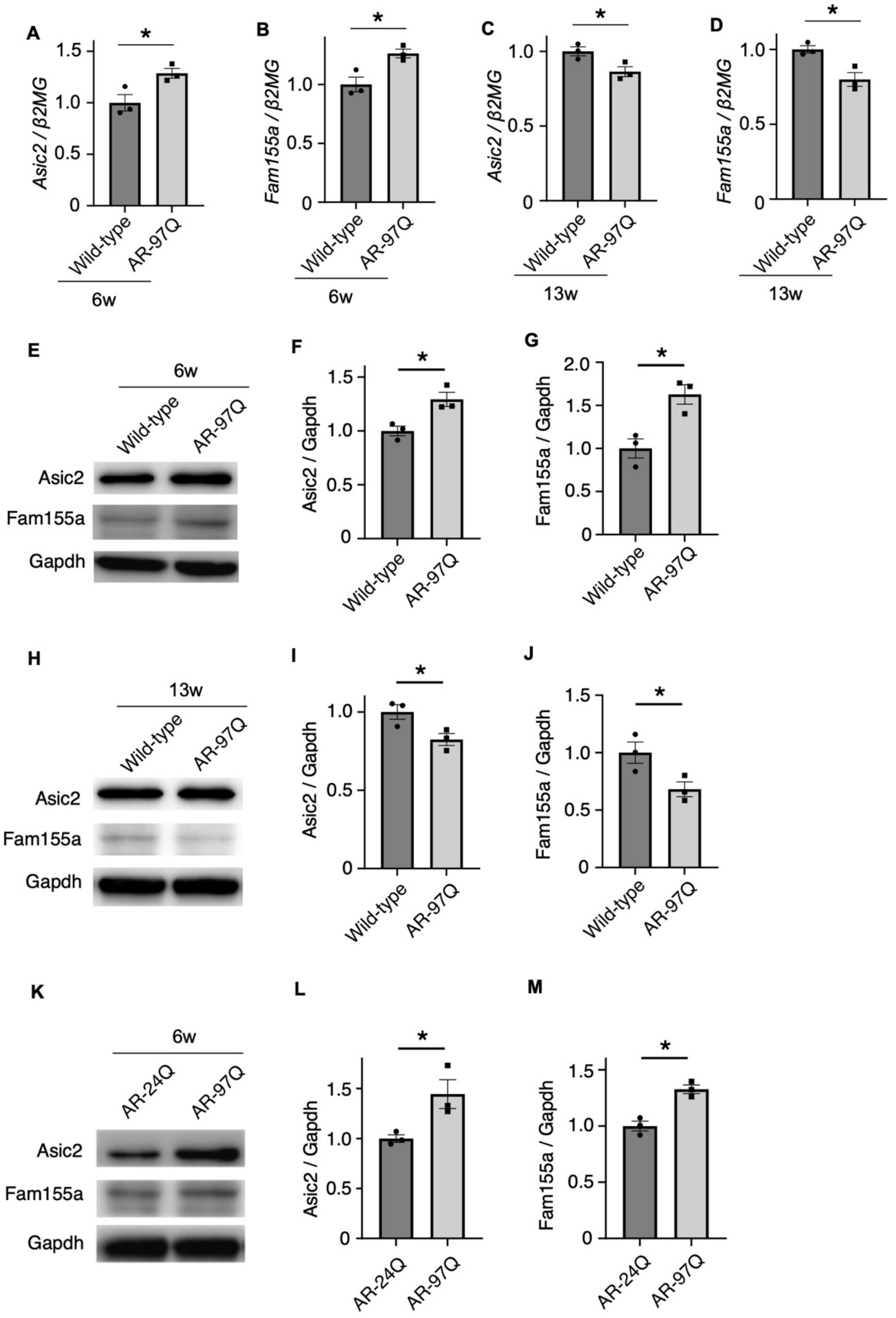

Supplementary Figure 18. The mRNA and protein levels of Asic2 and Fam155a are elevated in the spinal cord of AR-97Q mice at 6 weeks

A, B. Quantitative real-time polymerase chain reaction (RT-PCR) analysis of *Asic2* (**A**) and *Fam155a* (**B**) mRNA in the spinal cord of wild-type and AR-97Q mice at 6 weeks. **C, D.** Quantitative RT-PCR analysis of *Asic2* (**C**) and *Fam155a* (**D**) mRNA in the spinal cord of wild-type and AR-97Q mice at 13 weeks. **E.** Immunoblotting of Asic2 and Fam155a in the spinal cord of wild-type and AR-97Q mice at 6 weeks. **F, G.** Quantitative immunoblot analysis of Asic2 (**F**) and Fam155a (**G**) in the spinal cord of wild-type and AR-97Q mice at 6 weeks. **H.** Immunoblotting of Asic2 and Fam155a in the spinal cord of wild-type and AR-97Q mice at 13 weeks. **I, J.** Quantitative immunoblot analysis of Asic2 (**I**) and Fam155a (**J**) in the spinal cord of wild-type and AR-97Q mice at 13 weeks. **K.** Immunoblotting of Asic2 and Fam155a in the spinal cord of AR-24Q and AR-97Q mice at 6 weeks. **L, M.** Quantitative immunoblot analysis of Asic2 (**L**) and Fam155a (**M**) in the spinal cord of AR-24Q and AR-97Q mice at 6 weeks. Error bars indicate the SEM. *$p < 0.05$, unpaired two-sided t test. N = 3 mice per group.

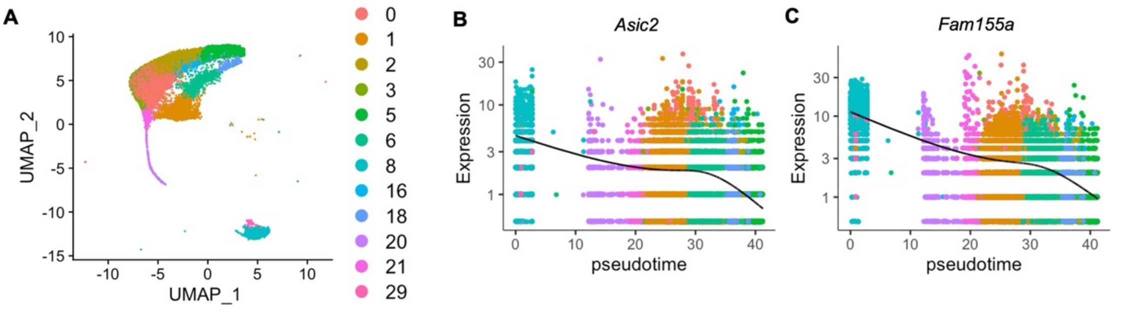

Supplementary Figure 19. Pseudotime kinetics of *Asic2* and *Fam155a* in oligodendrocyte lineage cells of AR-97Q and wild-type mice

A, Uniform manifold approximation and projection (UMAP) visualization of clusters of oligodendrocyte lineage cells of wild-type mice at 4 stages colored by Seurat package (resolution = 1.2). **B, C,** Pseudotime kinetics of *Asic2* (**B**), and *Fam155a* (**C**).

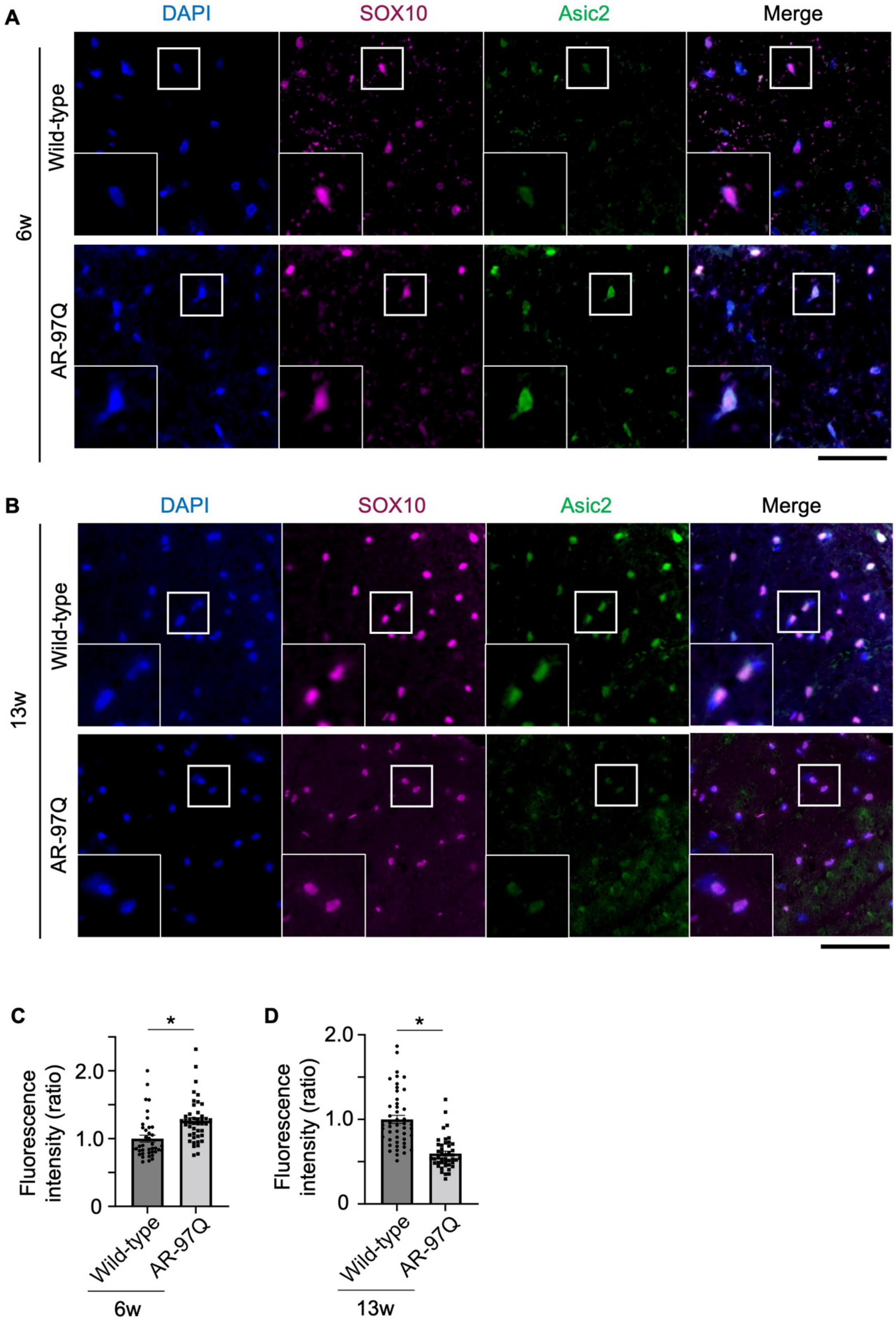
A
DAPI
SOX10
Asic2
Merge
6w
Wild-type
AR-97Q
B
DAPI
SOX10
Asic2
Merge
13w
Wild-type
AR-97Q
C
Fluorescence intensity (ratio)
0
1.0
2.0
*
Wild-type
AR-97Q
6w
D
Fluorescence intensity (ratio)
0
1.0
2.0
*
Wild-type
AR-97Q
13w

212

Supplementary Figure 20. Asic2 levels in oligodendrocytes of spinal cords of AR-97Q mice are upregulated at 6 weeks and downregulated at 13 weeks

A, B, Immunofluorescence staining of spinal cords of wild-type mice and AR-97Q mice at 6 weeks (**A**) and 13 weeks (**B**). **C, D.** Fluorescence intensities of Asic2 in the spinal cords of wild-type mice and AR-97Q mice at 6 weeks (**C**) and 13 weeks (**D**). Error bars indicate the SEM. *$p < 0.01$, unpaired two-sided t test. Scale bars: 50 μm. N=3 per group.

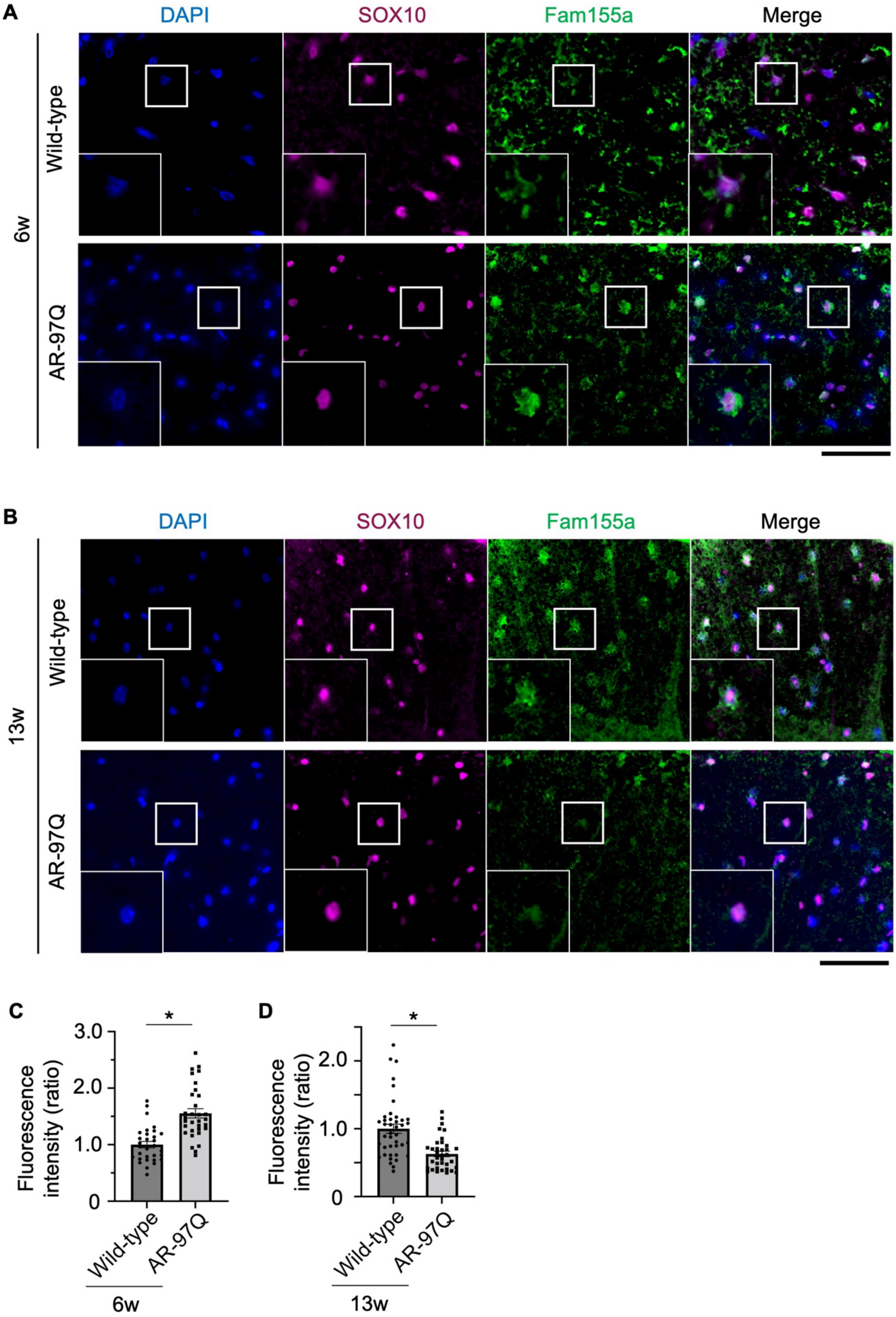
A
DAPI
SOX10
Fam155a
Merge
6w
Wild-type
AR-97Q
B
DAPI
SOX10
Fam155a
Merge
13w
Wild-type
AR-97Q
C
Fluorescence intensity (ratio)
3.0
2.0
1.0
0
*
Wild-type
AR-97Q
6w
D
Fluorescence intensity (ratio)
2.0
1.0
0
*
Wild-type
AR-97Q
13w

220

Supplementary Figure 21. Fam155a levels in oligodendrocytes of spinal cords of AR-97Q mice are upregulated at 6 weeks and downregulated at 13 weeks

A, B. Immunofluorescence staining of spinal cords of wild-type mice and AR-97Q mice at 6 weeks (**A**) and 13 weeks (**B**). **C, D.** Fluorescence intensities of Fam155a in spinal cords of wild-type mice and AR-97Q mice at 6 weeks (**C**) and 13 weeks (**D**). Error bars indicate the SEM. *$p < 0.01$, unpaired two-sided t test. Scale bars: 50 μm. N=3 per group.

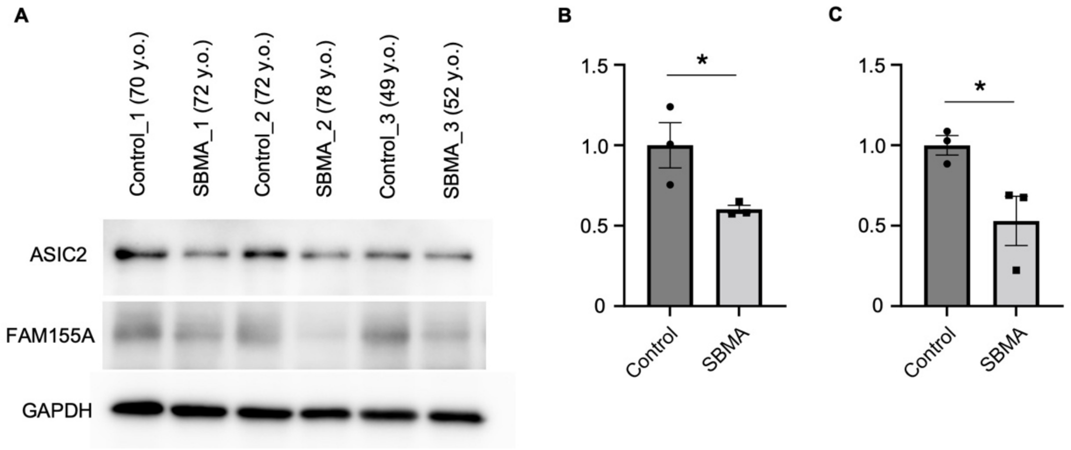

Supplementary Figure 22. The levels of ASIC2 and FAM155A are suppressed in the spinal cord of patients with SBMA

A. Immunoblotting of ASIC2 and FAM155A in autopsied spinal cords in disease controls and SBMA subjects. All subjects are males. **B, C.** Quantitative immunoblot analysis of ASIC2 (**B**) and FAM155A (**C**) in the spinal cords of control and SBMA subjects. Error bars indicate the SEM. *$p < 0.05$, unpaired two-sided t test.

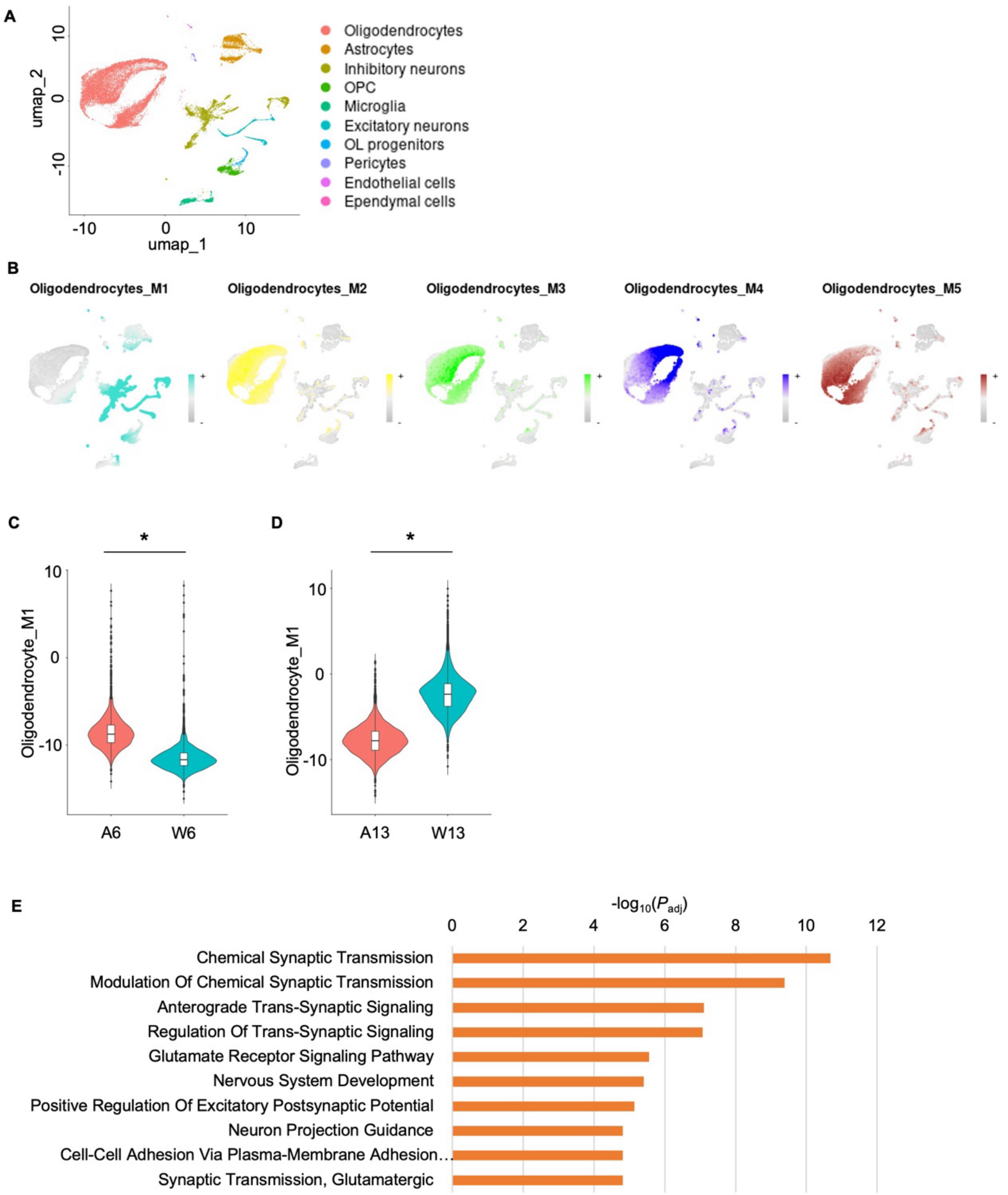

Supplementary Figure 23. High-dimensional weighted gene co-expression network analysis (hdWGCNA) using oligodendrocyte data identifies a module related to synaptic transmission

A. Uniform manifold approximation and projection (UMAP) plots visualizing clusters of single nuclei in the spinal cord of AR-97Q and wild-type mice at 6 and 13 weeks. **B.** UMAP plots of single cell data, colored by module eigengene of each module. **C, D.** Comparison of module eigengene expression between AR-97Q and wild-type mice in the M1 module at 6 weeks (**C**) and 13 weeks (**D**). **E.** Top 10 Gene Ontology (GO) terms with the lowest *p*-value in

the category of biological processes enriched in the top 100 genes contained in M1 modules. *P*-values were adjusted for FDR. Wilcoxon rank sum test, $*p < 0.001$.

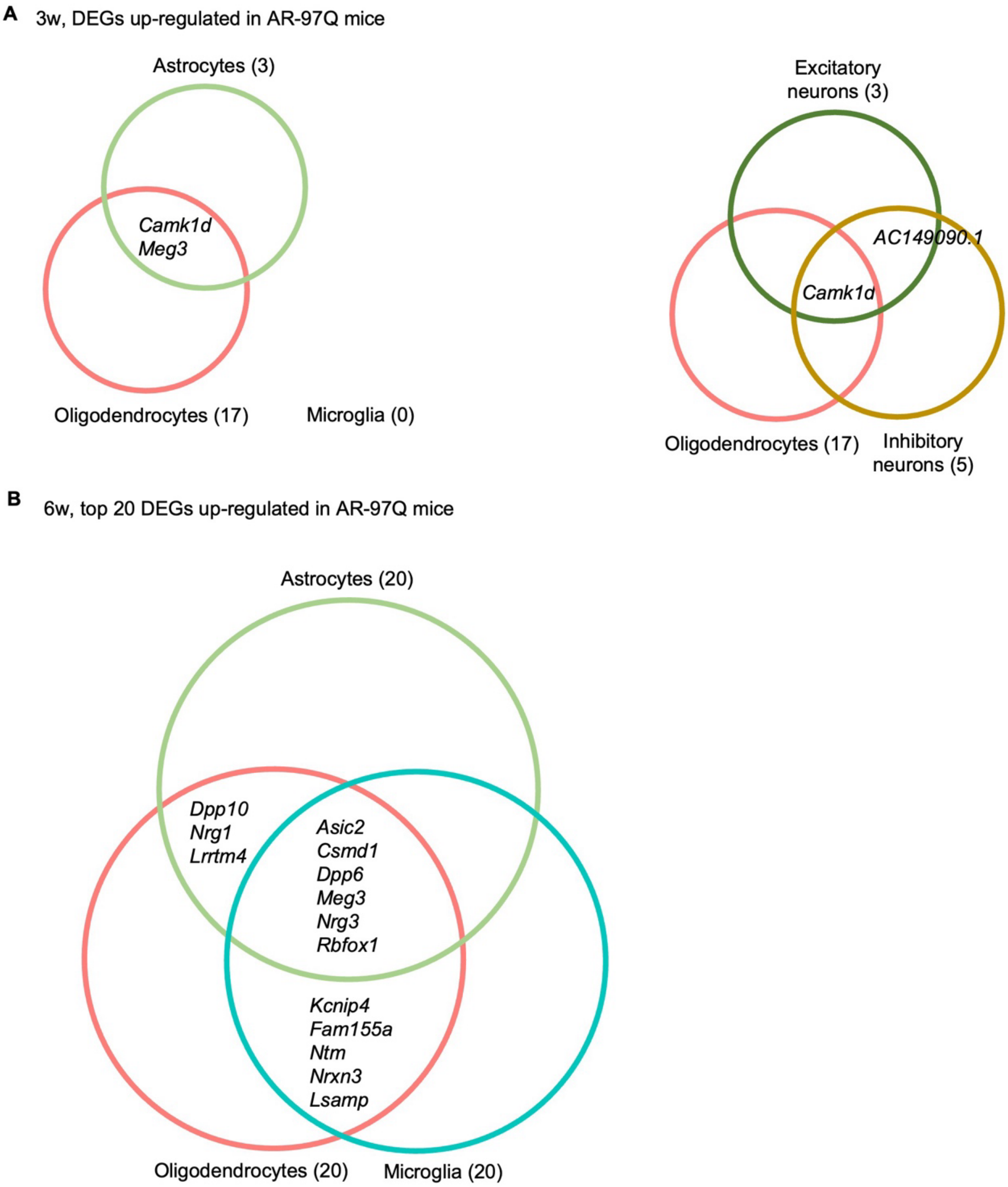

Supplementary Figure 24. Top DEGs that are common across cell types

A, Common DEGs upregulated in AR-97Q mice in oligodendrocytes and astrocytes (left), and in oligodendrocyte s, inhibitory neurons, and excitatory neurons (right) at 3 weeks. **B,** Common top 20 DEGs across oligodendrocytes, astrocytes, and microglia at 6 weeks. Numbers in parentheses indicate the number of DEGs.

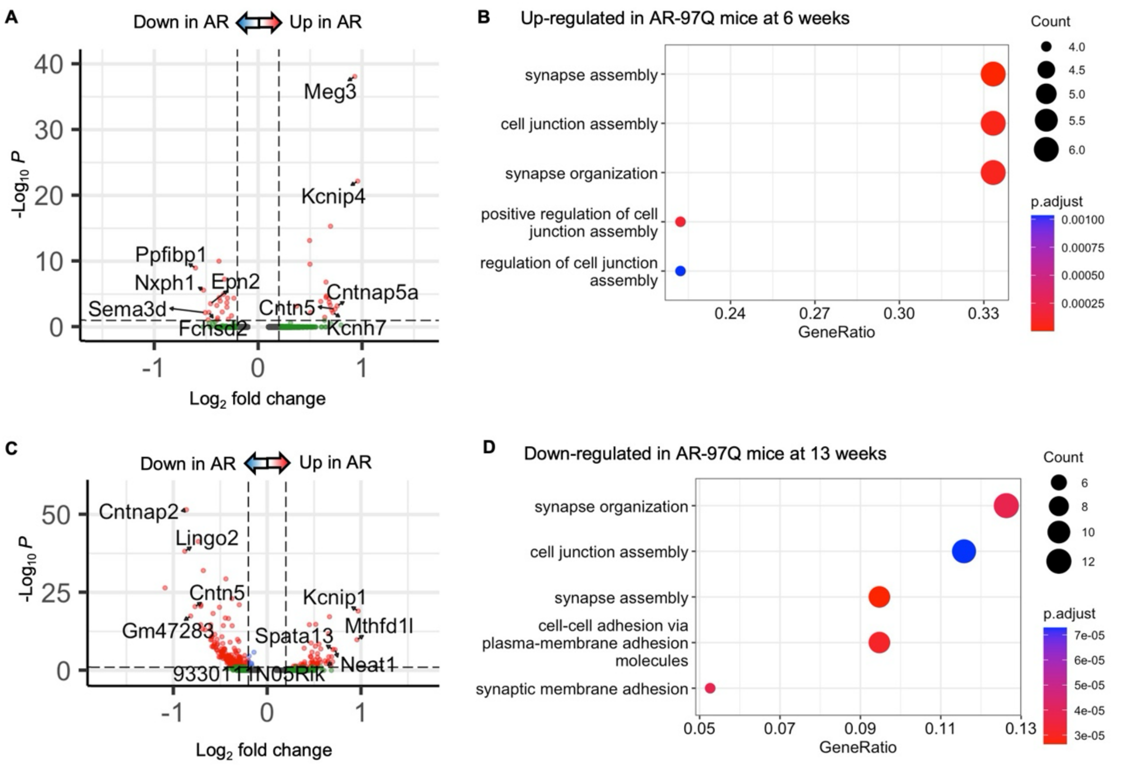

Supplementary Figure 25. Disease-dependent DEGs of OPC at 6 and 13 weeks

A, Volcano plot showing differential gene expression of the oligodendrocyte precursor cells (OPC) cluster of AR-97Q mice and wild-type mice at 6 weeks. The top 5 genes and last 5 genes are marked. **B,** The enrichment of 18 upregulated genes in AR-97Q mice at 6 weeks in the biological process category ($log_2FC > 0.37$). **C,** Volcano plot showing differential gene expression of OPC cluster of AR-97Q mice and wild-type mice at 13 weeks. The top 5 genes and last 5 genes are marked. **D,** The enrichment of 100 downregulated genes in AR-97Q mice at 13 weeks in the biological process category ($log_2FC < -0.368$).

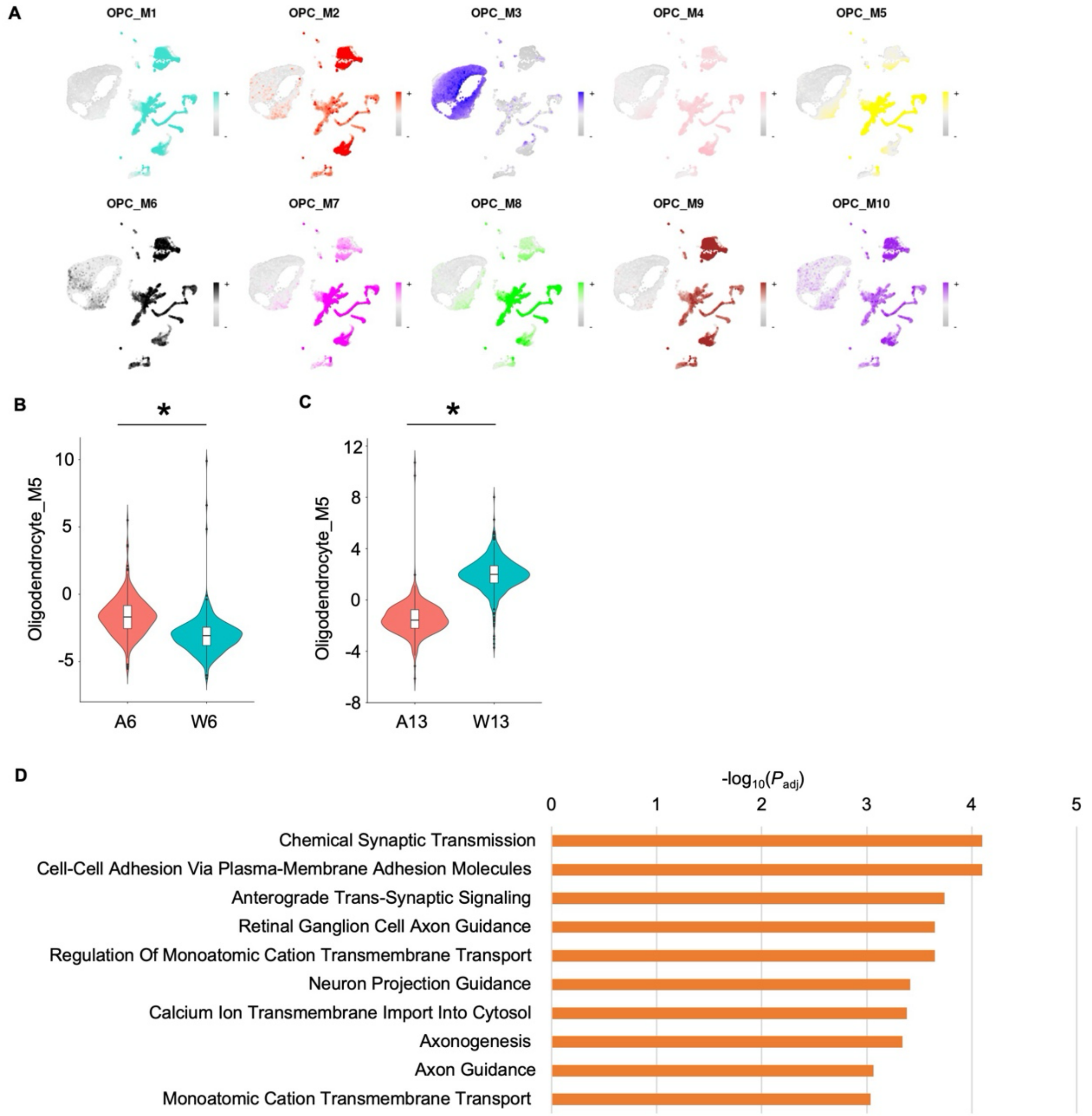

Supplementary Figure 26. High-dimensional weighted gene co-expression network analysis (hdWGCNA) using OPC data identifies a module related to synaptic transmission

A. UMAP plots of single cell data, colored by module eigengene of each module. **B, C.** Comparison of module eigengene expression between AR-97Q and wild-type mice in the M5 module at 6 weeks (**B**) and 13 weeks (**C**). **D**. Top 10 Gene Ontology (GO) terms with the lowest *p*-value in the category of biological processes enriched in the top 100 genes contained in M5 modules. *P*-values were adjusted for FDR. Wilcoxon rank sum test, *$p < 0.001$.

A

3w	6w	9w	13w
AC149090.1	Rnf220	St18	Gm42418
Camk1d	Plcl1	Taco1	Gm26691
Gm42418	Mobp	Slc24a2	AC149090.1
	Gm47283	Cdh20	Taco1
	Plp1	Zeb2	Cmss1
	St6galnac3	Kcnq1ot1	Garnl3
	Etl4	AC149090.1	Tcf25
	Prr5l	Grip1	Elavl3
	Gm42418	Pcdh9	Srrm2
	St18	Pde4b	Rasgrf1
	AC149090.1	Nkain2	Zkscan2
	Tmeff2		Ebf3
	Taco1		Golgb1
	Mast4		1500004A13Rik
	Dscaml1		Capn2
	Gpc5		Zfp810
	Frmd5		Tubgcp5
	Nkain2		Scg5
	Fstl5		Gm41361
	Gm26917		Grin1
	Pcdh9		
	Mapt		
	Cmss1		
	Lrrtm4		

B

3w	6w	9w	13w
Gm47283	Syn2	Sst	Plp1
Mid1	Prkg1	A330008L17Rik	Qk
Il31ra	Sst	Pcsk1n	Gm47283
Setx	Cacna2d1	Atp6v0c	Nxph1
Gm48099	Gm32509	Nrbp2	Htr2c
Cck	Gabra2	Setx	Gpc5
A830082K12Rik	Nrg3os	Igsf8	Hsph1
Lars2	Setx	AY036118	Slit2
Cmss1	Ebf1	Ly6h	Nrxn3
Ppm1a	Osbpl8	Prnp	Ptpn9
Hacd1	Gabrb3	Slc22a17	Tmeff2
Khdrbs3	4930438E09Rik	Calm1	Nr6a1
Cfap54	Gm15261	Pura	Stxbp6
Ptger3	B230334C09Rik	Baiap3	Hspa4l
Gm13986	Mme	Atp6v0b	Sgcz
Asph	5530401A14Rik	Gm15738	Stip1
Tmem181a	Ssbp2	Grin1	Pcdh9
Kif3a	Gm15398	Ddx17	Hsp90aa1
Armc9	Dlg2	Hsph1	Fmnl2
Taco1	Nrg3	Sdf4	Ppm1e
Cdkal1	Lncpint	Snhg11	Ext1
Aebp2	Gm15738	6430628N08Rik	Tcf12
Dzip3	Gm16599		Gabra2
Ttc9	Nrxn1		Cdh20
Ldlr	Gm5441		Celf1
4632427E13Rik	Adgrl3		Banp
Kit	Lsamp		Strbp
Dnah7c	Hnrnpdl		P4ha1
Klf7	Fgf14		Top1
Lrrc3b	Gm20642		Ugt8a

Supplementary Figure 27. The lists of significantly upregulated or downregulated genes in excitatory neurons of AR-97Q mice compared to wild-type mice

A, B. Upregulated (**A**) or downregulated (**B**) genes in excitatory neurons of AR-97Q mice compared to wild-type mice. Adjusted $p < 0.05$, up to 30th.

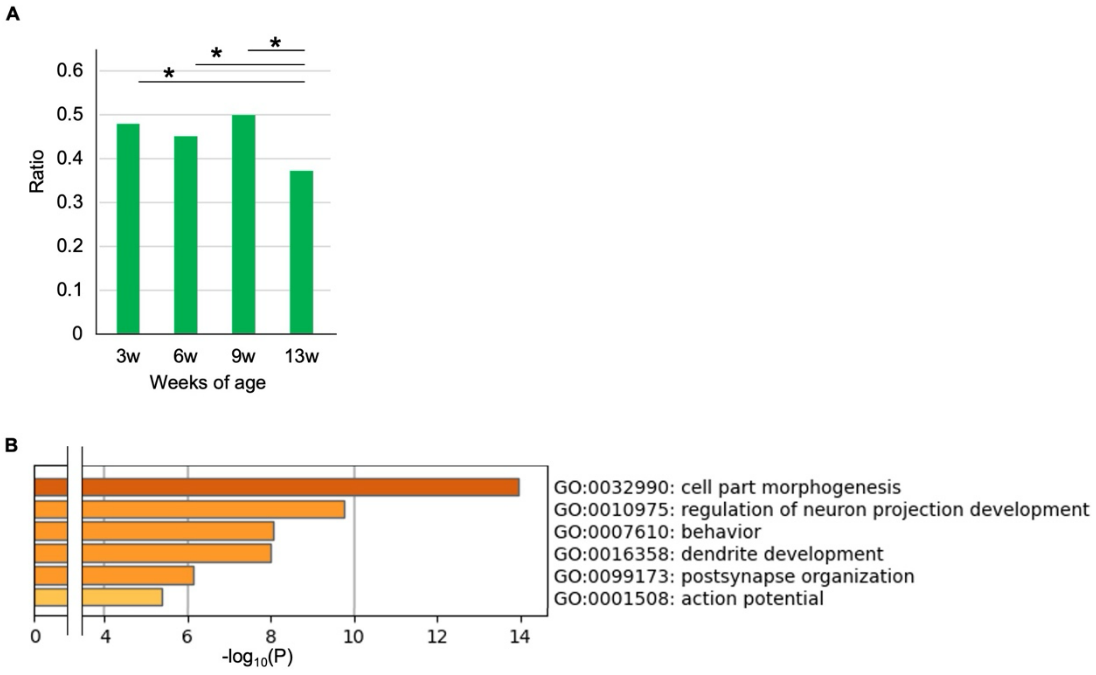

Supplementary Figure 28. Comparison of the RNA-seq data of oligodendrocyte cell model of SBMA and oligodendrocyte data from snRNA-seq

A, Ratio of the number of common genes upregulated or downregulated in both cell and mouse model versus the number of total genes that were measured the expression levels by both RNA-seqs. **B,** GO terms ($-\log_{10}(P) > 5$) of genes that are among DEGs upregulated in oligodendrocytes of AR-97Q mice ($\log_2 FC > 0.1$) at 6 weeks and among genes upregulated in AR-97Q cells compared to AR-24Q cells. *$p < 0.001$, equality of proportions hypothesis test.

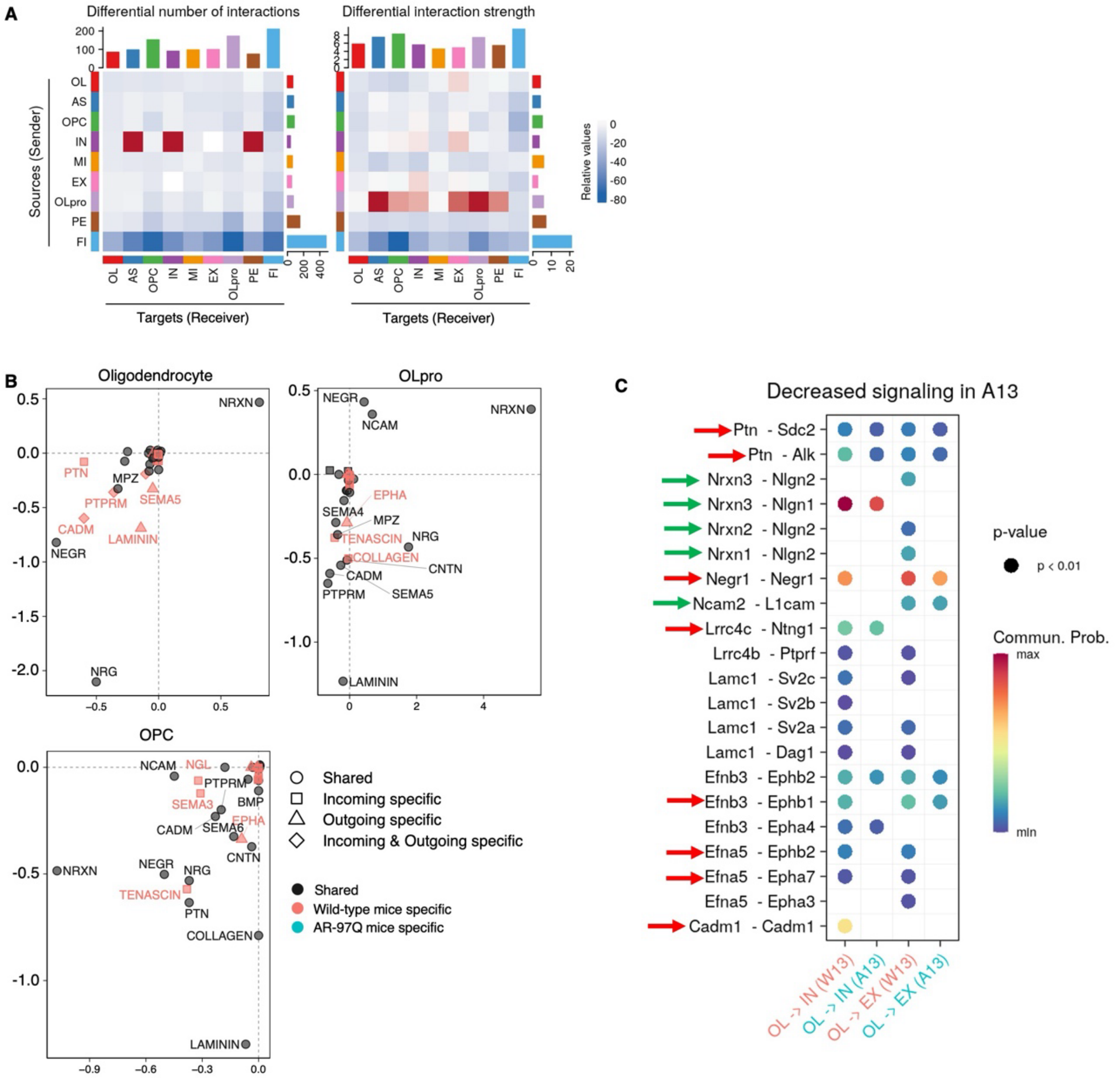

Supplementary Figure 29. Interaction strength between oligodendrocytes and neurons is decreased at the advanced stages of SBMA

A, Heatmap of differential interaction strength in AR-97Q mice compared to wild-type mice at 13 weeks. The top colored bar plot represents the sum of the values in columns displayed in the heatmap (incoming signaling). The right colored bar plot represents the sum of the values in rows (outgoing signaling). In the heatmap, red (or blue) represents increased (or decreased) signaling in AR-97Q mice compared to wild-type mice. Relative value = the interaction strength from source to target in AR-97Q mice – the interaction strength from source to target in wild-type mice. **B,** Signaling changes in oligodendrocytes, oligodendrocyte progenitors (OLpro), and oligodendrocyte precursor cells (OPCs) in AR-97Q mice compared to wild-type mice at 13 weeks. The vertical axis represents the differential incoming interaction strength, while the horizontal axis represents the differential outgoing interaction strength. **C,** Bubble plots of the communication probability of all the significant

ligand–receptor interactions between oligodendrocytes and inhibitory neurons or excitatory neurons, which are decreased in AR-97Q mice at 13 weeks. The dot color and size represent the communication probability and p values, respectively. The p values were computed from a one-sided permutation test. The ligand–receptor pair interactions that are decreased at 13 weeks are indicated by red arrows. The ligand–receptor pairs related to NCAM and NRXN are indicated by green arrows. OL, oligodendrocytes; AS, astrocytes; IN, inhibitory neurons; MI, microglia; EX, excitatory neurons; OLpro, oligodendrocyte progenitors; PE, pericytes; EN, endothelial cells; FI, fibroblasts.

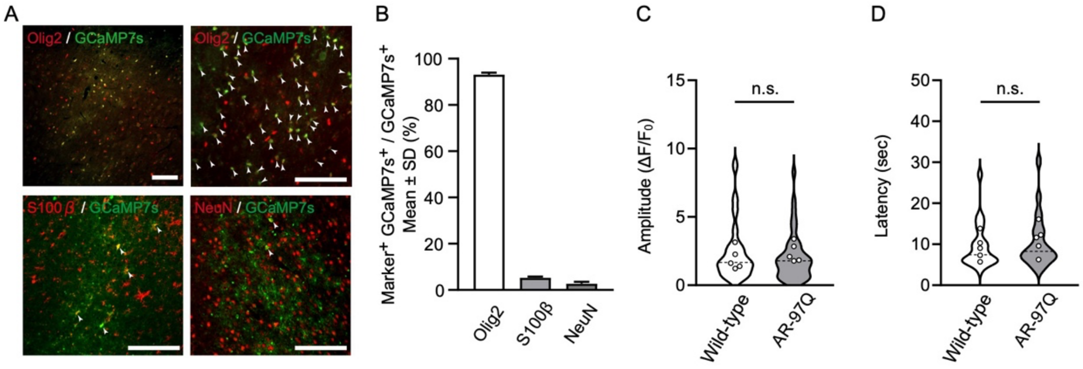

Supplementary Figure 30. Calcium imaging of oligodendrocytes in AR-97Q and wild-type mice

A. Immunofluorescence staining showing the expression of GCaMP7s with cell type markers olig2 (oligodendrocytes), S100β (astrocytes), and NeuN (neurons) in the cerebral cortex of wild type mice at 9 weeks. Arrows indicate GCaMP7s-positive cells co-stained with each marker. **B.** The ratio of GCaMP7s positive cells co-stained with each cell markers (N = 4 mice). **C, D.** Amplitude (**C**) and latency (**D**) of Ca^{2+} events on a process were not significantly changed between the oligodendrocytes from AR-97Q and wild-type mice. Wild-type: N = 69 processes, 20 cells from 5 mice; AR-97Q mice: N = 107 processes, 24 cells from 5 mice, Mann–Whitney U test. Circles on the violin plots indicate individual means of each mouse (Amplitude: p = 0.348; latency: p = 0.460, respectively. Unpaired t-test). Scale bar: 100 μm (**A**).

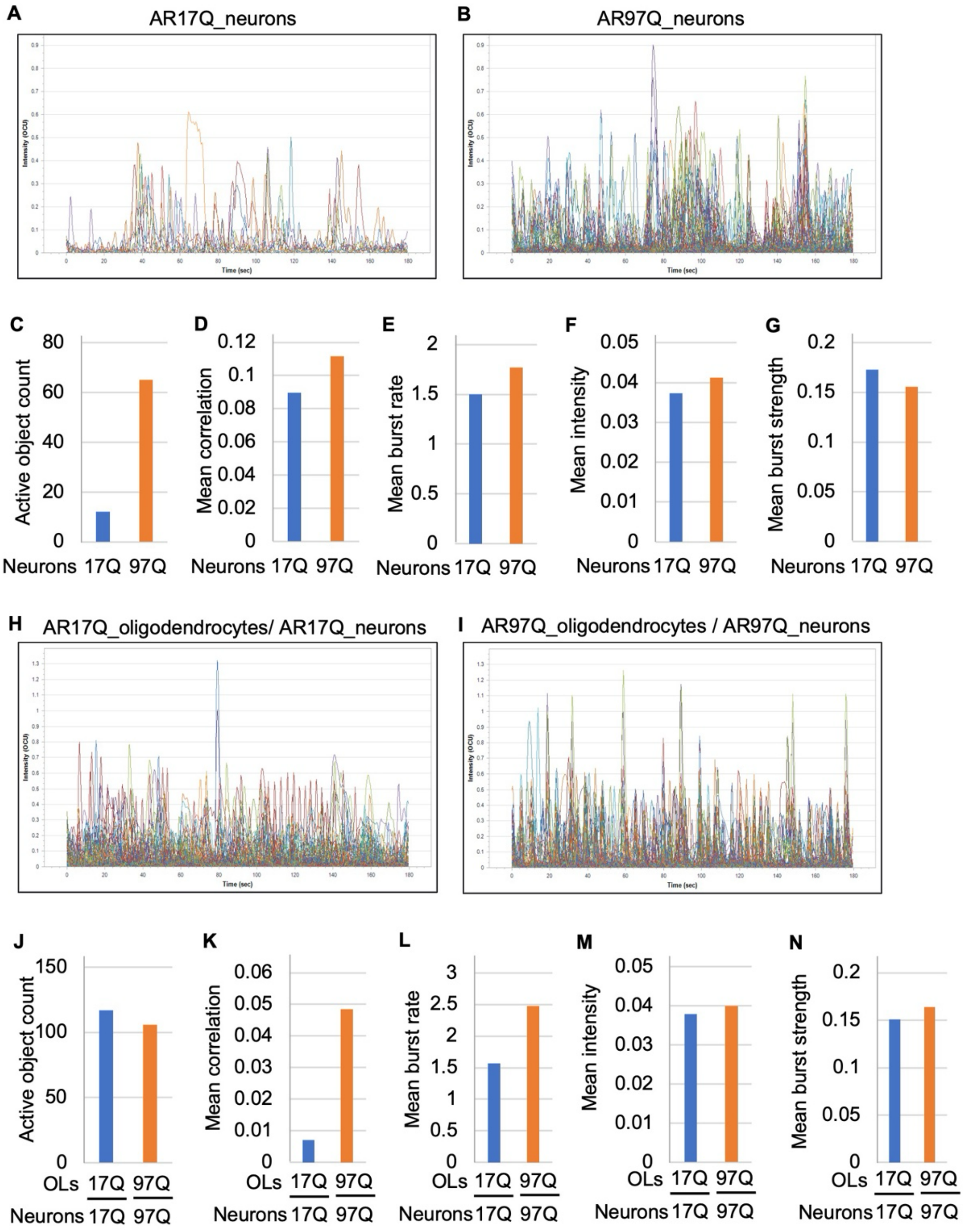

Supplementary Figure 31. Mutant AR in neurons affects neuronal activity

A, B, Calcium imaging traces of AR17Q_neurons (**A**) and AR97Q_neurons (**B**) models. The Y-axis shows the intensity of the signal in the range from 0 to 0.9. **C–G,** Active object count (**C**), mean correlation (**D**), mean burst rate (**E**), mean intensity (**F**), and mean burst strength (**G**) of AR17Q_neurons and AR97Q_neurons models. **H, I,** Calcium imaging traces of the co-culture systems of AR17Q_ oligodendrocytes/ AR17Q_neurons (**H**) and AR97Q_

oligodendrocytes/ AR97Q_neurons (**I**). The Y-axis shows the intensity of the signal in the range from 0 to 1.4. **J–N,** Active object count (**J**), mean correlation (**K**), mean burst rate (**L**), mean intensity (**M**), and mean burst strength (**N**) of the co-culture systems of AR17Q_ oligodendrocytes/ AR17Q_neurons and AR97Q_ oligodendrocytes/ AR97Q_neurons. OLs, oligodendrocytes.

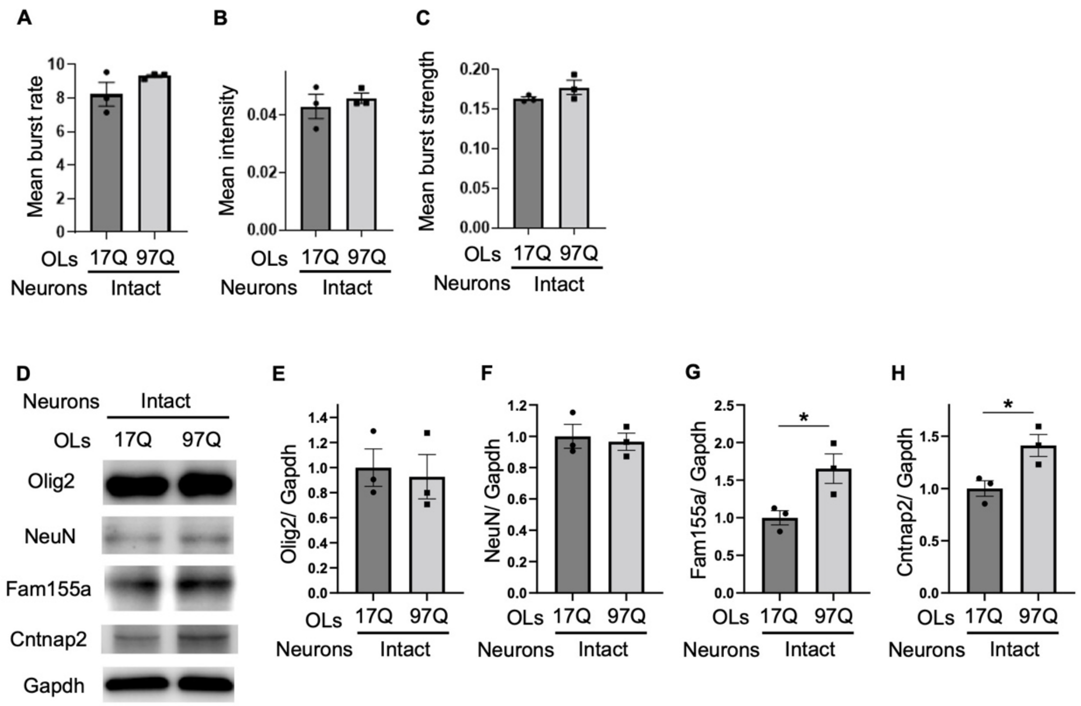

Supplementary Figure 32. The levels of Fam155a and Cntnap2 are significantly increased in the AR97Q_oligodendrocytes/ intact neurons model compared to the AR17Q_oligodendrocytes/ intact neurons model

A–C, Mean burst rate (**A**), mean intensity (**B**), and mean burst strength (**C**) of the co-culture systems of AR17Q_oligodendrocytes/ intact neurons and AR97Q_oligodendrocytes/ intact neurons. **D,** Immunoblotting of Olig2, NeuN, Cntnap2, and Fam155a in AR17Q_oligodendrocytes/ intact neurons and AR97Q_oligodendrocytes/ intact neurons co-culture models. **E–H,** Quantitative immunoblot analysis of Olig2 (**E**), NeuN (**F**), Fam155a (**G**), and Cntnap2 (**H**) in the co-culture systems of AR17Q_oligodendrocytes/ intact neurons and AR97Q_oligodendrocytes/ intact neurons (n = 3 per group). Error bars indicate the SEM. *$p < 0.05$, unpaired two-sided t test. OLs, oligodendrocytes.

Genotyping primers

Mice	Forward (5’-3’)	Reverse (5’-3’)
AR-97Q	CTTCTGGCGTGTGACCGGCG	TGAGCTTGGCTGAATCTTCC

RT-PCR primers

Gene	Forward (5’-3’)	Reverse (5’-3’)
Asic2	CAGGAGTGAAGGTTCAGATCCA	GGAAAGAAGTCGAGTCCCATCTC
Cntnap2	CCTTGGCACCTAGATCACTTG	GCGATGACCCCTCCAATGATA
Grip1	CTTGGCCTGACGGTATCGG	CCGCCTTGATGTAGTCGCC
β2mg	CTGACCGGCCTGTATGCTAT	CCGTTCTTCAGCATTTGGAT

Supplementary Table 1. List of used primers

Supplementary Methods

Pseudotime analysis of cell differentiation trajectories

The Monocle3 package (v1.3.1) (1) was used for pseudotime trajectory analysis to determine the differentiation status of cells at different disease stages.

Cell–cell interaction analysis

Seurat preprocessed data were subjected to the CellChat package (v1.6.1) with the Seurat R package (v4.3.1) to infer, analyze, and visualize cell–cell communication (2). The ligand–receptor interaction database was included in the package.

Protein–Protein Interaction Analysis

To determine the core DEGs at each age, we used the online tool STRING (version 12.0) (http://string-db.org/) to construct PPI networks, and the parameters were set to the default value.

Gene co-expression network analysis

To generate robust gene co-expression network for the scRNA-seq data, we applied hdWGCNA (version 0.4.0) (3). We extracted each of oligodendrocytes and OPCs from the

merged scRNA-seq data of wild-type and AR-97Q mice at weeks 6 and 13, and constructed a gene co-expression network. We applied the signed network to construct the co-expression networks. Based on the WGCNA tree, we generated modules of highly correlated genes that included at least 50 genes. Next, module-eigengenes (MEs) that explain the expression patterns of modules were calculated. To summarize the gene signatures in each module, a module score for each co-expression module using UCell R package (v2.8.0) (4). To evaluate the module differences between the wild-type and AR-97Q mice at each week for each of the oligodendrocytes and OPCs, the Wilcoxon rank sum test was applied. Finally, enrichment analysis was performed on each of modules that show the most significant difference between wild-type and AR-97Q mice in each of cell types using the enrichR R package (v3.2) (5), focusing on the biological process category of Gene Ontology terms.

Cell culture and the generation of SBMA oligodendrocyte model cells

Tissue culture media and reagents were purchased from Invitrogen. Oli-neu cells were kindly provided by Prof. J. Trotter (University of Mainz, Mainz, Germany) and cultured in Sato medium containing Dulbecco's modified Eagle's medium (DMEM) (#08458-16, nacalai tesque, Kyoto, Japan) supplemented with 1% (v/v) horse serum, insulin (5 µg/ml), penicillin (100 U/ml), streptomycin (0.1 mg/ml), 1% (v/v) N2 supplement, sodium selenite (190 nM),

gentamicin (25 μg/ml), triiodothyronine (400 nM) and L-thyroxine (520 nM). Cells were kept in a humidified atmosphere with 5% CO2 at 37°C. Lentiviruses expressing full-length human AR containing 17 or 97 CAG repeats were introduced into Oli-neu cells on day 2 of cell culture, thereby generating cell lines harboring AR-17Q as control cells and cell lines harboring AR-97Q as SBMA model cells. Differentiation was induced by adding 1 μM PD174265 (Calbiochem, CA, USA) to the medium on day 2 (6). On day 3, the cells were treated with 10nM DHT. Total RNA was extracted from the cells on day 5. We performed four passages of these cells before analysis.

Lentiviral production

Lentivirus was prepared following Campeu's protocols (7). Briefly, lentiviral particles were produced in HEK293T cells by transfection using Lipofectamine 2000 (#11668030, Thermo Fisher Scientific). Lentivirus-containing supernatant was collected at 72 hours after transfection and stored at -80°C. The viral titer was measured using a Lenti-X qRT–PCR Titration Kit (#631235, TaKaRa, Shiga, Japan).

Bulk RNA-seq

Total RNA was extracted from the SBMA oligodendrocyte cell model and the control cell model using the RNeasy Mini Kit (Qiagen, Hilden, Germany). The quality and quantity of the RNA obtained were checked using a NanoDrop 2000 spectrophotometer (Thermo Fisher Scientific) and analyzed with the Agilent 2100 Bioanalyzer system (Agilent Technologies Inc.). The RNA was sent to Macrogen (Macrogen Inc., Seoul, South Korea) for library preparation and sequencing. The sequencing of the 8 libraries was carried out using the NovaSeq sequencing protocol and TruSeq stranded mRNA Library Kit, following a paired-end 100 bp strategy on the Illumina NovaSeq 6000 platform.

Quantitative RT–PCR

Total RNA was extracted from the cells using the RNeasy Mini Kit (Qiagen). The extracted RNA was then reverse-transcribed into first-strand cDNA using the ReverTra Ace qPCR RT Kit (Toyobo, Osaka, Japan). RT–PCR was performed using a KOD SYBR qPCR kit (Toyobo), and the amplified products were detected with a CFX Connect system (Bio-Rad Laboratories, Hercules, CA, USA). The reaction conditions were as follows: 98.0 °C for 2 min, 45 cycles of 10 s at 98.0 °C, 10 s at 60.0 °C and 30 s at 68.0 °C. The expression level of the internal control, β_2 microglobulin, was simultaneously quantified. The primers are listed in Supplementary Table 1.

Immunoblotting

Cultured cells were lysed in buffer containing 50 mM Tris-HCl (pH 8.0), 150 mM NaCl, 1% Nonidet P-40, 0.5% deoxycholate, 0.1% SDS, and 1 mM 2-mercaptoethanol with the Halt Protease and Phosphatase Inhibitor Cocktail (Thermo Fisher Scientific). We separated equal amounts of protein on 5-20% SDS–PAGE gels (Wako, Osaka, Japan) and transferred them to Hybond-P membranes (GE Healthcare, Piscataway, NJ, USA). The following primary antibodies and dilutions were used: AR (#5153, 1:2000; Cell Signaling Technology), Mog (ab233549, 1:2000; Abcam), PDGFRα (#3164S, 1:2000; Cell Signaling Technology), Sox10 (sc-365692, 1:200; Santa Cruz), Olig2 (ab109186, 1:1000, Abcam), NeuN (MAB377, 1:1000, Millipore), Cntnap2 (PA5-28969, 1:500, Invitrogen), Fam155a (bs-8223R, 1:500, Bioss), APC (ab58, 1:1000, Abcam), and Asic2 (ASC-012, 1:1000, alomone labs). Primary antibodies bound to the proteins were probed with a 1:5000 dilution of horseradish peroxidase-conjugated secondary antibodies, and the bands were detected using an immunoreaction enhancing solution (Can Get Signal; Toyobo) and enhanced chemiluminescence (ECL Prime; GE Healthcare). Chemiluminescence signals were digitized using a ChemiDoc MP imaging system (Bio-Rad Laboratories). Membranes were reprobed with an anti-GAPDH antibody (ab8245, 1:5000; Abcam) for normalization.

Immunohistochemistry

Mouse tissues were dissected, postfixed in 10% phosphate buffered formalin, and paraffin-embedded. Six-micron-thick sections were prepared from the paraffin-embedded tissues. The sections designated to be stained with the anti-polyglutamine antibody (1C2) (MAB1574, 1:20,000; Millipore) were treated with formic acid for 5 min at room temperature. The sections designated to be incubated with the human AR antibody (#5153, 1:1000, Cell Signaling Technology) were boiled in 10 mM citrate buffer for 15 min. Primary antibodies bound to proteins were incubated with a secondary antibody labeled with a polymer as part of the Envision + system containing horseradish peroxidase (Dako Cytomation, Gostrup, Denmark). Images of immunohistochemically stained sections were obtained using an optical microscope (BX51, Olympus, Tokyo, Japan). Immunoreactivity and cell size were analyzed using ImageJ software (NIH, Bethesda, MD). The mean ± standard error of the mean (SEM) of the obtained values is presented in arbitrary units.

Immunofluorescence for mouse tissues and human autopsy samples

Mouse tissues and autopsied human spinal cords were dissected, postfixed with 10% phosphate-buffered formalin and processed for paraffin embedding. Six-micron-thick sections

were prepared from paraffin-embedded tissues. The sections designated to be stained with the anti-polyglutamine antibody (1C2) (MAB1574, 1:20,000; Millipore) were treated with formic acid for 5 min at room temperature. The sections designated to be incubated with the anti-human AR (554224, 1:100; Biosciences), Plp1 (ab28486, 1:500; Abcam), Olig2 (#18953, 1:100; IBL), Sox10 (MAB2864, 1:50; R&D for human samples, sc-365692, 1:200; Santacruz for mouse samples), Mbp (ab7349, 1:200; Abcam), Asic2 (ASC-012, 1:100; alomone labs), Fam155a (orb2222, 1:100; biorbyt), and GFAP (ab7260, 1:500; Abcam) antibodies were boiled in 10 mM citrate buffer for 15 min. After washing, the samples were incubated with Alexa-488–conjugated donkey anti-mouse IgG (1:1000; A21202, Invitrogen), Alexa-546–conjugated goat anti-mouse IgG (1:1000; A11003, Invitrogen), Alexa-488–conjugated goat anti-rabbit IgG (1:1000; A11008, Invitrogen), or Alexa-568–conjugated goat anti-rabbit IgG (1:1000; A11036, Invitrogen) for 1 hour, stained with Hoechst 33342 (H3570, Invitrogen), mounted using ProLong Diamond Antifade Mountant (P36961, Invitrogen), then imaged with a fluorescence microscope (BZ-X810, Keyence Corporation, Osaka, Japan). The quantification of fluorescence intensities was performed using Image J software.

G-ratio measurement of the L5 ventral roots and L5 anterior columns

The L5 ventral roots and spinal cords at the level of L5 were dissected from wild-type and AR-97Q mice at 13 weeks. The nerve tissues were fixed in 2.5% glutaraldehyde in a 0.125 M cacodylate buffer (pH 7.4) and then embedded in epoxy resin. The specimens were cut into semithin sections and stained with toluidine blue. To determine the g-ratios, we assessed at least 100 randomly selected fibres in the images of toluidine blue-stained sections (3 animals/group). G ratio analysis was performed according to MyelTracer software v1.3 (8).

Cell co-culture of primary oligodendrocytes and primary neurons

Primary cortical neurons were obtained from Wister rat cerebrum at E17. Briefly, the cerebri of six embryos were collected in a petri dish containing 1× HBSS. After carefully removing the meninges, the tissue was transferred to a 15 ml centrifuge tube containing 10 ml 0.25% Trypsin-EDTA (Gibco, 25200-056) and incubated at 37°C for 30 minutes. Trypsin was discarded and the enzymatic reaction was stopped by mixing the tissue with 3 ml DMEM supplemented with 10% FBS and DNase-I (0.2 mg/ml) (11284932001, Roche Diagnostics, Mannheim, Germany). The tissue suspension was incubated at 37°C for 10 minutes and centrifuged at 300×g for 10 minutes. The total number of cells was counted and the cells were seeded onto poly-L-lysine-coated coverslips at a density of 0.4 x 10^5 /ml. After 2 hours of adhesion, the plating medium was carefully aspirated and Neurobasal medium (500ml

Neurobasal medium (Gibco, 21103049), 10ml B27 (Gibco, 17504-044), 1x GlutaMAX™ (Gibco, 35050-061), and 20 units/ml penicillin–streptomycin) was slowly added to the wells. The day of the primary culture was defined as day 1 in vitro (DIV1). The medium was changed every three days by replacing half of the medium with fresh medium. At DIV5, neurons were infected with lentivirus expressing AR17Q or AR97Q. Neurons were then cultured for an additional 2 days.

Primary glial cells were obtained from the cerebrum of Wister rat at postnatal day 1. The cerebri of six embryos were collected in a petri dish containing 1× HBSS. After carefully removing the meninges, the cerebral tissue was cut into small pieces using a surgical blade. The tissue was transferred to a 15 ml centrifuge tube containing 13.6 ml of HBSS, DNase I (0.2 mg/ml), and 0.8 ml of 0.25% Trypsin-EDTA and incubated at 37℃ for 15 minutes. The tissue was titrated 10 times with a 1 ml pipette tip. The enzymatic reaction was stopped by adding 5 ml of DMEM20S (500ml of DMEM, 1x GlutaMAX™, 1mM Sodium pyruvate, 20% FBS, and 50 units/ml penicillin–streptomycin) and the tissue suspension was centrifuged at 100×g for 5 minutes. The supernatant was removed and the pellet was gently resuspended in 20 ml of DMEM20S and incubated on ice for 10 minutes. The supernatant was then passed through a 70 μm cell strainer and stored until the next step. The pellet was gently resuspended in 20 ml of DMEM20S again and incubated on ice for 10 minutes. The

supernatant was passed through a 70 µm cell strainer. DMEM20S was added to the supernatant until it reached 60 ml (10 ml/ an embryo) and incubated for 14 days. The medium was changed every three days by replacing half of the medium with fresh medium. At day 12 in vitro (DIV12), glial cells were infected with lentivirus expressing AR17Q or AR97Q. At DIV14, the sealed cell culture flasks were placed on an orbital shaker at 200 rpm for 1 hour at 37°C. The culture medium was removed and replaced with 10 ml of fresh DMEM20S. Then, the sealed cell culture flasks were placed on an orbital shaker at 200 rpm at 37°C overnight. On the next day, the culture medium was transferred to a petri dish and left for 45 minutes. Then, the medium was passed through a 40 µm cell strainer and centrifuged at 100×g for 10 minutes. The supernatant was discarded and the cells were resuspended to a density of 0.4 x 10^5/ml with co-culture medium (500 ml DMEM, 1% horse serum (Gibco, 16050122), 5 ug/ml insulin (093-06351, Wako), 10 units/ml penicillin–streptomycin, 1% N2 supplement (17502048, Gibco), 190 nM sodium selenite (10102-18-8, Millipore), 400 nM triiodothyronine (55-06-1, Wako), 520 nM L-thyroxine (T1775, Millipore), 10 ml B27, 10 ng/ml PDGF-AA (165-25541, Wako), and 5 ng/ml FGF (PHG0367, Thermo Fisher Scientific)) and seeded onto primary cortical neurons at DIV 7. PDGF-AA and FGF were added before use. The co-culture medium was changed every three days by replacing half of the medium with fresh medium.

Immunocytochemistry

Cells were cultured on coverslips, fixed with 2% paraformaldehyde buffer for 20 minutes, and incubated with 0.2 % Triton X-100 (9036-19-5, Sigma-Aldrich) and phosphate-buffered saline (PBS) for 5 minutes. The washed cells were then blocked with Tris-NaCl-blocking (TNB) buffer (FP1012, PerkinElmer) for 45 minutes and incubated overnight at 4°C with the primary antibodies: Tuj1 (ab18207, 1:1000; Abcam) and Mbp (ab7349, 1:500; Abcam). After further washing, the cells were incubated with the secondary antibody for 30 min and mounted with ProLong Gold Antifade Reagent with DAPI (P36935; Thermo Fisher Scientific). Confocal images were taken using ZEN (black edition) 2.1 (Carl Zeiss) on a confocal system (LSM880; Carl Zeiss) with an objective Plan-Apochromat 40×/1.4 oil immersion objective. Images are acquired using ZEN (black edition) 2.3 software. As secondary antibodies, AlexaFluor goat anti-rabbit IgG 488 (A11008, 1:1000; Thermo Fisher Scientific) and goat anti-rat IgG H&L (Cy5) (ab6565, 1:1000; Abcam) were used.

Construction of adeno-associated virus (AAV)

To transduce a fluorescent Ca^{2+} indicator (GCaMP7s) into oligodendrocytes, we construct an oligodendrocyte preferring AAV. A promoter region of the myelin-associated glycoprotein gene (MAG), a 1.5-kb fragment of genomic DNA corresponding to the 5’ flanking region of

translation initiation site of exon1 (9, 10), was amplified by PCR with the following primer set. Forward: 5'-TGCTCTAGGAAGATCT (BglII restriction site) -CGACTCCAGCTCCAACTAGG; Reverse: 5'-CTAGCAGCTTGAATTC (EcoRI restriction site) -GCCCCCACTTGCCAGCCCCTCCCCT. The genomic DNA prepared from human cancer cell line (HepG2) was used as template. The PCR fragment was subcloned into pBSKS II(+) following sequencing, we confirmed that the sequence was completely matched with those in published data base (PubMed, National Library of Medicine, NIH). The human MAG promoter fragment (hMAG1.5kb) was extracted from pBSKS II(+) at BglII – EcoRI site and cloned into BglII – EcoRI site of the pGP-AAV-hSyn-GCaMP7s-WPRE plasmid [addgene #104487], then yielded the hMAG promoter driven GCaMP7s viral plasmid (pAAV-hMAG1.5-GCaMP7s-WPRE). To generate AAV vector, the virus plasmid was transfected with the packaging plasmid and helper plasmid into HEK293T cell and cultured for 4 days at 37℃. The culture medium grown the transfected HEK293T cell was collected, and the AAV in the medium was concentrated by ultracentrifugation and purified by an ultrafiltration (Vivaflow 50, Sartorus, Goettingen), as described previously (11). The tighter of AAV solution was determined as viral genomes per solution (vg/ml) by the qPCR with the following primer set. Forward: 5'-CGCTATGTGGATACGCTGCT; Reverse 5'-CGGGCCACAACTCCTCATAA, and

then stored at -80℃ deep freezer. The specificity of hMAG1.5kb promoter has evaluated in Supplementary Figure 29.

Surgery and adeno associated virus injection

Under ketamine (74mg/kg, i.p.) and xylazine (10mg/kg, i.p.) anesthesia, the skin was disinfected using 70% (w/v) ethanol. The skull was exposed, cleaned, and a custom-designed metal plate was firmly affixed to the skull using dental cement (C-CEM ONE; GC, Tokyo, Japan). To protect the intact skull from drying, it was coated with an acrylic-based dental resin (Super bond; Sun Medical, Shiga, Japan). This metal plate provided stable fixation for the mice during subsequent procedures such as craniotomy, AAV injection, and two-photon imaging. One or two days after plate implantation, craniotomy (circular, 2.5mm diameter) and AAV injection were conducted under 1.0% isoflurane inhalation. The position of the cranial window was stereotactically determined based on the mouse brain atlas, centered at 0.8 mm anterior and 1.2 mm lateral to the bregma. After craniotomy, a total of 1 µl AAV (AAV8-hMAG-GCaMP7s-WPRE, 4.1 x 10^{12} viral genome/ml) solution was injected through a glass pipette (tip diameter, 25-30 µm). The exposed brain surface was covered with 2% (w/v) agarose L (Nippon Gene, Tokyo, Japan) dissolved in saline, and sealed with a glass window consisting of two coverslips (2.0mm [square] and 4.5mm [round] in diameter; Matsunami,

Osaka, Japan) bounded with ultraviolet light-polymerized adhesive (NOR-61, Norland Product, Cranbury, NJ). The edges of the glass window were sealed and fixed with an ultraviolet light-polymerized adhesive and dental cement.

Two-photon imaging

Two-photon images were acquired from the left cerebral cortices using a laser scanning system (C2 plus, Nikon, Japan) equipped with a water-immersion objective lens (20×, numerical aperture [N.A.] = 1.0, Nikon, Japan). For two-photon imaging based on C2 plus excitation light beams, an aTi: sapphire laser (Coherent, Santa Clara, CA) operating at a wavelength of 920–950nm was used. The imaging fields were 145 × 145 μm (objective lens 20×, digital zoom 3.5) at a depth of 200–300 μm below the brain surface. The scanning speed was 500 ms per frame. Continuous 1,000-frame images were acquired for each image field with no interval time.

Imaging analysis

Images were analyzed using ImageJ (National Institute of Health) and MATLAB software packages (Math Works, Natick, MA). Displacement of XY-plane within the 1,000-frame images due to mouse motion was corrected for focal plane shift using the ImageJ TurboReg

plug-ins. To estimate Ca^{2+} activity in oligodendrocyte processes, regions of interest were determined using non-negative matrix factorization (12). To detect and analyze Ca^{2+} transients, baseline fluorescence was defined as the 35th percentile of the total fluorescence intensity histogram obtained during all imaging periods (F0). Ca^{2+} transients were calculated using the equation ΔF/F0 (ΔF=F-F0), where ΔF is the instantaneous fluorescence signal and ΔF exceeded 4 standard deviations (SDs) of the baseline fluorescence (F0). We used an F0 set at the 35th percentile of the total fluorescence distribution when re-analyzing the results. The frequency of occurrence of Ca^{2+} transients was calculated as the ratio of the total number of transients over all the imaging periods. The intensity of each Ca^{2+} transient (ΔF/F0) was then calculated from the area under the curve (AUC), which was calculated by integrating the area between the traces representing Ca^{2+} transients and a horizontal line expressing baseline fluorescence. Amplitude was calculated as the maximum ΔF/F0 of each Ca^{2+} transient. Latency was calculated as the duration between the occurrence of the first Ca^{2+} transient that exceeded and was less than 4 SDs of baseline fluorescence.

Calcium imaging analyses

At DIV5, the primary neurons were infected with a lentivirus expressing a genetically-encoded calcium indicator (4761, Sartorius, Germany). Calcium imaging was conducted using

the IncuCyte SX5 (Sartorius, Germany). Scans were taken at least 24 hours after a media change. Six parameters of neural activity were automatically calculated by the Incucyte system. Parameters included the number of active objects (cells), object intensity, correlation, burst duration, and burst strength. The mean correlation is a measure of network synchronicity, with highly synchronous bursting assigned a value of 1 and random bursting a value of -1.

Supplementary References

1. Trapnell C, et al. The dynamics and regulators of cell fate decisions are revealed by pseudotemporal ordering of single cells. *Nat Biotechnol*. 2014;32(4):381–386.
2. Jin S, et al. Inference and analysis of cell-cell communication using CellChat. *Nat Commun*. 2021;12(1). https://doi.org/10.1038/s41467-021-21246-9.
3. Morabito S, et al. hdWGCNA identifies co-expression networks in high-dimensional transcriptomics data. *Cell Reports Methods*. 2023;3(6). https://doi.org/10.1016/j.crmeth.2023.100498.
4. Andreatta M, Carmona SJ. UCell: Robust and scalable single-cell gene signature scoring. *Comput Struct Biotechnol J*. 2021;19:3796–3798.
5. Kuleshov M V., et al. Enrichr: a comprehensive gene set enrichment analysis web server 2016 update. *Nucleic Acids Res*. 2016;44(1):W90–W97.
6. Naffaa V, et al. Bisphenol A Impairs Lipid Remodeling Accompanying Cell Differentiation in the Oligodendroglial Cell Line Oli-Neu. *Molecules*. 2022;27(7). https://doi.org/10.3390/molecules27072274.
7. Campeau E, et al. A versatile viral system for expression and depletion of proteins in mammalian cells. *PLoS One*. 2009;4(8). https://doi.org/10.1371/journal.pone.0006529.
8. Kaiser T, et al. Myeltracer: A semi-automated software for myelin g-ratio quantification. *eNeuro*. 2021;8(4). https://doi.org/10.1523/ENEURO.0558-20.2021.

9. von Jonquieres G, et al. Recombinant human myelin-associated glycoprotein promoter drives selective AAV-mediated transgene expression in oligodendrocytes. *Front Mol Neurosci*. 2016;9(FEB). https://doi.org/10.3389/fnmol.2016.00013.

10. Kato D, et al. Regulation of lipid synthesis in myelin modulates neural activity and is required for motor learning. *Glia*. 2023;71(11):2591–2608.

11. Kobayashi K, et al. Survival of corticostriatal neurons by Rho/Rho-kinase signaling pathway. *Neurosci Lett*. 2016;630:45–52.

12. Yoshida K, et al. Activity-dependent oligodendrocyte calcium dynamics and their changes in Alzheimer's disease. *Front Cell Neurosci*. 2023;17. https://doi.org/10.3389/fncel.2023.1154196.
