# Supplementary material for: Dysregulated synaptic gene expression in oligodendrocytes of spinal and bulbar muscular atrophy
Source: JCI Insight. 2025 Jun 23;10(12):e182123. doi: 10.1172/jci.insight.182123 (PMC12220948; doi:10.1172/jci.insight.182123)

Full unedited blot for Figure 2C

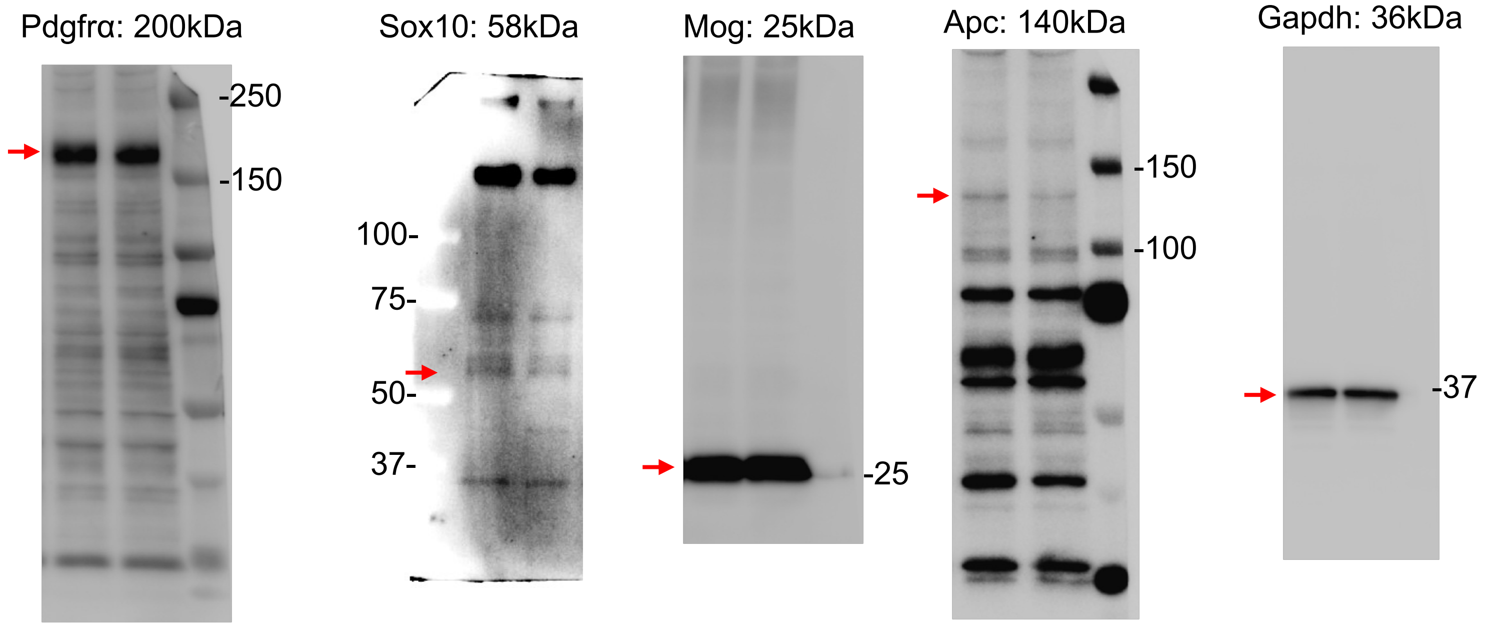

Full unedited blot for Figure 2I

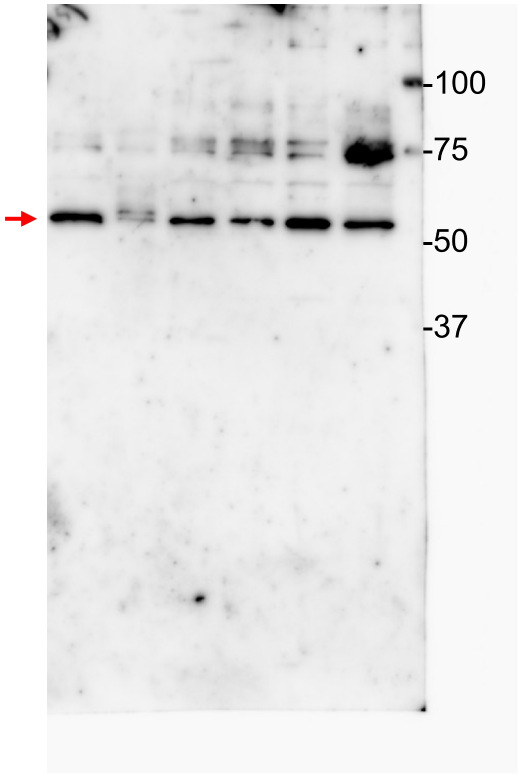

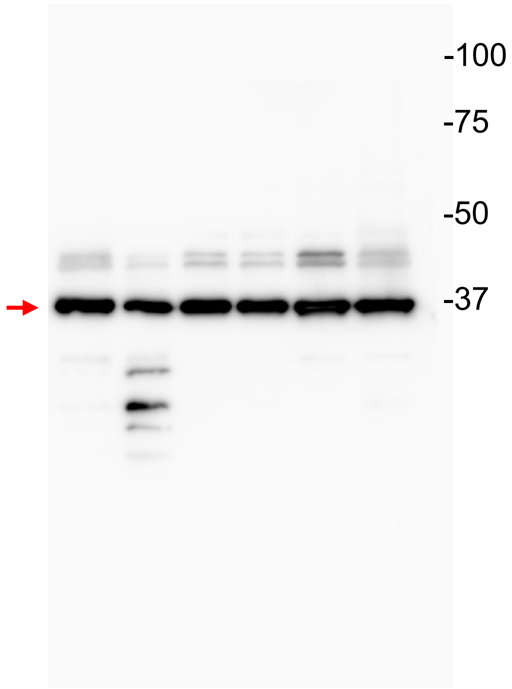

Full unedited blot for Figure 7A

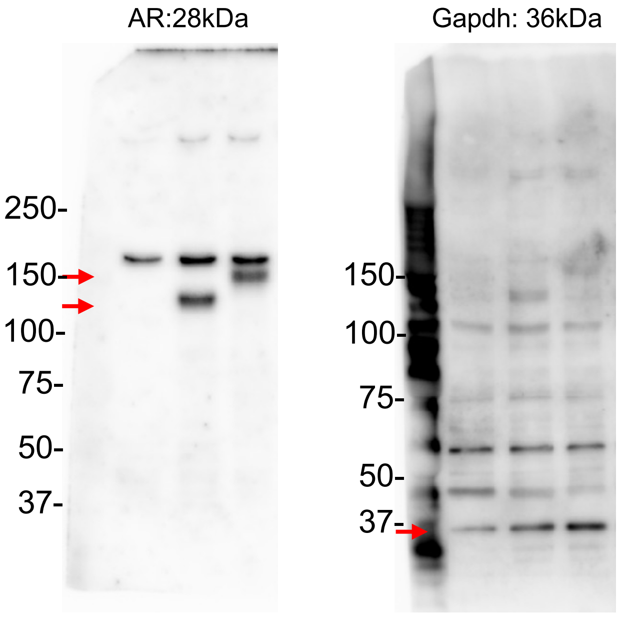

Full unedited blot for Figure 7B

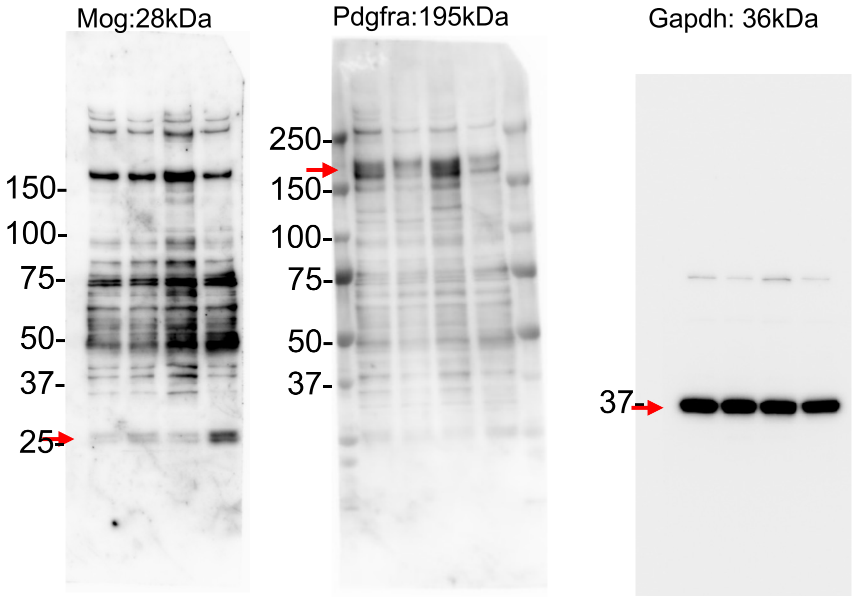

Full unedited blot for Supplementary Figure 16A

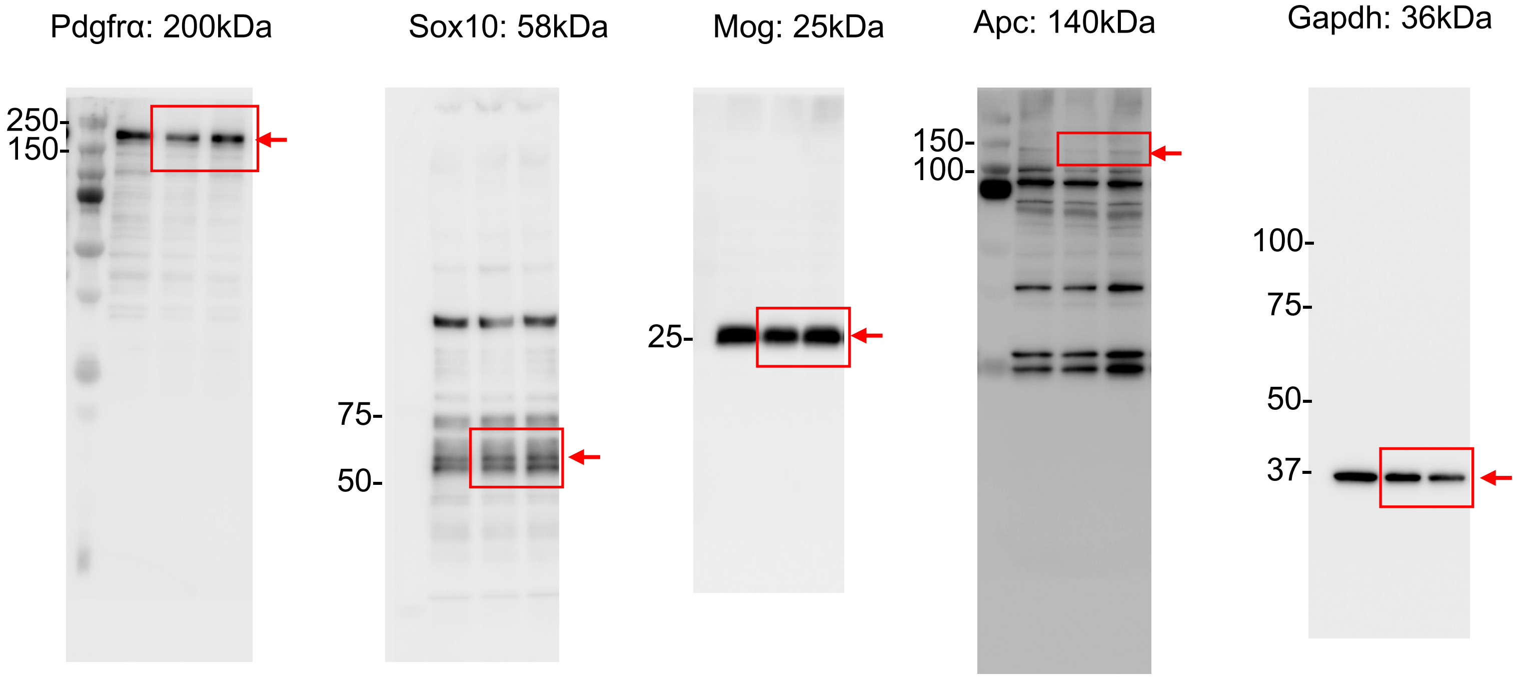

Full unedited blot for Supplementary Figure 17A

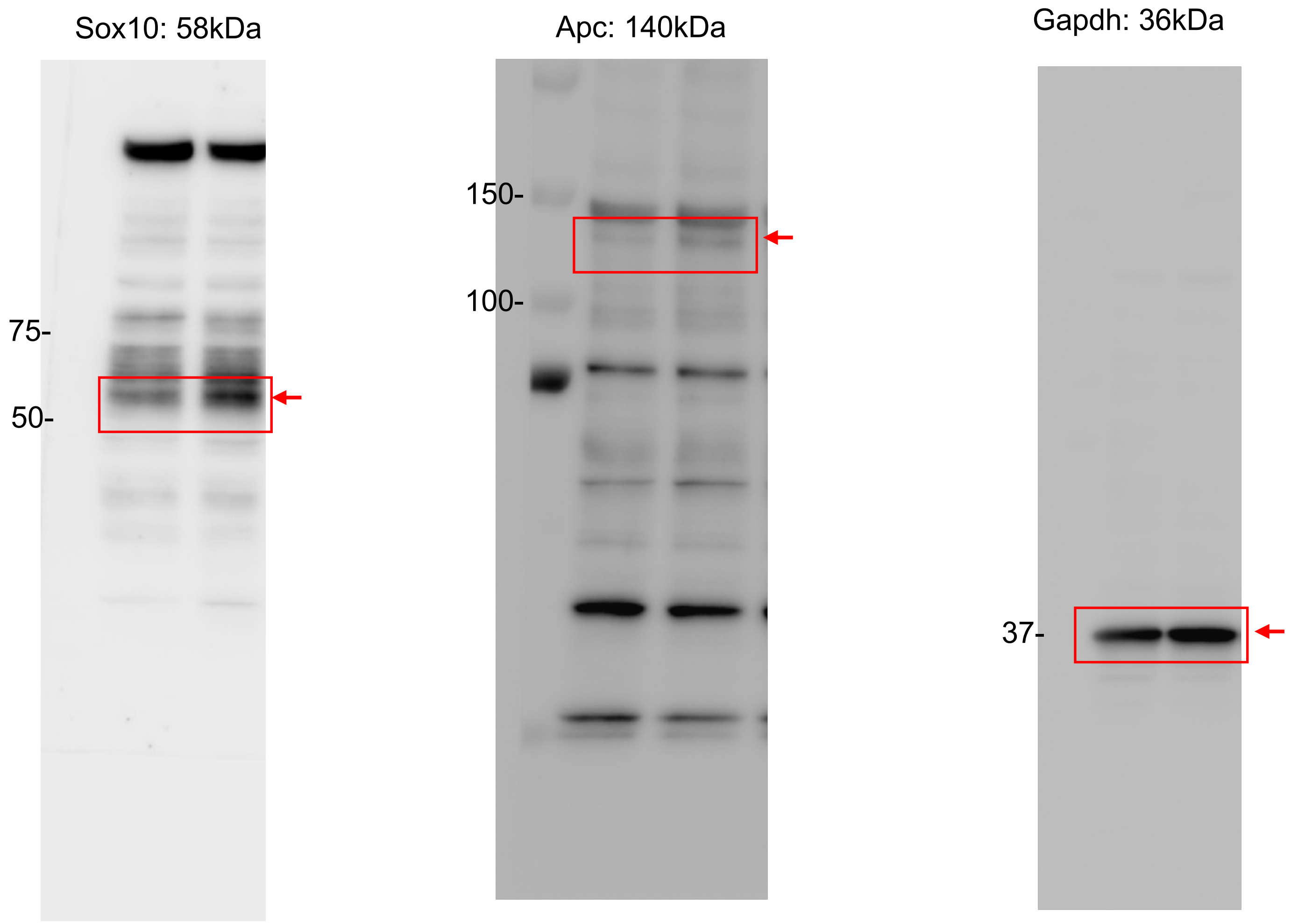

Full unedited blot for Supplementary Figure 17D

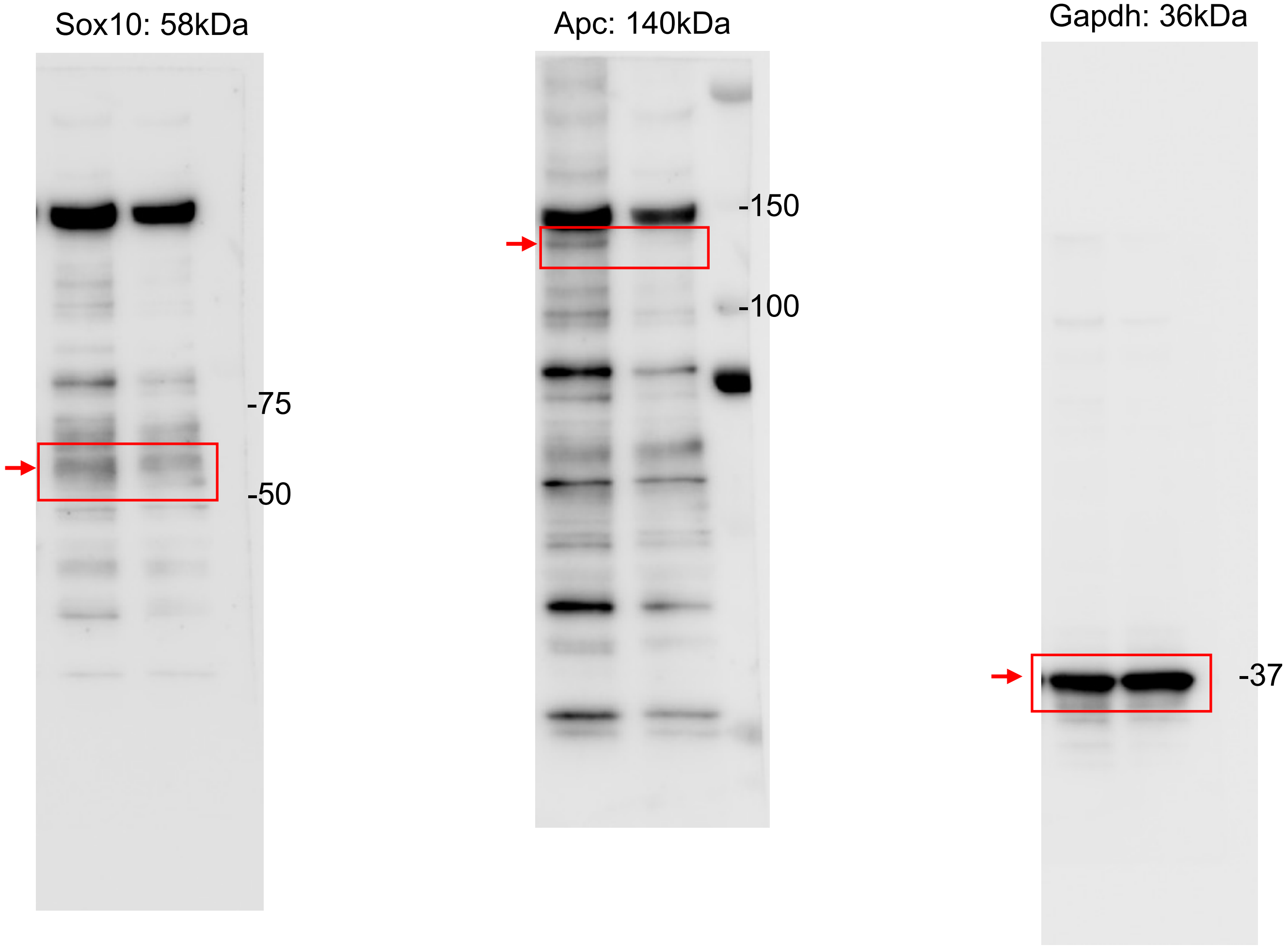

Full unedited blot for Supplementary Figure 18E

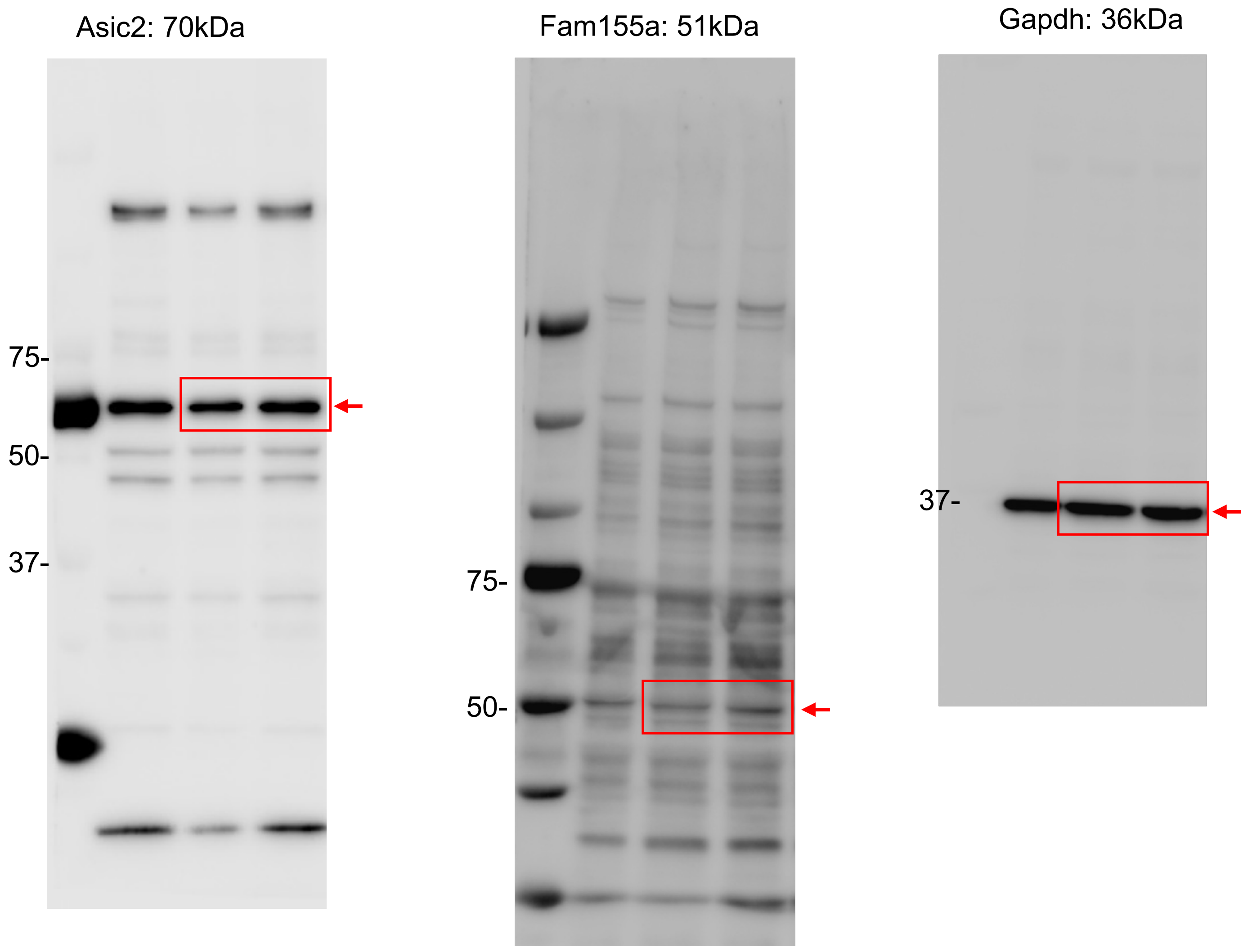

Full unedited blot for Supplementary Figure 18H

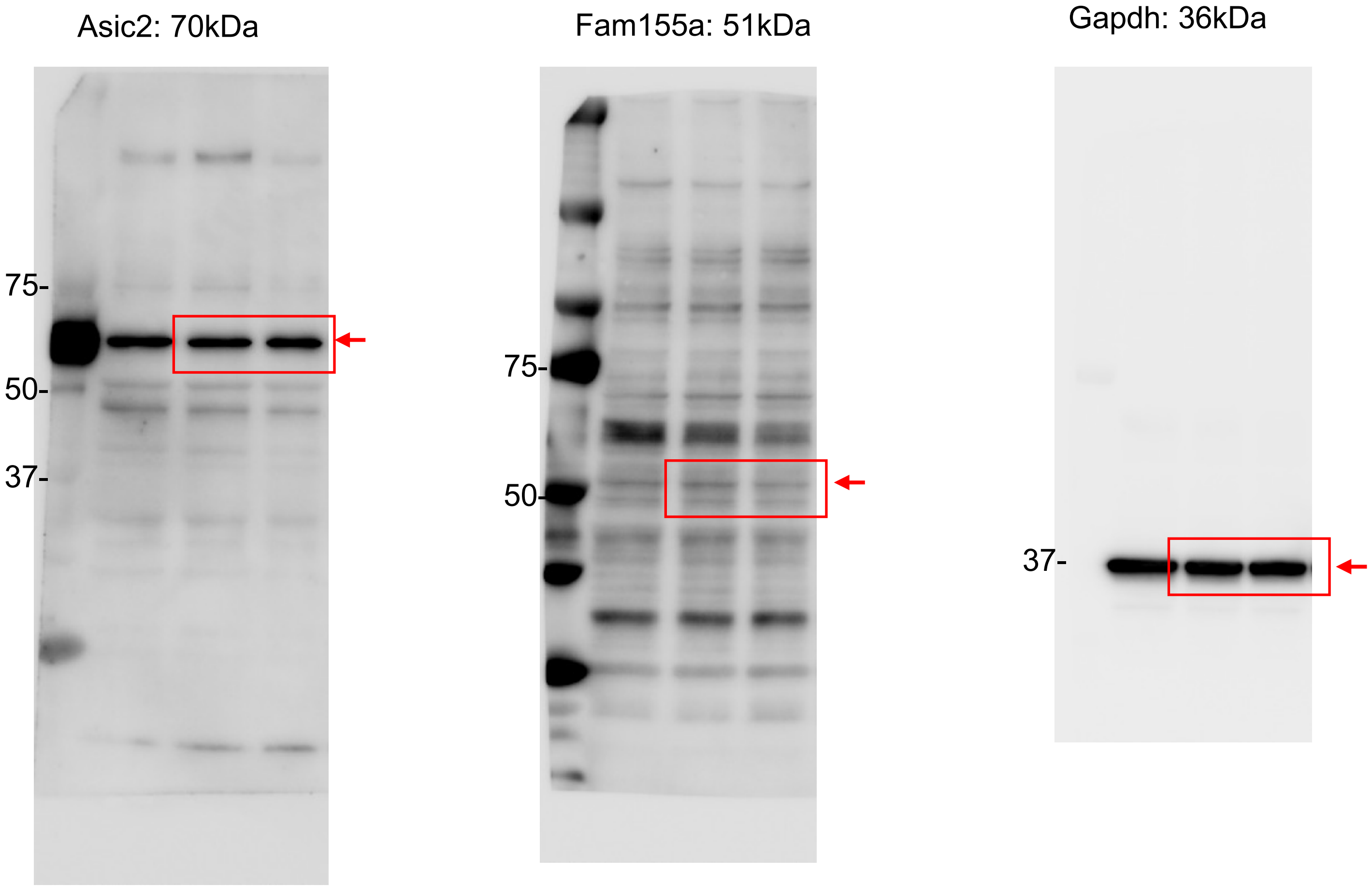

Full unedited blot for Supplementary Figure 18K

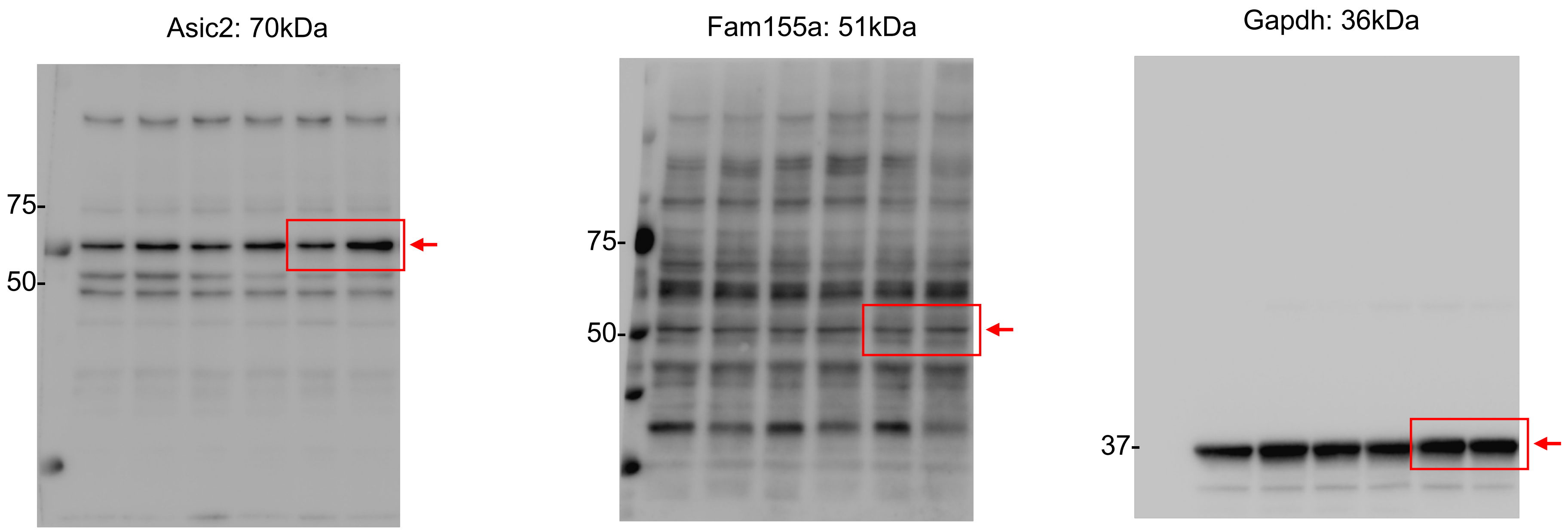

Full unedited blot for Supplementary Figure 22A

ASIC2: 70kDa

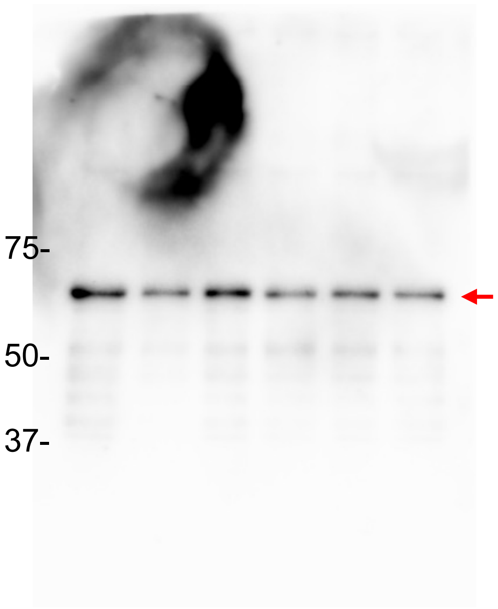

FAM155A: 51kDa

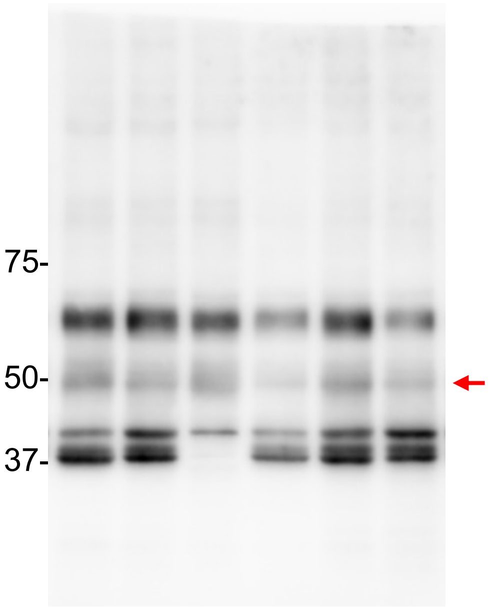

GAPDH: 36kDa

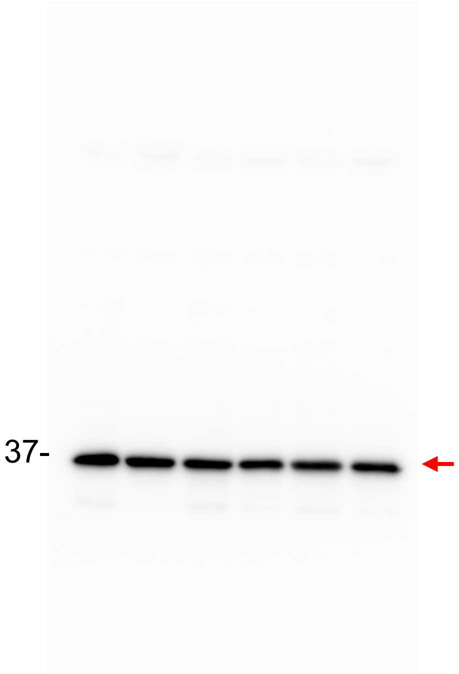

Full unedited blot for Supplementary Figure 32D

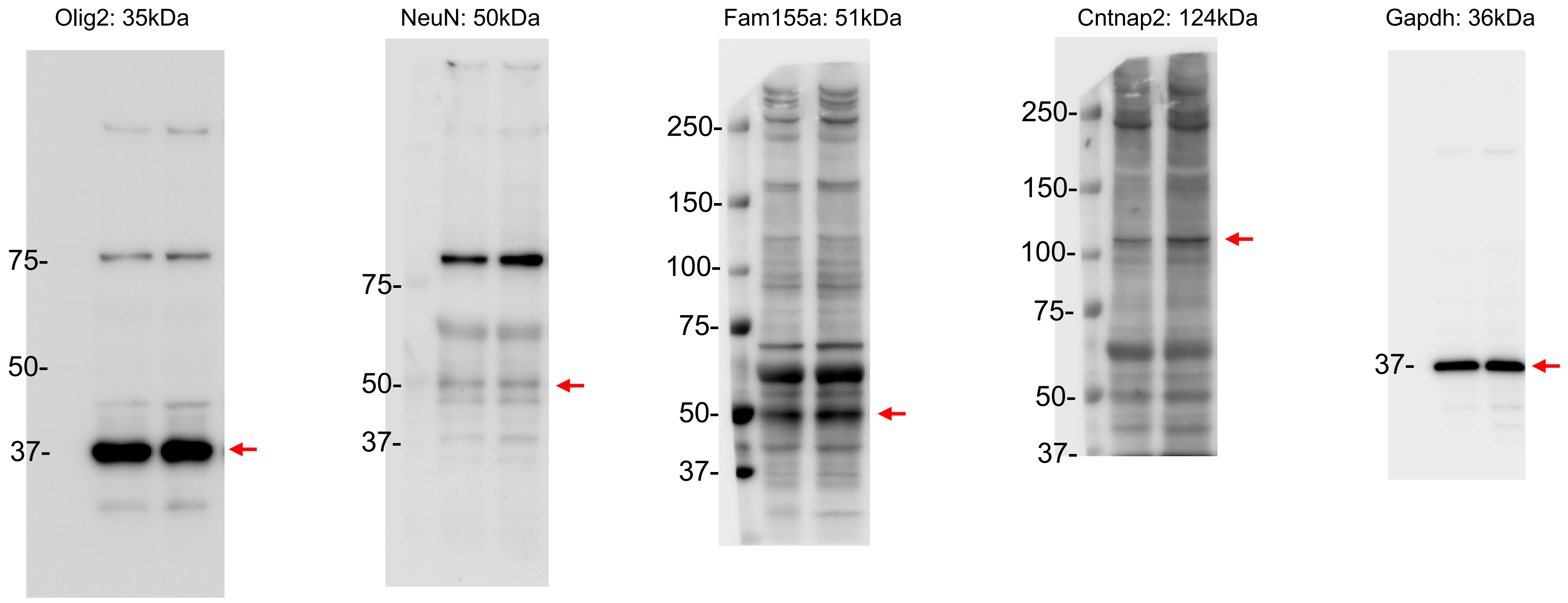

Supplement: Unedited blot and gel images [file jciinsight-10-182123-s031.pdf]
